# Tuning Aggregation in Liquid‐Crystalline Squaraine Chromophores

**DOI:** 10.1002/advs.202416249

**Published:** 2025-03-27

**Authors:** Tianyi Tan, Yu Cao, Changlong Chen, Sanliang Ling, Gaolei Hou, Martin J. Paterson, Xin Wang, Ying Chen, Kai Jiang, Gang He, Goran Ungar, Georg H. Mehl, Feng Liu

**Affiliations:** ^1^ Shaanxi International Research Center for Soft Matter, State Key Laboratory for Mechanical Behavior of Materials Xi'an Jiaotong University Xi'an 710049 P. R. China; ^2^ MOE Key Laboratory for Nonequilibrium Synthesis and Modulation of Condensed Matter, School of Physics Xi'an Jiaotong University Xi'an 710049 P. R. China; ^3^ Advanced Materials Research Group Faculty of Engineering University of Nottingham Nottingham NG7 2RD UK; ^4^ Institute of Chemical Sciences Heriot‐Watt University Edinburgh EH14 4AS UK; ^5^ Hunan Key Laboratory for Computation and Simulation in Science and Engineering Key Laboratory of Intelligent Computing and Information Processing of Ministry of Education School of Mathematics and Computational Science Xiangtan University Xiangtan Hunan 411105 P. R. China; ^6^ Frontier Institute for Science and Technology Xi'an Jiaotong University Xi'an 710049 P. R. China; ^7^ School of Chemical Materials and Biological Engineering University of Sheffield Sheffield S1 3JD UK; ^8^ Department of Chemistry University of Hull Hull HU6 7RX UK

**Keywords:** aggregate, columnar phase, liquid crystals, self‐assembly, squaraine dyes

## Abstract

For a series of novel hydroxy anilino squaraines (SQ), separated via short flexible spacers from bulky dendrons, hexagonal columnar (Col_hex_) liquid‐crystalline (LC) phase behavior is detected. The optical properties of these SQ systems, designed for melting below 80 °C, very low for SQ systems, are found to be determined by the LC structures which in turn are governed by volume and steric effects of the dendrons affecting the temperature‐dependent interplay between monomeric species and H‐aggregates in the LC state, ultimately responsible for the optical properties. Experimental results are corroborated by self‐consistent field theory (SCFT) as well as time‐dependent density functional theory calculations (TD‐DFT). The results demonstrate a new strategy for dynamically tuning aggregates and optical properties for the prescriptive functionalization of the LC SQ materials and with potential for other aggregating systems.

## Graphical abstract

A novel squaraine dye (SQ) forming columnar liquid crystal (LC) phase is synthesized, whose aggregate state can be tuned without disturbing the columnar structures. The liquid crystallization not only offers a rich spectrum and tuning method of optical property but also a novel path for the prescriptive functionalization of LC SQ materials.

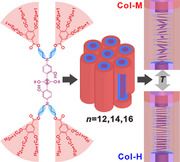

## 1 Introduction

Squaraine (SQ) based materials are attracting considerable attention due to a wide array of interesting optical properties with applications ranging from nonlinear optics,^[^ ^1^ ^]^ photovoltaics^[^ ^2^, ^3^ ^]^ to chemo‐/bio‐sensors.^[^ ^4^, ^5^, ^6^ ^]^ These technologies are associated with the high extinction coefficients of SQ materials and the tunability of absorption and fluorescence. These photophysical properties are linked to the zwitterionic character of the SQ chromophores, typically incorporated into a rigid conjugated donor–acceptor–donor (D‐A‐D) system with intramolecular charge transfer.^[^ ^7^ ^]^ For applications of SQ systems, the understanding of aggregation, such as H/J aggregation, their ensuing photophysical properties, and their correlation with chemical structure is essential.^[^ ^7^, ^8^, ^9^, ^10^, ^11^ ^]^ However, almost all SQ materials in bulk show untunable optical properties due to fixed aggregate structures, limiting so far their applications, especially as smart materials. Employing mesomorphic states has been demonstrated to be a feasible strategy in dynamically tuning aggregates by utilizing their fluid nature and developing functional materials,^[^ ^12^, ^13^ ^]^ such as the construction of 1D π‐stacks for charge carrier transport^[^ ^14^ ^]^ and the design of photoluminescent materials.^[^ ^15^ ^]^ However, due to the zwitterionic SQ units, SQ‐based liquid crystal (LC) materials tend to form very high melting materials.^[^ ^16^, ^17^ ^]^ This leads to issues of thermal stability for the organic systems or electronic devices, specifically when LC dyes are applied in highly energy‐efficient polarizer‐free information displays^[^ ^18^ ^]^ or adaptive lens systems.^[^ ^19^ ^]^

Here we report the preparation of a new class of LC SQ materials **SQ*n*** (*n* = 10–18), which exhibit hexagonal columnar phase formed below 80 °C, thus being in the thermally stable range of organic materials. To the best of our knowledge, this is the *first* observation of columnar phases in the SQ families. The 2D‐ordered columnar phase enables the modulation of SQ aggregation by the thermal environment without disturbing the mesophase. Based on detailed structure analysis using synchrotron XRD and thin film spectroscopy together with theoretical simulations ‐ time‐dependent density function theory (TD‐DFT) and self‐consistent field theory (SCFT), we show that the subtle interplay between molecular structure and global order determines the aggregation and photophysical properties.

## 2 Results and Discussion

### 2.1 Synthesis

In our design, the anilino SQ core is chosen for shape anisotropy and enhanced electronic interactions. Hydroxy groups are introduced in SQ chromophores to improve the yield and chemical stability.^[^ ^20^ ^]^ Moreover, intramolecular hydrogen‐bonding planarizes the squaraine π‐scaffold which facilitates the formation of well‐defined aggregates.^[^ ^21^ ^]^ To optimize the melting behavior and self‐assembly properties, two wedge‐shaped dendrons are attached at each end, based on gallate groups functionalized with long aliphatic chains (octyl (C8) to octadecyl (C18)).^[^ ^22^, ^23^ ^]^ Critical is the selection of a flexible propyl spacer between the SQ chromophores and the dendrons. These spacers with four bonds in length separate the central and peripheral aromatic groups, giving rise to flexibility, then addressing the critical issues of melting behavior and thermal stability.

**Scheme 1:**
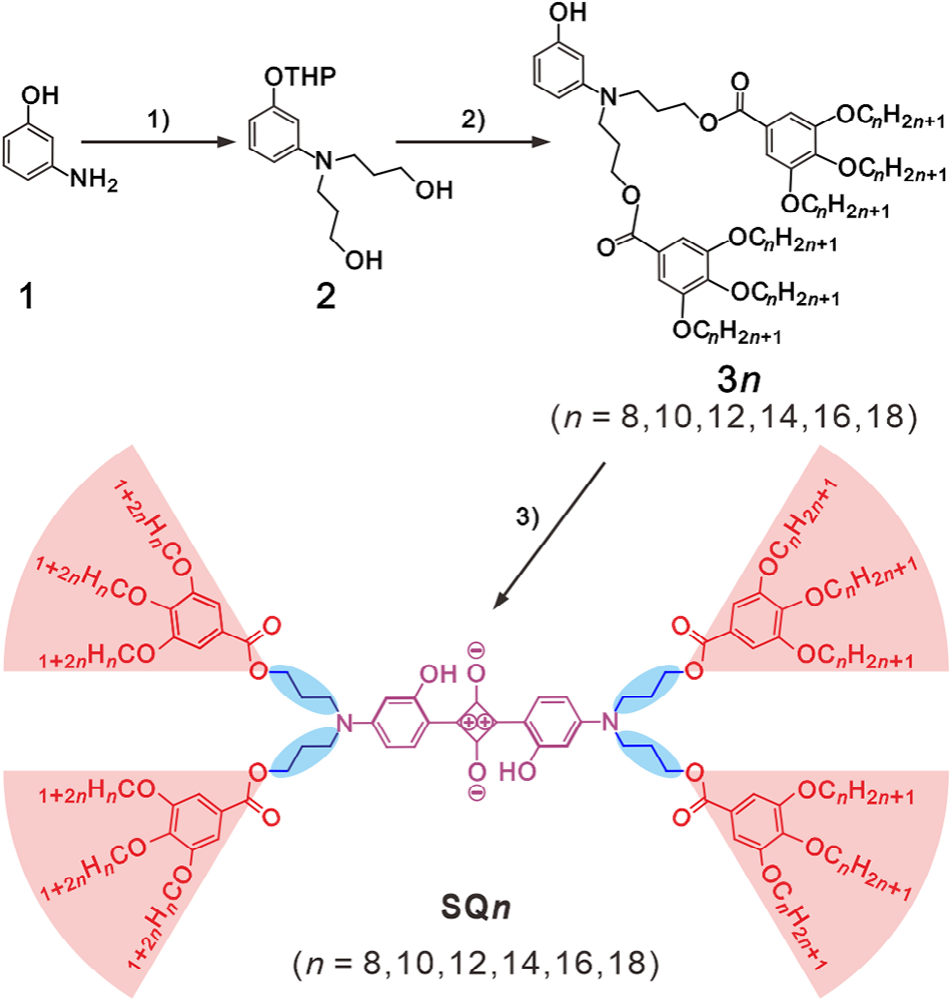
Synthesis of **SQ*n***: 1) a) NaBr, CH_3_COOH, MA, 95 °C; b) DHP, PPTS, DCM, r.t.; c) LiAlH_4_, THF, 0 °C to r.t.; 2) a) EDCI, DMAP, DCM, r.t.; b) Dowex (50WX8‐100‐200(H)), MeOH/THF, 80 °C; 3) squaric acid, *n*‐butanol/toluene, 140 °C.

This extendable synthetic strategy of constructing SQ materials is highly modular and efficient, as shown in **Scheme** **1**. The amino group of 3‐aminophenol (compound **1**) was alkylated in a reaction with methyl acrylate (MA) (yield: 95%), followed by protection of the phenol function as a tetrahydropyran (THP) ether (yield: 60%). A subsequent reduction with LiAlH_4_ yields compound **2** (yield: 90%) with terminal hydroxy functions. Esterification with alkyloxy‐substituted gallates was performed using EDCI/DMAP (yields: 53–70%), followed by a quantitative restoration of the phenol group using Dowex resin to afford compound **3*n***. The **SQ*n*** compounds were obtained as deeply colored solids by a condensation reaction of **3*n*** in *n*‐butanol/toluene at 140 °C with squaric acid in isolated yields of 33–50%. Details of the synthesis and the full chemical characterizations are provided in the Supporting Information (SI).

### 2.2 Liquid‐Crystalline Properties

**Figure 1:**
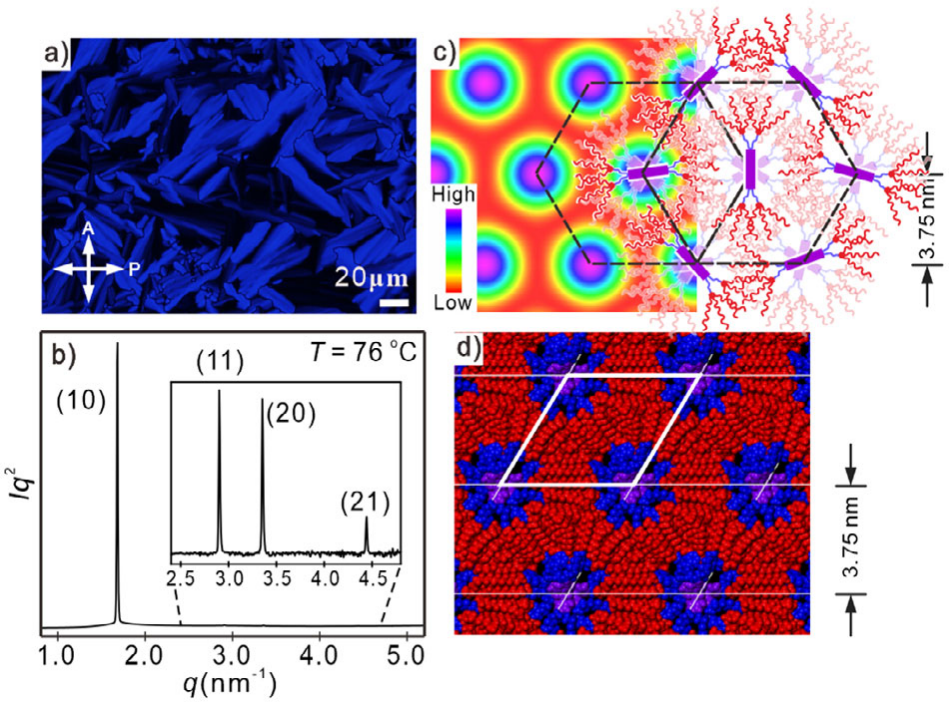
LC phase of compound **SQ12** at 76 °C a) Optical micrograph of Col_hex_ phase; b) SAXS diffractogram; c) Reconstructed ED map of the Col_hex_ phase with a schematic depiction of molecular arrangement; for easy visualization, some alkyl chains in (c) are omitted. The map refers to the electron density scale on the left. Color coding: purple/blue = SQ cores, green = spacers and gallate groups, yellow/red = alkyl chains. Note: SQ cores shown in (c) are schematic and are randomly rotated or as aggregates distributed along the columnar axis; d) Snapshot of a molecular dynamic simulation of Col_hex_ phase, details see Section S6.4 (Supporting Information).

The thermal behavior and LC properties were characterized by polarizing optical microscopy (POM), differential scanning calorimetry (DSC), and synchrotron small/wide angle X‐ray scattering (SAXS/WAXS) – for details see Supporting Information. Phase transitions and related lattice parameters are collated in **Table** **1** and Table S1 (Supporting Information). For compounds **SQ10** and **SQ18**, only crystal to isotropic (Iso) phase transitions were detected while LC phases were observed for **SQ12**, **SQ14,** and **SQ16**, with a monotropic LC phase for **SQ12** ranging from 80 to 72 °C and for **SQ16** in the temperature range of 73–61 °C upon cooling. **SQ14** forms an enantiotropic phase from 68 to 80 °C on heating and from 78 to 44 °C on cooling. Though the phase transition enthalpies of Iso‐LC for the above compounds are different, ranging from 3.0 J g^−1^ (9.5 kJ mol^−1^) for **SQ12** via 2.2 J g^−1^ (7.7 kJ mol^−1^) for **SQ14** to 1.5 J g^−1^ (5.7 kJ mol^−1^) for **SQ16**, the corresponding phase structures are similar. The birefringent optical textures observed between crossed polarizers confirm the LC state of **SQ12**, **SQ14,** and **SQ16**. The fan‐shaped textures suggest either a columnar phase or a highly ordered smectic variant, see **Figure** **1**a and Figures S13‐S15 (Supporting Information).

**Table 1:** Mesophase transitions and lattice parameters of **SQ*n***.

Compound	*n*	*T*/°C [ΔH J g^−1^]^a)^	Lattice parameters(*T*/°C)
**SQ10**	10	Iso 80 [8.5] Cr	/
**SQ12**	12	Iso 80 [3.0] Col_hex_/*p*6*mm* ^b)^ 72 [1.9] Cr	4.33 nm (76)
**SQ14**	14	Iso 78 [2.2] Col_hex_/*p*6*mm* ^c)^ 44 [13.9] Cr	4.51 nm (74)
**SQ16**	16	Iso 73 [1.4] Col_hex_/*p*6*mm* ^b)^ 61 [36.7] Cr	4.78 nm (68)
**SQ18**	18	Iso 67 [56.8] Cr	/

^a)^ Transition temperatures were determined using the onset temperature on cooling (rate: 10 K min^−1^). Abbreviations: Iso, isotropic liquid; Col_hex_, hexagonal columnar phase with *p*6*mm* symmetry; Cr, crystalline solid;

^b)^ monotropic liquid crystal;

^c)^ enantiotropic liquid crystal.

WAXS diffractograms show diffuse scattering with a maximum at 0.43–0.45 nm (see Figure S24, Supporting Information), typical for LC phases lacking fixed positions of individual molecules. Further detailed information on phase structure is obtained by performing X‐ray scattering experiments for **SQ12**, **SQ14,** and **SQ16** in the LC state. See Figure 1b as an example of the SAXS diffractogram of **SQ12** recorded at 76 °C, based on reflections indexed as (10), (11), (20), and (21), we derived a 2D hexagonal lattice (plane group *p*6*mm)* with a lattice parameter of *a*_hex_ = 4.33 nm (also see Table S3, Supporting Information). Only one small angle reflection could be recorded in compounds **SQ14** and **SQ16**, see Figure S17 (Supporting Information). The LC phase structures of these compounds were confirmed as a hexagonal phase by patterns of surface‐oriented thin films (Figures S18 and S19, Supporting Information), where (10) and (01) reflections are observed, azimuthally separated by 60°. Electron density (ED) maps for this structure were reconstructed, see Figure 1c and Section S5.1 (Supporting Information) for details. The data are consistent with the SQ chromophores with high electron density (blue/purple) stacked on top of each other, surrounded by a corona of hydrocarbon chains with lower electron density (yellow/red). The space‐filling of **SQ12** molecules in the Col_hex_ phase was evaluated too by molecular dynamic simulations using Materials Studio, see Figure 1d and Section S6.4 (Supporting Information). The lattice parameters *a*_hex_ of 4.33 nm at 76 °C for **SQ12**, 4.51 nm at 74 °C for **SQ14** and 4.78 nm at 68 °C for **SQ16**, are considerably smaller than the molecular lengths of 6.7, 7.2, and 7.7 nm respectively (Table S4, Supporting Information). This apparent discrepancy is typical for dendronized molecules in the Col_hex_ phase, mainly due to the coiling‐up of the alkoxy chains and to nearly vertical arrangement of molecules in neighboring columns^[^ ^24^, ^25^ ^]^see the model in Figure 1c. Based on the enumeration of states of a similar dendron (3,4,5‐tris‐(dodecyl) gallate) with self‐avoiding chains confined to a planar 90° sector on an atomistic diamond lattice and excluding gg* sequences, the number of possible conformations was determined as 41376644.^[^ ^26^ ^]^ For a molecule such as **SQ12**, there would thus be ≈10^29^ conformations, and this does not include the flexibility of the propylene spacer; only one of these conformations would have all twelve chains all‐*trans*. The dendron groups fill the surrounding volume of the column efficiently, and this is also reflected. in the associated radial volume distribution curves^[^ ^27^, ^28^ ^]^ and related calculations (see Section S6.3 Supporting Information).

**Figure 2:**
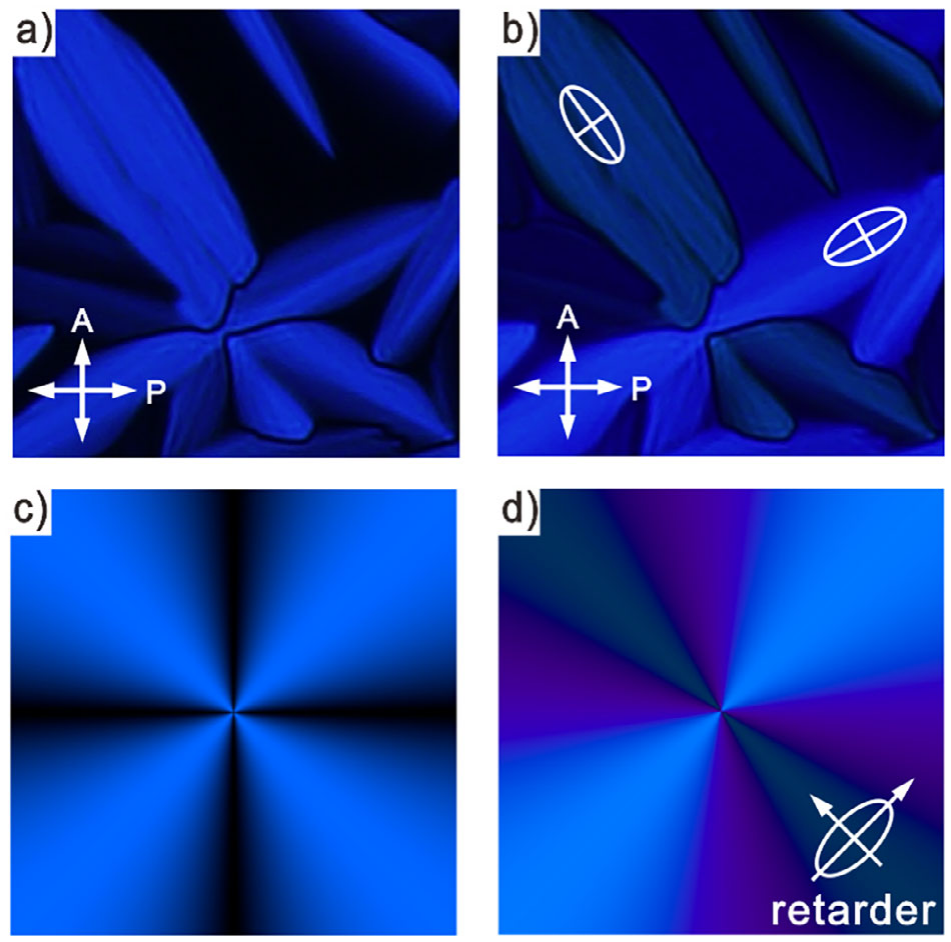
Optical textures between crossed polarizers of **SQ12** at 76 °C: a) Fan‐shaped texture and b) texture recorded with a retarder of Col_hex_ phase; c) Simulated optical texture of a developable domain (“spherulite”) of Col_hex_ phase with four “brushes” and a Maltese cross, and d) simulated texture of the same but with a retarder. The orientation of the retarder's indicatrix is shown.

Fan‐shaped optical textures using an additional retarder exhibit unusual colors due to the strong absorption of SQs, allowing virtually no transmission except some blue (see Figures 1a and **2**a,b). This hampers the determination of the optical indicatrix. Taking account of the absorption spectrum, a simulation of optical textures with a 550 nm *λ*‐plate (see Figure 2c,d) successfully reproduces these colors. This shows that the columns, which follow circular trajectories around the center of the Maltese cross, are optical negative and the molecules are positioned perpendicular to the column axis.

Employing the method of atomic volume increments in building an organic crystal unit cell,^[^ ^29^ ^]^ it is possible to deduce that 1 molecule occupies a columnar stratum of 4.4 Å high (Section S6.2 and Table S5, Supporting Information), and LC behavior is absent below a hydrocarbon volume fraction (V_a_) of 76% (for **SQ10**) and above 85% (for **SQ18**, Tables S1 and S4). As in almost all LCs, the simple shape of the rigid part (rod or disc) allows close packing and ensures low enthalpy H, whilst the flexible chains provide the entropy S. A stable enantiotropic LC phase forms if in a given temperature interval when *G* = *H* – *TS* is the lowest. Increasing alkyl chain length simultaneously increases the enthalpy and the entropy, which leads to a chain‐size window, in our case, C12–C16, allowing liquid crystal formation, see details in Section S10 (Supporting Information).

### 2.3 Photophysical Properties

**Figure 3:**
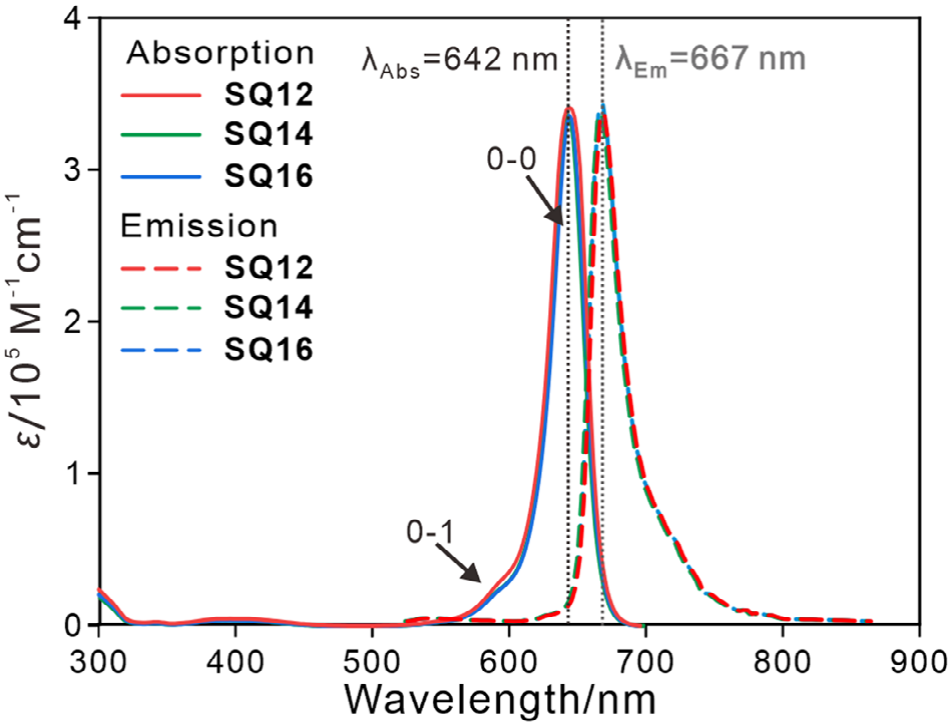
UV/vis/NIR absorption (solid lines) and emission (dotted lines) spectra of **SQ12**, **SQ14** and **SQ16** in DCM. (*c* = 10^−5^ M, *λ*_ex_ = 500–600 nm).

Diluted SQ*n* show high absorption coefficients *ε* of 3.1–3.3 × 10^5^ M^−1^ cm^−1^ in different solvents, and are characterized by distinct absorption (*λ*_abs_ = 642 nm) and emission (*λ*_em_ = 667 nm, λ_ex_ = 500–600 nm) maxima with Stokes shifts of 584 cm^−1^ in dichloromethane (**Figure** **3**), a value typical for hydroxy anilino SQs.^[^ ^7^ ^]^; A slight blue shift of the absorption maxima from 636 nm in methylcyclohexane and then a blue shift to 646 nm in tetrahydrofuran implied solvatochromism for **SQ*n***, for details of **SQ*n*** photophysical parameters see Section S7.1 (Supporting Information). Moreover, the absorption band (500–750 nm) comprises a dominant 0–0 peak (642 nm) and a weaker vibronic shoulder at ≈620 nm, which is typical for spectral signatures of anilino squaraines.^[^ ^21^ ^]^

**Figure 4:**
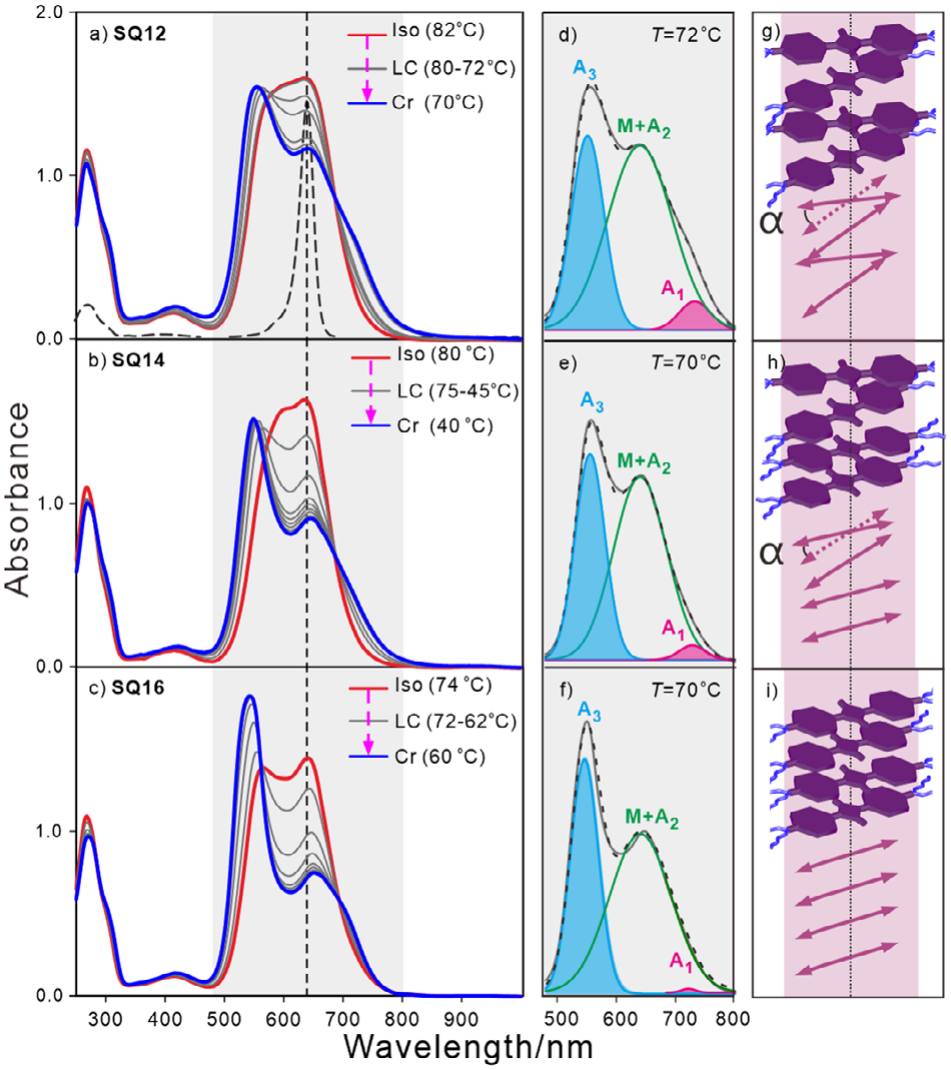
Temperature‐dependent UV/vis/NIR absorption spectra and local spectrum (grey) at the indicated temperature of a,d) **SQ12**; b,e) **SQ14**; c,f) **SQ16**. Three more realistic models of aggregates in Col_hex_ phase of g) **SQ12**; h) **SQ14**; i) **SQ16** are provided with hydroxy groups and peripheral chains omitted for easy visualization. Light purple arrows represent the transition dipole moments along the long axis of SQ cores, *α* indicates the rotation angle between neighboring SQ cores. Abbreviations: M = Monomers. The vertical dashed line in (a, b, c) marks the peak maximum for their respective monomers in DCM. The dashed curves in (d, e, f) show Gaussian fits for absorption profiles. Different absorption bands are colored differently for clarity. The dashed lines in (g, h, i) represent the column axis.

To correlate the optical properties with self‐assembled structure and aggregation in the LC state, temperature‐dependent absorption and photoluminescence (PL) spectra of **SQ*n*** thin films were recorded (**Figure** **4**; Section S7.1, Supporting Information). Their analysis is based on the study of exciton couplings in the LC state and phase transitions, such as H/J‐type aggregates characterized by slip angle *θ* between parallel aligned transition dipole moments, giving rise to blue‐shifted (*θ* > 54.7°) or red‐ shifted (*θ* < 54.7°) absorption bands when compared to the monomer.^[^ ^30^, ^31^, ^32^ ^]^ Molecular arrangements and related spectral properties were corroborated by TD‐DFT with the Gaussian 16 package^[^ ^33^ ^]^ using the CAM‐B3LYP functional^[^ ^34^ ^]^ and the 6–31+G* basis set (see Section S9, Supporting Information for details), based on SQ monomer and dimer. The dimer used as a model system for calculation is characterized by two geometrical parameters, including orientation angle α which describes the rotation and slip angle *θ*, based on which excitonic coupling dynamics of the aggregates were thoroughly studied.^[^ ^35^ ^]^

As can be seen in Figure 4, the UV/vis/NIR absorption spectra of **SQ12**, **SQ14**, and **SQ16** thin films are broadened, compared to that of a monomer in dichloromethane, dashed line in Figure 4a. This can generally be attributed to typical collisional broadening and more recently, this has been associated with phonon fluctuations.^[^ ^36^ ^]^ However, closer inspection of the spectra shows several additional features. In the isotropic state, monomeric species dominate, characterized by a narrower (red) peak compared to the LC and crystal states. In the LC state, a high‐energy band (A_3_ in Figure 4d–f) associated with H‐type coupling is intensified and blue‐shifted as the temperature decreases. For **SQ12** and **SQ14**, A_3_ band shifts from 570 to 550 nm, and from 560 to 540 nm for **SQ16**. Upon cooling, a quasi–isosbestic point is observed at ≈560 nm. Concurrently, the band at 640 nm, which is assigned to the monomeric species, as well as another band corresponding to H – aggregates^[^ ^37^, ^38^, ^39^ ^]^ (M and A2 in Figure 4), both exhibit a weakening trend. All these phenomena indicate the dynamic interchange between monomeric species and H – aggregates in the LC state. A lower intensity band at 720 nm that arises from slightly nonparallel arrangements of H‐aggregates, i.e., Davydov splitting (DS), see A_1_ band in Figure 4 intensifies with decreasing temperature. In the crystal state, a spectrum with a double‐hump structure (A_2_, A_3_) is observed, typical for hydroxy anilino SQs of monoclinic/orthorhombic polymorph,^[^ ^38^ ^]^ blue line in Figure 4a–c. Besides, a 400 nm band with low oscillator strength, unique for monohydroxy anilino SQs^[^ ^38^ ^]^ arises from a higher‐energy excitonic state according to our TD‐DFT calculation, see Table S9 (Supporting Information). In the temperature‐dependent PL spectra (Figure S36, Supporting Information), a weak, broad, and slightly structured emission band (≈900 nm) intensifies and shifts toward low energy on cooling. Considering the fluorescence suppression character of H‐aggregates in SQ systems, this emission band resulting in lower energy photons with large Stokes shifts (4200–4300 cm^−1^) can be attributed to strongly emissive optical monomers rather than aggregates,^[^ ^40^, ^41^, ^42^, ^43^, ^44^ ^]^ detailed discussion sees Section S7.1 (Supporting Information).

### 2.4 Structural Insights and Self‐Assembled Model

The correlation between spectral signatures and molecular arrangements in the Col_hex_ phase needs to be clarified. Nonparallel mutual orientation of transition dipole moments (described by orientation angle α in Figure 4), i.e., DS,^[^ ^41^, ^44^, ^45^, ^46^, ^47^ ^]^ causing lower and upper Davydov components, gives rise to the red‐shifted (A_1_) and the blue‐shifted (A_3_) band respectively, explaining the spectral signatures The red‐shifted A_3_ band at 720 nm has been attributed to short‐ranged Intermolecular charge transfer coupling (ICT) for some hydroxy anilino SQs, crystalizing in triclinic structure with only one molecule in each unit cell.^[^ ^21^, ^32^ ^]^ However, in our case the absence of face‐to‐face π‐π stacking reflection at a wide angle^[^ ^25^ ^]^ (Figure S24, Supporting Information) reflects poor face‐to‐face stacking of SQ chromophores, reducing the possibility of short‐range exciton coupling. Meanwhile, the crystal of **SQ14** exhibits the highest A_1_ band when compared to the isotropic/LC state, and that can be indexed as an orthorhombic phase according to GIWAXS patterns with more than 1 molecule in each unit cell, see Figure S20 (Supporting Information). Therefore, ICT coupling can be safely ruled out in our case

**Figure 5:**
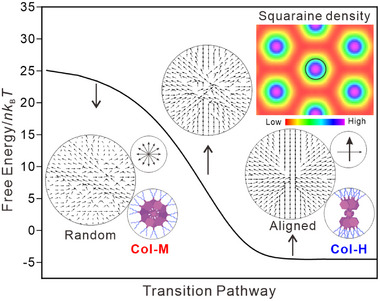
Squaraine orientations of each point (big black circle) inside the selected squaraine‐rich region and the averaged molecular orientation (small circle) as well as packing model (medium circle) of **SQ14**. The alignment of squaraines from Col‐M to Col‐H is an energy‐favored process. The inset shows the density map of the squaraine distribution. Two top‐views of more realistic aggregation models in Col_hex_ phase are displayed.

In the absence of specific strong interactions, the columnar phase and aggregate formation are closely related to nano‐segregation. To elucidate the aggregation behavior of squaraines and the related energy landscape, the SCFT^[^ ^48^, ^49^ ^]^ was applied to evaluate the alignment of squaraines (**Figure** **5**) by calculating the free energy dependence of squaraine distribution and orientation. These calculations reveal the probability of squaraine distribution (Figure 5, inset) and orientation at each point inside the selected area (Figure 5, big black circle), which corresponds to a stratum containing one molecule from an individual column. The averaged orientations (Figure 5, medium and small black circle) suggest the most possible situation from the perspective of system free energy. (see detailed discussion in Section S8, Supporting Information). The density map of the squaraine distribution (inset in Figure 5) in one columnar stratum is consistent with the columnar nanostructure and reconstructed ED maps obtained by SAXS in Figure 1. By tracking the orientations, the aggregation behavior can be partially supported. In the high energy state, there is no preferred squaraine orientation in each stratum, reflecting randomness in the column (Col‐M mode). In the low energy state, limited orientation in each stratum facilitates the H‐aggregation inside the column (Col‐H). Moreover, low energy states and similar phase transition pathways have been found in all **SQ*n*** series, either with random or parallel orientation as the initial state (Table S8 and Figure S45).

At higher temperatures in the LC state, weak H‐type coupling aggregates (A_3_ band at 560–570 nm) and monomers are dominant in the core region of the Col_hex_ phase, indicating most molecules randomly rotate at large angles *α* (*α* > 30°) along the columnar axis. Such a high‐temperature columnar phase leads to random orientated squaraines, which is in line with the high energy state in the SCFT model, see Col‐M in Figure 5. The large angle *α* is caused by the expansion of end groups along the column axis at high temperatures, which has been observed in the Col_hex_ phase for wedge‐shaped trialkoxybenzoate salts too,^[^ ^26^ ^]^ resulting in the low‐resolution monomer spectrum.^[^ ^44^ ^]^ As the temperature decreases, the A_3_ band shifts hypsochromically (550–540 nm) along with the weakening of the 640 nm band and the intensifying of the A_1_ band, indicating the transition from monomers to more well‐defined H‐aggregates with smaller *α* featuring more ideal parallel‐packing. Such packing is energy‐favored. The orientations of squaraine‐rich regions are gradually aligned at a lower free energy state, resulting in the columnar phase with one dominating molecular orientation. Such alignment supports the well‐defined H‐aggregates along columns, see Col‐H in Figure 5, Section S8 (Supporting Information) and Supporting Video S1. The origin of such alignment could be the contraction of dendrons on cooling, attenuating the clash of end groups along the columnar axis. The alignment of squaraines also leads to their denser packing, which is related to the small expansion of *a*_hex_ (0.1–0.2 nm) as well as the similar intermolecular distance of **SQ12**‐**16**, see Figures S24–S27 (Supporting Information). The denser packing is also supported by the squaraines density variation simulated from SCFT, see Supporting Video S2. The absence of J‐aggregates can also be explained by the need of if SQ systems to pack into columns with a well‐defined diameter.

Additionally, detailed TD‐DFT calculations based on dimers with different angle α further confirm the peak shift and the redistribution of the oscillator strength toward higher energies that arise from the decreasing angle *α*. The calculated A_3_ band (Table S10, Supporting Information) appears at ca 60° and continuously intensifies upon decreasing α from 60° to 0°, showing a blue shift of ≈10 nm, compared to 20 nm for experimental data. These results explain qualitatively the formation of well‐defined H‐aggregates and monomer–aggregate transitions in the Col_hex_ phase that arise from decreasing angle α. Meanwhile, the calculated A_2_ band for dimers with α ranging from 90° to 0° exhibits a small shift toward the monomer band, see Table S10 (Supporting Information), explaining the experimentally detected broadband at 640 nm, assigned to monomers and the A_2_ band of H‐aggregates at high temperatures in the LC state. Furthermore, in a small α region (0–20°), the A_1_ band exhibits both small oscillator strength and a large redshift (Table S10, Supporting Information), in line with the experimental results at a low temperature.

The relatively narrow and more pronounced blue‐shifted H‐type peaks of **SQ16** in comparison with those of **SQ12** at similar temperatures indicate that considerably more H‐aggregates are formed with more ideal parallel arrangement at low temperatures, see Figure 4d–i. This is also evidenced by a weaker A_1_ band in the spectra of **SQ16**, arising from a slight nonparallel orientation of H‐aggregates. For SQs with small lateral groups, such as short linear/branched alkyl chains (butyl (C4) ∼ (octyl (C8)), the electrostatic interactions of SQ chromophores and dispersion force of peripheral chains are comparable, steering the various aggregation behaviors (H/J) in crystals.^[^ ^38^, ^39^, ^42^, ^47^ ^]^ However, in our case the large branched dendron groups give rise to a large dispersion force overwhelming the electrostatic interaction between SQ cores, favoring H‐type aggregation. Furthermore, the better H‐type aggregation at low LC temperatures can be rationalized by elongation and parallel alignment of the hydrocarbon chains, guiding the enhanced H‐aggregation of the SQ cores. Recalling *G* = *H – TS*, the loss of the entropy penalty with decreased temperature (*ΔT)* and decreased conformational entropy (*ΔS)* of the chains can be compensated by the enthalpy gain of more ordered molecular stacking, i.e., better H‐aggregation. Likewise, long alkyl chains (hexadecyl, (C16)) as for **SQ16** tend to crystallize; this view is confirmed by **SQ18** which only forms crystals, forcing the SQ cores to form more well‐defined H‐aggregates.

**Figure 6:**
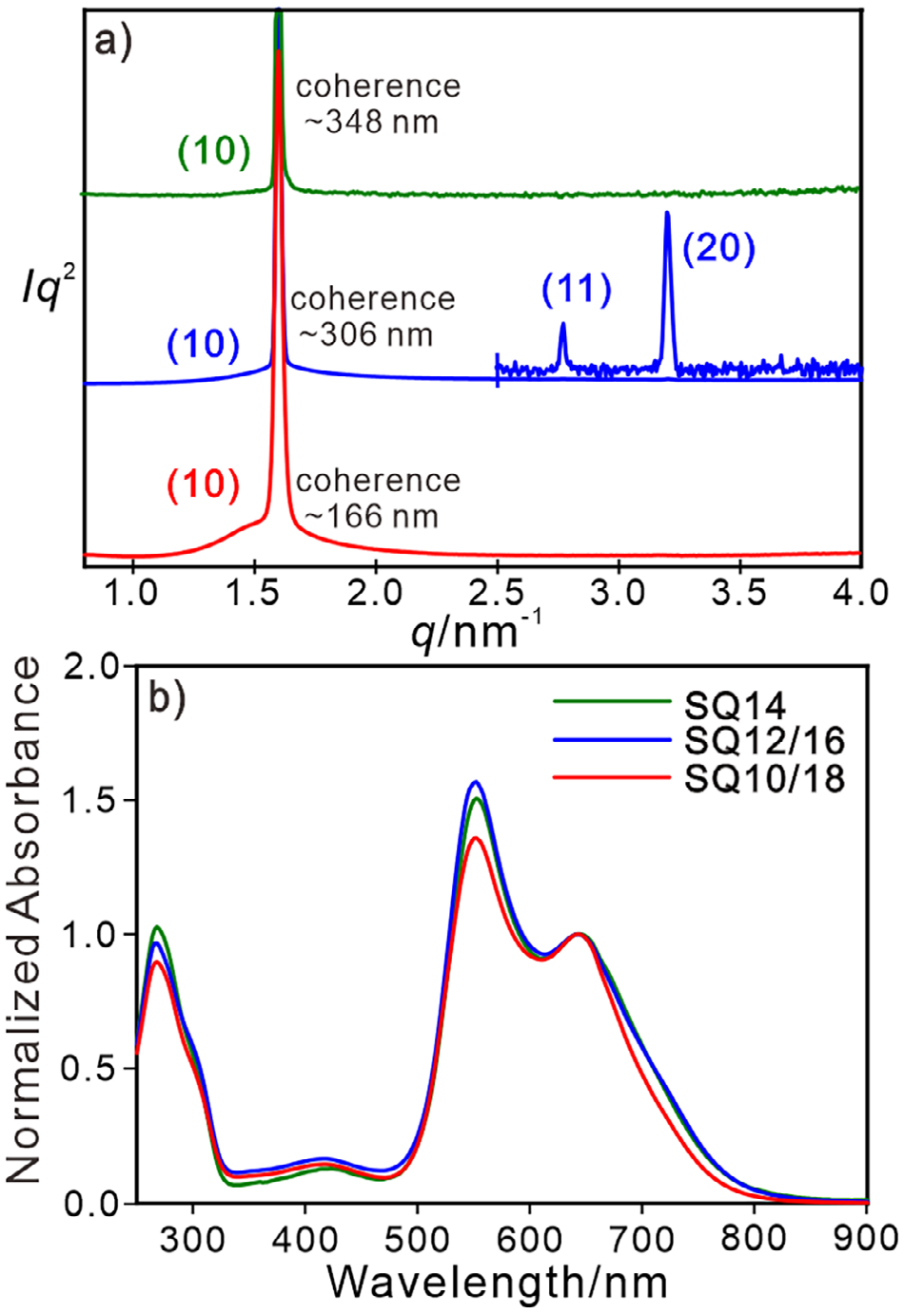
a) Transmission powder SAXS curves and b) normalized temperature‐dependent UV/vis/NIR absorption spectra of **SQ14**, **SQ10/18**, **SQ12/16** at 60 °C. Note: the absorption curves are normalized by the absorbance of 640 nm.

To further explore the effect of peripheral chains on the aggregation of SQ chromophores and confirm our analysis, five mixtures were prepared by mixing two different **SQ*n*** compounds in a 1:1 molar ratio, labeled **SQ10/14**, **SQ8/16**, **SQ12/16**, **SQ10/18** and **SQ14/18**. The overall aliphatic chain volume of **SQ10/14** and **SQ8/16** is identical to that of **SQ12**; **SQ12/16**, **SQ10/18** are similar to **SQ14** and **SQ14/18** to **SQ16**. The mixtures were systematically studied; for detailed information see Sections S3,S5, and S7, (Supporting Information). The mixtures form a Col_hex_ phase with *a*_hex_ very close to the pure compounds with an equivalent chain volume, see Figures S21–S27 (Supporting Information), showing that phase is closely related to the periphery volume. **SQ14** (**SQ12/16**, **SQ10/18**) exhibit an enantiotropic Col_hex_ phase, and are discussed here for their appropriate chain volume inducing thermal stable LC phase behavior. The lattice parameters *a*_hex_ of these samples are almost the same 4.63–4.64 nm at 60 °C, see Figure S26 (Supporting Information). Comparing **SQ14** and **SQ12/16**, thermal behaviors are similar as indicated by identical LC temperature ranges, transition enthalpies, and diffraction patterns (see **Figure** **6**a; Figure S38 and Table S2, Supporting Information). Besides that, the absorption curves are close (Figure 6b), implying not only similar phase structures but also aggregation behaviors in columns. For **SQ10/18** as well as for **SQ8/16** with a large chain‐length mismatch between the mixed components less ordered phase structures are formed with poor aggregation, identified through weaker H‐type spectral signatures, narrower LC temperature range, and smaller coherence lengths, see Figure 6, Figure S38 and Table S2 (Supporting Information). Therefore, the LC phase and aggregation behavior not only depends on the overall peripheral chain volume but also the judicious selection of chain lengths. This is further supported by mixtures with **SQ*n*** compounds with improper chain lengths, such as **SQ18** with overly long alkyl chains that tend to crystallize, while **SQ8** and **SQ10** with too short peripheral chains that lack flexibility to form LC phases. Aggregation is disturbed and LC phase stability is reduced, details see for details see the SI.

Overall peripheral chain volume with proper chain length guides the aggregation of SQ cores in the Col_hex_ phase. This can be explained by the predominant dispersion force and entropy of peripheral chains that steer the packing of SQ cores. Furthermore, large dendron groups hinder the formation of densely packed aggregates responsible for short‐ranged ICT coupling, just as for anilino SQs with branched alkyl chains^[^ ^38^, ^47^ ^]^ favoring Kasha‐type aggregation behavior and the nonideal parallel arrangement of H‐aggregates in the Col_hex_ phase. The dendritic soft alkyl chains also act as dual modulators in dynamically tuning aggregates on cooling without disturbing the global order of the columnar phase. Their volume controls the columnar nanostructure and the temperature sensitivity modulates the aggregation. Besides, We note that by simply shearing **SQ*n*** samples in the LC state we could prepare macroscale anisotropic thin films conveniently with columns and H‐aggregates orientating along the shearing direction, showing a dichroic ratio of 1.7 and a significant thermochromic character due to a sharp change of absorption, for details see Section S7.3 (Supporting Information). Therefore, **SQ*n*** LC materials with anisotropic absorption and temperature response characteristics have potential applications in sensing and display.^[^ ^19^ ^]^

## 3 Conclusion

**Figure 7:**
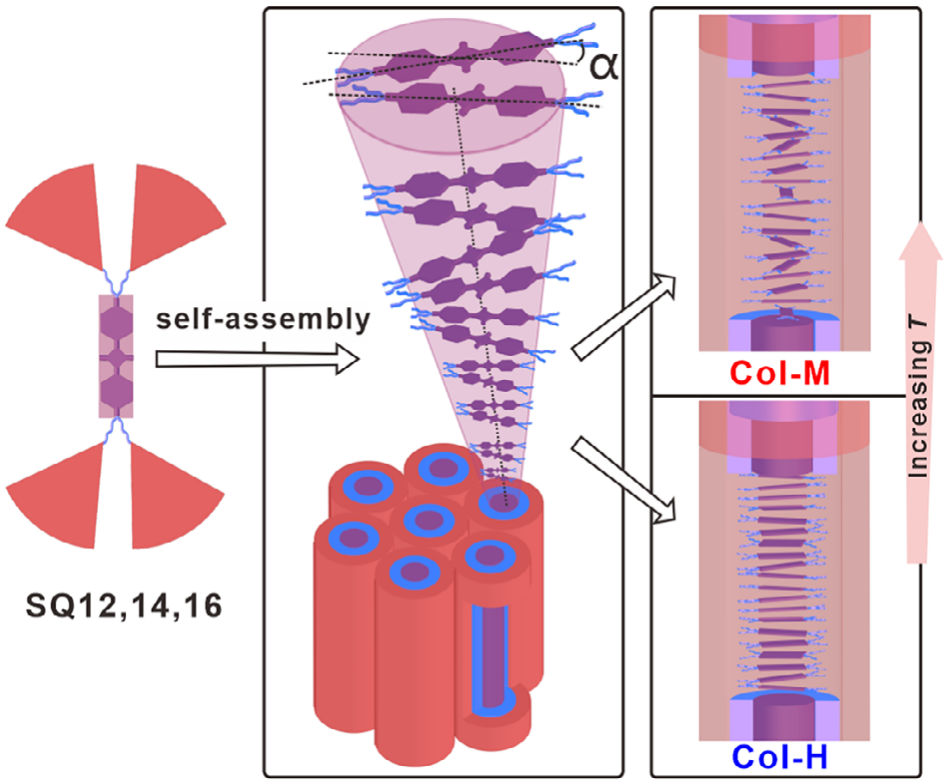
Stylized models of Col_hex_ phase of **SQ12**, **SQ14,** and **SQ16**. Col‐M/Col‐H represent the different aggregation behaviors in Col_hex_ phase at high/low temperatures, respectively. Cores, spacers, and dendrons are simplified as different shapes and are colored differently for clarity.

In conclusion, the first series of squaraine dyes forming columnar LC phases is reported with dual modes of aggregation, see **Figure** **7**. The materials feature a rigid zwitterionic central core linked to four wedge‐shaped dendritic terminal groups via short flexible spacers, ensuring melting below 80 °C. The volume requirements of the hydrocarbon dendrons, together with the electronic properties of the SQ chromophores determine the core–shelled Col_hex_ phase, which is responsible for the different aggregation behaviors of the SQ cores at different temperatures, i.e., monomers at high temperatures (Col‐M) and H‐type aggregates with slight non‐ideal parallel arrangement (Col‐H) at low temperatures. As the temperature decreases, a dynamic transition from monomers to H‐aggregates arises, evidenced by absorption spectra and calculations based on SCFT and TD‐DFT, modulating optical properties while maintaining the phase unchanged. The strategy takes advantage of dendritic soft alkyl chains, whose volume controls the columnar nanostructure and temperature sensitivity modulates the aggregation. Moreover, the macroscopic alignment of LC thin films, overcoming the disadvantages of polymorphs in crystals and precipitation in solvents found for SQ systems,^[^ ^21^, ^42^, ^47^ ^]^ provides an ideal platform to study different aggregates involving different excitonic couplings.

## 4 Experimental Section

### Materials

All chemicals and commercially available. Solvents were purchased and used without further purification unless otherwise stated. Squaric acid (99%), methyl acrylate (MA, 98%), LiAlH_4_ (99%) and Pyridinium *p*‐toluenesulfonate (PPTS, 98%) were purchased from Macklin; Dowex(50WX8‐100‐200(H)) resin was purchased from Alfa Aesar; 3,4‐Dihydro‐2H‐pyran (DHP, 98%) and 1‐(3‐Dimethylaminopropyl)‐3‐ethylcarbodiimide hydrochloride (EDCI, 98%) were purchased from Aladdin; 4‐dimethylaminopyridinium (DMAP, 98%) was purchased from Tokyo Chemical Industry, TCI.

### Chemical Characterizations

^1^H and ^13^C NMR spectra were recorded on a Bruker AV‐600 (^1^H: 600 MHz; ^13^C: 151 MHz) at 298 K. Chemical shift was reported relative to the residual solvent peak, coupling constants (*J*) are denoted in Hz, and chemical shifts (*δ*) in ppm. High‐resolution mass spectra (HRMS) were obtained on a time‐of‐flight mass spectrometer (WATERS I‐Class VION IMS QTof). Elemental analysis was conducted on Elementar vario EL cube/vario OXY cube element analyzer.

### Optical Spectroscopy—Polarizing Optical Microscopy (POM)

Thin film samples were prepared between glass plates and heated with a Linkam heating cell. The optical textures were recorded by Olympus BX51 using a CMOS image sensor.

### Optical Spectroscopy—UV/vis/NIR Spectroscopy

Absorption spectra were recorded by a Shimadzu UV‐3600 Plus spectrophotometer. The spectra of liquid samples were measured in quartz glass cuvettes by using spectroscopic grade solvents. Extinction coefficients were calculated based on Lambert‐Beer's law. The temperature‐dependent absorption spectra of thin films between quartz slices were measured by a Specac heating jacket. Polarized UV/vis measurements were performed with a fixed linear polarizer.

### Optical Spectroscopy—Fluorescence Spectroscopy

The fluorescence experiments were conducted on a fluorescence spectrophotometer (Edinburgh FLS1000). For solutions, standard quartz glass cuvettes were used. For temperature‐dependent fluorescence spectra of LC, thin films between quartz slices were measured. Excitation wavelength ranges from 500 to 600 nm.

### Thermal Behaviors Analysis—Differential Scanning Calorimetry (DSC)

Conventional differential scanning calorimetry (DSC) thermograms were recorded on a TA DSC250 calorimeter, purged with nitrogen. Heating and cooling rates were 10 K/min unless stated otherwise. Peak transition onset temperatures were quoted, and corrected for thermal lag.

### Structural Analysis—Small‐Angle X‐Ray Scattering (SAXS)

SAXS measurements were performed on Beamline BL16B1 at Shanghai Synchrotron Radiation Facility, SSRF. Experiments were carried out on solid samples in 1 mm glass capillaries under the control of a modified Linkam hot stage with thermal stability within 0.2 °C. The Pilatus 2M detector was applied in the experiments. *q* calibration and linearization were calibrated by silver behenate.

### Structural Analysis—Wide‐Angle X‐Ray Scattering (WAXS)

WAXS experiments were carried on SAXSpoint 2.0 of Anton Paar under vacuum with the powder samples in capillaries at the same temperature as the SAXS.

### Structural Analysis—Reconstructed Electron Density (ED) Maps

ED maps can be reconstructed with intensities *I*(*hk*) and phases *ϕ_hk_*. The general formula is as follows:

(1) $$E\left(xy\right)=\sum hksqrt\left[I\left(hk\right)]\;exp[i2\pi \left(hx+ky\right)+\theta_{hk}\right]$$

The ED maps were selected by a trial‐and‐error approach, where possible electron density maps are reconstructed for all possible phase combinations, details see Section S5.1 (Supporting Information).

### Simulations—Molecular Dynamic Simulation

Utilizing the software (*Materials Studio, Accelrys*), the length of molecular segments was measured from molecular models after geometrical optimization with the Forcite module. The space‐filling of the hexagonal phase was simulated by molecular dynamics with the Forcite module. See detailed information in Section S6.4 (Supporting Information).

Simulation of colours for fan‐shaped textures: The fan‐shaped textures were simulated according to the directors of molecules in LC phase which can be described by Laplace equation. The spectrum of light source was simulated by Planck's black body radiation. The absorption of sample is estimated according to the experimental UV/vis/NIR absorption spectra. Taking birefringence Δ*n*, sample thickness *d* and the retarder (λ = 550 nm) into consideration, the spectrum of emergent light can be simulated and translated into sRGB by color matching function.

### Simulations—Self‐Consistent Field Theory (SCFT)

SQ*n* molecules were considered as binary thirteen‐block polymers‐12 flexible blocks (alkyl chains) and 1 rigid block (SQ cores). SCFT model was applied to calculate the nano‐segregation of Col_hex_ phase. Five independent model parameters were used in the calculation–volume fraction of rigid block (*f*_R_), two interaction parameters ( χ_AR_, η), one parameter β measuring the conformational asymmetry and one parameter λ describing the hardness of rod molecule. By constructing a model and resolving the free energy, the SQ density and orientation in the Col_hex_ phase can be derived. Details and related parameters are in Section S8 (Supporting Information).

### Simulations—Time‐Dependent Density Functional Theory (TD‐DFT)

The molecular structures of monomer and dimer for DFT calculations were simplified by terminating the N atom with two methyl groups. The ground‐state geometries of SQ monomer and dimer were optimized with the Coulomb‐attenuated B3LYP (CAM‐B3LYP) exchange‐correlation functional together with Grimme's D3 van der Waals correction (CAM‐B3LYP+D3). All other calculations involving excited states were based on TD‐DFT. All DFT calculations were performed using the Gaussian 16 package and 6–31+G* basis set. Details and related parameters are in Section S9 (Supporting Information).

## Conflict of Interest

The authors declare no conflict of interest.

## Supporting information



Supporting Information

Supplemental Video 1

Supplemental Video 2

## Data Availability

The data that support the findings of this study are available in the supplementary material of this article.
