# Supplementary material for: Tuning Aggregation in Liquid‐Crystalline Squaraine Chromophores
Source: Adv Sci (Weinh). 2025 Mar 27;12(22):2416249. doi: 10.1002/advs.202416249 (PMC12165034; doi:10.1002/advs.202416249)
Supplement: Supplementary file 1 — Supporting Information [file ADVS-12-2416249-s002.docx]

**Supporting Infomation**

**Tuning Aggregation in Liquid-Crystalline Squaraine Chromophores**

Tianyi Tan^[a] +^, Yu Cao^[a,b] +^, Changlong Chen^[a]^, Sanliang Ling^[c]^, Gaolei Hou^[b]^, Martin J. Paterson^[d]^, Xin Wang^[e]^, Ying Chen^[e]^, Kai Jiang^[e]*^, Gang He^[f]*^, Goran Ungar^[a, g]*^, Georg H. Mehl^[a, h]*^ and Feng Liu^[a]*^

^[a]^ Shaanxi International Research Center for Soft Matter, State Key Laboratory for Mechanical Behaviour of Materials, Xi’an Jiaotong University, Xi’an 710049, P. R. China

^[b]^ MOE Key Laboratory for Nonequilibrium Synthesis and Modulation of Condensed Matter, School of Physics, Xi’an Jiaotong University, Xi’an 710049, P. R. China

^[c]^ Advanced Materials Research Group, Faculty of Engineering, University of Nottingham, Nottingham, NG7 2RD, U.K.

^[d]^ Institute of Chemical Sciences, Heriot-Watt University, Edinburgh, EH14 4AS, U.K.

^[e]^ School of Mathematics and Computational Science, Xiangtan University

^[f]^ Frontier Institute for Science and Technology, Xi’an Jiaotong University, Xi’an 710049, P. R. China

^[g]^ School of Chemical, Materials and Biological Engineering, University of Sheffield, Sheffield, S1 3JD, U.K.

^[h]^ Department of Chemistry, University of Hull, Hull HU6 7RX, U.K.

**Contents**

1. Materials and methods S4

2. Synthesis and characterization S5

2.1 Synthesis of compound B S5

2.2 Synthesis of compound C S6

2.3 Synthesis of compound D*n* S6

2.4 Synthesis of compound E*n* S9

2.5 Synthesis of compound SQ*n* S12

3. DSC traces, transition enthalpies of SQ*n* and their mixtures S24

4*.* Additional optical micrographs and simulations S28

4.1 Additional optical textures for SQ*n* S28

5. Additional X-ray data S30

5.1 Synchrotron X-ray diffraction and electron density reconstruction S30

5.2 SAXS/WAXS/GISAXS data S32

5.3 Additional electron density maps for SQ*n* mixtures S37

6. Calculation of geometrical parameters of molecule and Lattice parameters S38

6.1 Calculated molecular volume, volume fractions of molecular segments S38

6.2 Calculation of the average number of molecules in “unit cell” S39

6.3 Radial volume distribution curve S40

6.4 Molecular dynamic simulation of molecule arrangement in columnar phase S41

7. UV/vis absorption spectra and PL spectra S42

7.1 PL spectra of SQ*n* S42

7.2 Additional temperature-dependent absorption spectra S44

7.3 Measurement of the dichroic ratio of thin film S46

8. SCFT Model and Method S47

8.1 SCFT Model S47

8.2 Method S52

9. Excited-State Calculations S55

9.1 Computational details S55

9.2 Computational results S55

9.3 Discussion S59

10. Additional discussion on phase sequence S60

11. References S61

# 1. Materials and methods

All chemicals and commercially available solvents were purchased and used without further purification unless otherwise stated. Reactions were performed under an atmosphere of Argon. Reactions were monitoed by TLC plate (precoated with silica gel, F-254) and visualized by UV light with wavelength of 254 nm and 365 nm. Columns were packed with silica gel (300 - 400 mesh) as the stationary phase. ^1^H and ^13^C NMR spectra were recorded on a Brüker AV-600 (^1^H: 600 MHz; ^13^C: 151 MHz) at 298 K. Chemical shift was reported relative to residual solvent peak, coupling constants (*J*) are denoted in Hz and chemical shifts (*δ*) in ppm. Multiplicities are denoted as follows: br = broad, s = singlet, d = doublet, m = multiplet, t = triplet. High-resolution mass spectral (HRMS) data were obtained on a time-of-flight mass spectrometer(WATERS I-Class VION IMS QTof). Elemental analysis were conducted on Elementar vario EL cube/vario OXY cube element analyzer. The UV/vis experiments were conducted on a absorption spectrophotometer (UV-3600, Shimadzu). The spectra of liquid sample were measured in quartz glass cuvettes by using spectroscopic grade dichloromethane. Extinction coefficients were calculated base on Lambert-Beer’s law. Polarized UV/vis measurements were performed by using a fixed linear polarizer in the measurement. The fluorescence experiments were conducted on a Edinburgh FLS1000 fluorescence spectrometer.

# 2. Synthesis and characterization

**Scheme S1.**Synthesis of **SQ*n***. Reagents and conditions: (a) NaBr, CH_3_COOH, methyl acrylate (MA), 95 ℃; (b) DHP, PPTS, DCM, r.t.; (c) LiAlH_4_, THF, 0 ℃ to r.t.; (d) EDCI, DMAP, DCM, r.t.; (e) Dowex (50WX8-100-200(H)), MeOH/THF, 80 ℃; (f) squaric acid, *n*-butanol/toluene, 140 ℃.

Compound A^S1^(yield: 95%), compound **G*n***^S2^(***n*** =8, 10, 12, 14, 16, 18) (yield: 85-91%) were synthesized according to the literature procedures.

## 2.1 Synthesis of compound B

To a stirring solution of compound **A** (4.59 g, 16.32 mmol) and Pyridinium p-toluenesulfonate (0.41 g, 1.63 mmol) in DCM (60 ml) was added dropwise with 3,4-Dihydro-2H-pyran (3 ml, 32.69 mmol) in 30 minutes under the protection of Argon at 0 ℃, then the mixture was stirred at room temperature for 2 days. The mixture was washed with sat. NaHCO_3_ aq. solution (20 ml), the organic layer was separated, dried with MgSO_4_, and filtered. After removal of the solvent under reduced pressure, the residue was purified using silica gel column chromatography with acetate: petroleum (1/4, v/v, 1% Et_3_N) to afford compound **3** (3.58 g, 9.80 mmol, 60%) as a colorless viscous liquid. **^1^H NMR** (600 MHz, CDCl_3_) δ 7.09 (t, *J* = 8.2 Hz, 1H), 6.53 – 6.21 (m, 3H), 5.35 (t, *J* = 3.3 Hz, 1H), 3.94 – 3.82 (m, 1H), 3.66 – 3.52 (m, 11H), 2.61 – 2.53 (m, 4H), 2.02 – 1.91 (m, 1H), 1.85 – 1.77 (m, 2H), 1.68 – 1.52 (m, 3H). **^13^C NMR** (151 MHz, CDCl_3_) δ 172.44, 158.54, 147.98, 130.07, 106.23, 104.70, 101.33, 96.42, 62.12, 51.64, 46.93, 32.22, 30.46, 25.24, 19.00. **HRMS-ESI** m/z 366.1919 ([M+H]^+^[C_19_H_27_NO_6_H]^+^, calc. 366. 1911).

2.2 Synthesis of compound C

To a stirred suspension of LiAlH_4_ (0.74 g, 19.50 mmol) in anhydrous THF (30 ml), a solution of compound **B** (3.58 g, 9.80 mmol) in anhydrous THF (30 ml) was added dropwise at 0 ℃ under the protection of Argon. The reaction was stirred for 2 h at room temperature. The reaction mixture was quenched by water at 0 ℃, and filtered. The filtrate was dried with MgSO_4_, filtered and concentrated under reduced pressure. The crude product was purified by column chromatography with methanol: dichloromethane (1:50, v/v, 1% Et_3_N) as the eluent to yield compound **C** (2.73 g, 8.82 mmol, 90%) as yellow viscous liquid.**^1^H NMR** (600 MHz, CDCl_3_) δ 7.10 (t, *J* = 8.2 Hz, 1H), 6.52 – 6.32 (m, 3H), 5.40 (t, *J* = 3.3 Hz, 1H), 3.98 – 3.88 (m, 1H), 3.71 – 3.65 (m, 4H), 3.62 – 3.55 (m, 1H), 3.48 – 3.32 (m, 4H), 2.06 – 1.94 (m, 1H), 1.88 – 1.77 (m, 6H), 1.71 – 1.54 (m, 3H). **^13^C NMR** (151 MHz, CDCl_3_) δ 158.19, 149.39, 129.80, 106.54, 104.30, 101.26, 96.12, 62.08, 60.32, 48.04, 30.37, 29.97, 25.15, 18.78. **HRMS-ESI** m/z 310.2013 ([M+H]^+^[C_17_H_27_NO_4_H]^+^, calc. 310.2018).

2.3 Synthesis of compound D*n*

**1) D8**

The mixture of 3,4,5-tris(octyloxy)benzoic acid (0.5 g, 0.99 mmol), compound **C** (0.14 g, 0.45 mmol), EDCI (0.19 g, 0.99 mmol) and DMAP (55 mg, 0.45 mmol) were dissolved in anhydrous dichloromethane (20 ml). The mixture was stirred at room temperature for 16 h. Then the reaction mixture was added 50 ml dicromethane and washed with 20 mL water for three times, the organic phase was dried with MgSO_4_. After filtration, the solvent was removed under vacuum and the crude was purified by column chromatography with acetate : petroleum (1:40, v/v,1% Et_3_N) as the eluent to afford compound **D*n* (*n*** = 8**)** (0.35 g, 0.27 mmol, 60%) as a white solid. **^1^H NMR** (600 MHz, CDCl_3_) δ 7.27 (s, 4H), 7.08 (t, *J* = 8.1 Hz, 1H), 6.47 – 6.36 (m, 3H), 5.35 (s, 1H), 4.35 (t, *J* = 6.3 Hz, 4H), 4.14 – 3.94 (m, 12H), 3.90 (s, 1H), 3.56 (d, *J* = 11.3 Hz, 1H), 3.48 (t, *J* = 7.1 Hz, 4H), 2.17 – 2.01 (m, 4H), 2.00 – 1.87 (m, 1H), 1.86 – 1.69 (m, 14H), 1.68 – 1.53 (m, 3H), 1.53 – 1.40 (m, 12H), 1.41 – 1.14 (m, 48H), 0.89 (t, *J* = 6.9 Hz, 18H). **^13^C NMR** (151 MHz, CDCl_3_) δ 166.28, 158.46, 152.80, 148.94, 142.42, 129.86, 124.62, 107.88, 106.25, 104.21, 101.37, 96.37, 73.43, 69.11, 62.72, 62.04, 47.96, 31.84, 31.77, 30.36, 30.29, 29.46, 29.31, 29.28, 29.23, 26.74, 26.05, 26.00, 25.17, 22.64, 22.62, 18.86, 14.04. **HRMS-ESI** m/z 1304.0156 ([M+NH_4_]^+^[C_79_H_131_NO_12_NH_4_]^+^, calc. 1304.0010).

**2) D10**

Compound **D10** was synthesized according to the same procedure as for the synthesis of compound **D8** except using the different reagent: 3,4,5-tris(decyloxy)benzoic acid.**^1^H NMR** (600 MHz, CDCl_3_) δ 7.27 (s, 4H), 7.08 (t, *J* = 8.1 Hz, 1H), 6.43 (dd, *J* = 14.0, 6.2 Hz, 3H), 5.35 (t, *J* = 3.2 Hz, 1H), 4.36 (t, *J* = 6.3 Hz, 4H), 4.02 (dt, *J* = 13.0, 6.5 Hz, 12H), 3.95 – 3.84 (m, 1H), 3.56 (dt, *J* = 8.6, 3.7 Hz, 1H), 3.49 (t, *J* = 7.2 Hz, 4H), 2.14 – 2.03 (m, 4H), 1.98 – 1.89 (m, 1H), 1.86 – 1.71 (m, 14H), 1.66 – 1.53 (m, 3H), 1.53 – 1.44 (m, 12H), 1.42 – 1.19 (m, 72H), 0.89 (t, *J* = 7.0 Hz, 18H). **^13^C NMR** (151 MHz, CDCl_3_) δ 166.25, 158.45, 152.79, 148.92, 142.40, 129.85, 124.60, 107.86, 106.23, 104.21, 101.36, 96.34, 73.41, 69.08, 62.69, 62.00, 47.95, 31.88, 31.85, 30.34, 30.29, 29.67, 29.61, 29.58, 29.53, 29.36, 29.34, 29.30, 29.28, 26.73, 26.05, 26.01, 25.16, 22.64, 22.62, 18.84, 14.04. **HRMS-ESI** m/z 1472.1912 ([M+NH_4_]^+^[C_91_H_155_NO_12_NH_4_]^+^, calc. 1472.1888).

**3) D12**

Compound **D12** was synthesized according to the same procedure as for the synthesis of compound **D8** except using the different reagent: 3,4,5-tris(dodecyloxy)benzoic acid. Yield: 53%. **^1^H NMR** (600 MHz, CDCl_3_) δ 7.26 (s, 4H), 7.07 (t, *J* = 8.1 Hz, 1H), 6.46 – 6.35 (m, 3H), 5.35 (t, *J* = 3.1 Hz, 1H), 4.35 (t, *J* = 6.3 Hz, 4H), 4.01 (q, *J* = 6.7 Hz, 12H), 3.93 – 3.86 (m, 1H), 3.61 – 3.53 (m, 1H), 3.48 (t, *J* = 7.1 Hz, 4H), 2.06 (dd, *J* = 23.7, 17.1 Hz, 4H), 1.97 – 1.88 (m, 1H), 1.86 – 1.71 (m, 14H), 1.66 – 1.52 (m, 3H), 1.52 – 1.41 (m, 12H), 1.39 – 1.18 (m, 96H), 0.88 (t, *J* = 6.9 Hz, 18H). **^13^C NMR** (151 MHz, CDCl_3_) δ 166.32, 158.49, 152.83, 148.96, 142.44, 129.89, 124.63, 107.90, 106.29, 104.23, 101.40, 96.40, 73.47, 69.14, 62.75, 62.08, 47.99, 31.91, 30.38, 30.32, 29.73, 29.71, 29.69, 29.67, 29.64, 29.62, 29.56, 29.41, 29.37, 29.35, 29.31, 26.76, 26.09, 26.05, 25.19, 22.67, 18.89, 14.09. **HRMS-ESI** m/z 818.2168 ([M+Li] ^2+^[C_103_H_179_NO_12_ Li] ^2+^, calc. 818.2183).

**4) D14**

Compound **D14** was synthesized according to the same procedure as used for the synthesis of compound **D8** except using the different reagent: 3,4,5-tris(tetradecyloxy)benzoic acid. Yield: 70%. **^1^H NMR** (600 MHz, CDCl_3_) δ 7.25 (s, 4H), 7.11 – 7.03 (m, 1H), 6.44 (t, *J* = 13.4 Hz, 3H), 5.34 (s, 1H), 4.34 (t, *J* = 6.0 Hz, 4H), 4.01 (q, *J* = 6.6 Hz, 12H), 3.89 (t, *J* = 9.2 Hz, 1H), 3.55 (dd, *J* = 11.1, 4.0 Hz, 1H), 3.47 (s, 4H), 2.07 (s, 4H), 1.97 – 1.88 (m, 1H), 1.85 – 1.70 (m, 14H), 1.66 – 1.53 (m, 3H), 1.51 – 1.42 (m, 12H), 1.38 – 1.21 (m, 120H), 0.88 (t, *J* = 7.0 Hz, 18H). **^13^C NMR** (151 MHz, CDCl_3_) δ 166.33, 158.50, 152.84, 148.97, 142.46, 129.91, 124.64, 107.91, 106.30, 104.24, 101.41, 96.42, 73.48, 69.16, 62.76, 62.10, 48.00, 31.92, 30.39, 30.34, 29.74, 29.71, 29.68, 29.66, 29.64, 29.58, 29.42, 29.37, 29.33, 26.77, 26.11, 26.06, 25.20, 22.68, 18.89, 14.10. **HRMS-ESI** m/z 1791.5647 ([M+H]^+^[C_115_H_203_NO_12_H]^+^, calc. 1791.5378).

**5) D16**

Compound **D16** was synthesized according to the same procedure as used for the synthesis of compound **D8** except using the different reagent: 3,4,5-tris(hexadecyloxy)benzoic acid. Yield: 64%. **^1^H NMR** (600 MHz, CDCl_3_) δ 7.25 (s, 4H), 7.11 – 7.02 (m, 1H), 6.44 (t, *J* = 14.4 Hz, 3H), 5.34 (s, 1H), 4.34 (t, *J* = 6.0 Hz, 4H), 4.00 (q, *J* = 6.5 Hz, 12H), 3.94 – 3.85 (m, 1H), 3.59 – 3.53 (m, 1H), 3.47 (s, 4H), 2.07 (s, 4H), 1.98 – 1.86 (m, 1H), 1.85 – 1.70 (m, 14H), 1.66 – 1.52 (m, 3H), 1.50 – 1.43 (m, 12H), 1.38 – 1.18 (m, 144H), 0.88 (t, *J* = 7.0 Hz, 18H). **^13^C NMR** (151 MHz, CDCl_3_) δ 166.27, 158.47, 152.82, 148.94, 142.43, 129.87, 124.62, 107.88, 106.27, 104.25, 101.39, 96.36, 73.43, 69.11, 62.71, 62.03, 47.98, 31.90, 30.37, 30.32, 29.70, 29.65, 29.63, 29.56, 29.40, 29.35, 29.31, 26.76, 26.09, 26.05, 25.19, 22.66, 18.86, 14.07. **HRMS-ESI** m/z 986.3782 ([M+Li] ^2+^[C_127_H_227_NO_12_Li]^2+^, calc. 986.3746).

**6) D18**

Compound **D18** was synthesized according to the same procedure as used for the synthesis of compound **D8** except using the different reagent: 3,4,5-tris(octadecyloxy)benzoic acid. Yield: 65%. **^1^H NMR** (600 MHz, CDCl_3_) δ 7.27 (s, 4H), 7.08 (t, *J* = 8.1 Hz, 1H), 6.46 – 6.39 (m, 3H), 5.35 (t, *J* = 3.2 Hz, 1H), 4.35 (t, *J* = 6.3 Hz, 4H), 4.02 (dd, *J* = 14.4, 6.6 Hz, 12H), 3.94 – 3.86 (m, 1H), 3.56 (dt, *J* = 8.5, 3.6 Hz, 1H), 3.48 (t, *J* = 7.2 Hz, 4H), 2.12 – 2.04 (m, 4H), 1.99 – 1.89 (m, 1H), 1.86 – 1.71 (m, 14H), 1.69 – 1.52 (m, 3H), 1.52 – 1.44 (m, 12H), 1.40 – 1.19 (m, 168H), 0.89 (t, *J* = 7.0 Hz, 18H). **^13^C NMR** (151 MHz, CDCl_3_) δ 166.30, 158.48, 152.83, 148.96, 142.44, 129.89, 124.63, 107.89, 106.28, 104.25, 101.40, 96.38, 73.45, 69.13, 62.73, 62.05, 47.99, 31.92, 30.38, 30.33, 29.74, 29.71, 29.66, 29.65, 29.58, 29.42, 29.36, 29.32, 26.77, 26.11, 26.06, 25.20, 22.67, 18.88, 14.09. **HRMS-ESI** m/z1081.5749([M+NH_4_] ^2+^[C_139_H_251_NO_12_NH_4_]^2+^, calc. 1081.5869).

## 2.4 Synthesis of compound E*n*

**1) E8**

A mixed solution (methanol/tetrahydrofuran = 30ml/10ml) of Compound **D8** (0.35 g, 0.27 mmol) and Dowex (50WX8-100-200(H)) (0.1 g) was refluxed at 80℃ with vigorous stirring under the protection of Argon for 12 h. The reaction mixture was filtered at room temperature to remove the insoluble Dowex resin. The filtrate was concentrated under reduced pressure to afford compound **E8**(316mg, 0.26 mmol, 98%) as a white solid without further purification. **^1^H NMR** (600 MHz, CDCl_3_) δ 7.25 (s, 4H), 7.00 (t, *J* = 8.1 Hz, 1H), 6.44 – 6.07 (m, 3H), 5.31 (s, 1H), 4.33 (t, *J* = 6.0 Hz, 4H), 4.05 – 3.96 (m, 12H), 3.44 (t, *J* = 6.9 Hz, 4H), 2.08 – 2.02 (m, 4H), 1.83 – 1.69 (m, 12H), 1.49 – 1.43 (m, 12H), 1.35 – 1.23 (m, 48H), 0.87 (t, *J* = 6.6 Hz, 18H).**^13^C NMR** (151 MHz, CDCl_3_) δ 166.44, 152.79, 149.09, 142.38, 130.07, 124.49, 107.85, 104.72, 103.65, 99.54, 73.46, 69.08, 62.78, 47.85, 30.25, 29.66, 29.60, 29.58, 29.53, 29.51, 29.36, 29.33, 29.29, 29.26, 26.64, 26.04, 25.99, 22.61, 14.02. ^13^C NMR (151 MHz, CDCl_3_) δ 166.40, 157.11, 152.84, 149.17, 142.48, 130.15, 124.55, 107.93, 104.91, 103.53, 99.46, 73.50, 69.16, 62.75, 47.87, 31.86, 31.79, 31.46, 30.29, 30.08, 29.47, 29.33, 29.29, 29.25, 26.69, 26.06, 26.01, 22.65, 22.63, 14.06. **HRMS-ESI** m/z 1224.8896 ([M+Na]^+^[C_74_H_123_NO_11_Na]^+^, calc. 1224.8988).

**2) E10**

Compound **E10** was synthesized from compound **D10** using a similar procedure as for the synthesis of compound **E8**. **^1^H NMR** (600 MHz, CDCl_3_) δ 7.28 (s, 4H), 7.01 (t, *J* = 8.1 Hz, 1H), 6.36 – 6.10 (m, 3H), 4.34 (t, *J* = 6.2 Hz, 4H), 4.03 (dt, *J* = 20.6, 6.4 Hz, 12H), 3.45 (t, *J* = 7.0 Hz, 4H), 2.10 – 2.00 (m, 4H), 1.86 – 1.71 (m, 12H), 1.53 – 1.43 (m, 12H), 1.40 – 1.22 (m, 72H), 0.89 (t, *J* = 6.8 Hz, 18H). **^13^C NMR** (151 MHz, CDCl_3_) δ 166.44, 157.35, 152.79, 149.09, 142.38, 130.07, 124.49, 107.85, 104.72, 103.65, 99.54, 73.46, 69.08, 62.78, 47.85, 31.86, 31.85, 30.25, 29.66, 29.60, 29.58, 29.53, 29.51, 29.36, 29.33, 29.29, 29.26, 26.64, 26.04, 25.99, 22.61, 14.02. **HRMS-ESI** m/z 1388.1325 ([M+NH_4_]^+^[C_86_H_147_NO_11_NH_4_]^+^, calc. 1388.1312).

**3) E12**

Compound **E12** was synthesized from compound **D12** using a similar procedure as for the synthesis of compound **E8**. Yield: 97%. **^1^H NMR** (600 MHz, CDCl_3_) δ 7.27 (s, 4H), 7.06 – 6.97 (m, 1H), 6.36 – 6.10 (m, 3H), 4.34 (t, *J* = 6.2 Hz, 4H), 4.06 – 3.97 (m, 12H), 3.46 (t, *J* = 7.1 Hz, 4H), 2.12 – 2.00 (m, 4H), 1.87 – 1.70 (m, 12H), 1.51 – 1.43 (m, 12H), 1.40 – 1.18 (m, 96H), 0.88 (t, *J* = 7.0 Hz, 18H). **^13^C NMR** (151 MHz, CDCl_3_) δ 166.40, 157.11, 152.85, 149.17, 142.48, 130.16, 124.55, 107.92, 104.92, 103.54, 99.47, 73.50, 69.16, 62.75, 47.88, 31.90, 30.31, 29.72, 29.71, 29.69, 29.67, 29.64, 29.62, 29.56, 29.41, 29.37, 29.35, 29.31, 26.70, 26.09, 26.04, 22.66, 14.08. **HRMS-ESI** m/z 1561.2809 ([M+Na]^+^[C_98_H_171_NO_11_Na]^+^, calc. 1561.2744).

**4) E14**

Compound **E14** was synthesized from compound **D14** using a similar procedure as for the synthesis of compound **E8**. Yield: 98%. **^1^H NMR** (600 MHz, CDCl_3_) δ 7.27 (s, 4H), 7.02 (t, *J* = 8.1 Hz, 1H), 6.36 – 6.13 (m, 3H), 4.34 (t, *J* = 6.3 Hz, 4H), 4.02 (dt, *J* = 12.7, 6.5 Hz, 12H), 3.46 (t, *J* = 7.2 Hz, 4H), 2.11 – 2.01 (m, 4H), 1.79 (m, 12H), 1.47 (m, 12H), 1.41 – 1.16 (m, 120H), 0.89 (t, *J* = 7.0 Hz, 18H). **^13^C NMR** (151 MHz, CDCl_3_) δ 166.40, 157.11, 152.85, 149.18, 142.49, 130.17, 124.55, 107.92, 104.94, 103.54, 99.46, 73.50, 69.17, 62.75, 47.88, 31.91, 30.32, 29.73, 29.70, 29.66, 29.64, 29.57, 29.42, 29.36, 29.31, 26.70, 26.10, 26.05, 22.67, 14.09. **HRMS-ESI** m/z 1713.4843 ([M+Li]^+^[C_110_H_195_NO_11_Li]^+^, calc. 1713.4885).

**5) E16**

Compound **E16** was synthesized from compound **D16** using a similar procedure as for the synthesis of compound **E8**. Yield: 96%. **^1^H NMR** (600 MHz, CDCl_3_) δ 7.26 (s, 4H), 7.01 (t, *J* = 8.0 Hz, 1H), 6.39 – 6.11 (m, 3H), 4.34 (t, *J* = 5.9 Hz, 4H), 4.01 (q, *J* = 6.7 Hz, 12H), 3.46 (t, *J* = 6.9 Hz, 4H), 2.15 – 1.99 (m, 4H), 1.85-1.70 (m, 12H), 1.54 – 1.13 (m, 156H), 0.88 (t, *J* = 6.6 Hz, 18H). ^13^C NMR (151 MHz, CDCl_3_) δ 166.37, 156.96, 152.88, 149.23, 142.53, 130.21, 124.58, 107.95, 105.04, 103.47, 99.41, 73.52, 69.20, 62.72, 47.89, 31.92, 30.34, 29.75, 29.72, 29.67, 29.66, 29.59, 29.43, 29.37, 29.33, 26.73, 26.11, 26.07, 22.68, 14.11. **HRMS-ESI** m/z 944.3489 ([M+Li]^2+^[C_122_H_219_NO_11_Li]^2+^, calc. 944.3458).

**6) E18**

Compound **E18** was synthesized from compound **D18** using a similar procedure as for the synthesis of compound **E8**. Yield: 97%. **^1^H NMR** (600 MHz, CDCl_3_) δ 7.25 (s, 4H), 7.02 (t, *J* = 8.0 Hz, 1H), 6.38 – 6.11 (m, 3H), 4.34 (t, *J* = 6.2 Hz, 4H), 4.01 (q, *J* = 6.5 Hz, 12H), 3.46 (t, *J* = 7.0 Hz, 4H), 2.06 (s, 4H), 1.84 – 1.68 (m, 12H), 1.54 – 1.41 (m, 12H), 1.27 (m, 168H), 0.88 (t, *J* = 6.9 Hz, 18H). **^13^C NMR** (151 MHz, CDCl_3_) δ 166.35, 156.94, 152.88, 152.77, 149.20, 142.54, 130.24, 124.57, 107.97, 107.48, 105.09, 104.82, 103.50, 101.12, 99.43, 73.52, 69.21, 62.70, 47.92, 31.93, 30.34, 29.73, 29.66, 29.59, 29.44, 29.37, 29.34, 26.71, 26.12, 26.07, 22.69. **HRMS-ESI** m/z 1044.4099 ([M+Na]^2+^[C_134_H_243_NO_11_ Na]^2+^, calc. 1044.4135).

## 2.5 Synthesis of compound SQ*n*

**1) SQ8**

In a flask with Dean-Stark apparatus and a reflux condenser, the compound **E8** (316mg, 0.26 mmol) and squaric acid (15 mg, 0.13mmol) were suspended in a mixture of toluene (18 ml) and *n*-butanol (6 ml). The reaction was protected with Argon and stirred vigorously at 140 ℃. The color of the solution turned to yellow, then green and dark-blue in the end. After 9 h the reaction was cooled down to room temperature, the solvent was removed under reduced pressure on rotary evaporator. The residue was dissolved in dichloromethane (30 ml), then methanol (10 ml) was added to precipitate the deeply colored product. The precipitate was collected, and washed with methanol (15 ml) for three times, then the crude product was purified by column chromatography with dichloromethane: methanol (500 : 1 ~ 300 : 1, v/v) as the eluent to afford dark solids. After re-crystallization from dichloromethane/methanol compound **SQ8** (184 mg, 57%) was obtained as a dark-purple solid.**^1^H NMR** (600 MHz, CDCl_3_) δ 7.90 (d, *J* = 9.1 Hz, 2H), 7.25 (s, 8H), 6.41 (d, *J* = 9.2 Hz, 2H), 6.22 (s, 2H), 4.36 (t, *J* = 6.2 Hz, 8H), 4.02 (q, *J* = 6.4 Hz, 24H), 3.66 – 3.56 (m, 8H), 2.20 – 2.11 (m, 8H), 1.88 – 1.70 (m, 24H), 1.53 – 1.42 (m, 24H), 1.39 – 1.19 (m, 96H), 0.87 (q, *J* = 7.1 Hz, 36H).**^13^C NMR** (151 MHz, CDCl_3_) δ 182.59, 174.19, 166.29, 165.00, 156.23, 152.94, 142.71, 132.77, 124.15, 110.51, 107.92, 107.45, 98.93, 73.53, 69.23, 62.09, 48.51, 31.89, 31.83, 30.34, 29.51, 29.38, 29.36, 29.33, 29.29, 27.10, 26.11, 26.04, 22.68, 22.66, 14.10. **HRMS-ESI** m/z 2522.7819 ([M]+K^+^C_152_H_246_N_2_O_24_, calc. 2522.7722). **Elemental analysis** found: C 73.42%; H 10.02%; N 1.08%. C_152_H_246_N_2_O_24_. Required: C 73.45%; H 9.98%; N 1.13%.


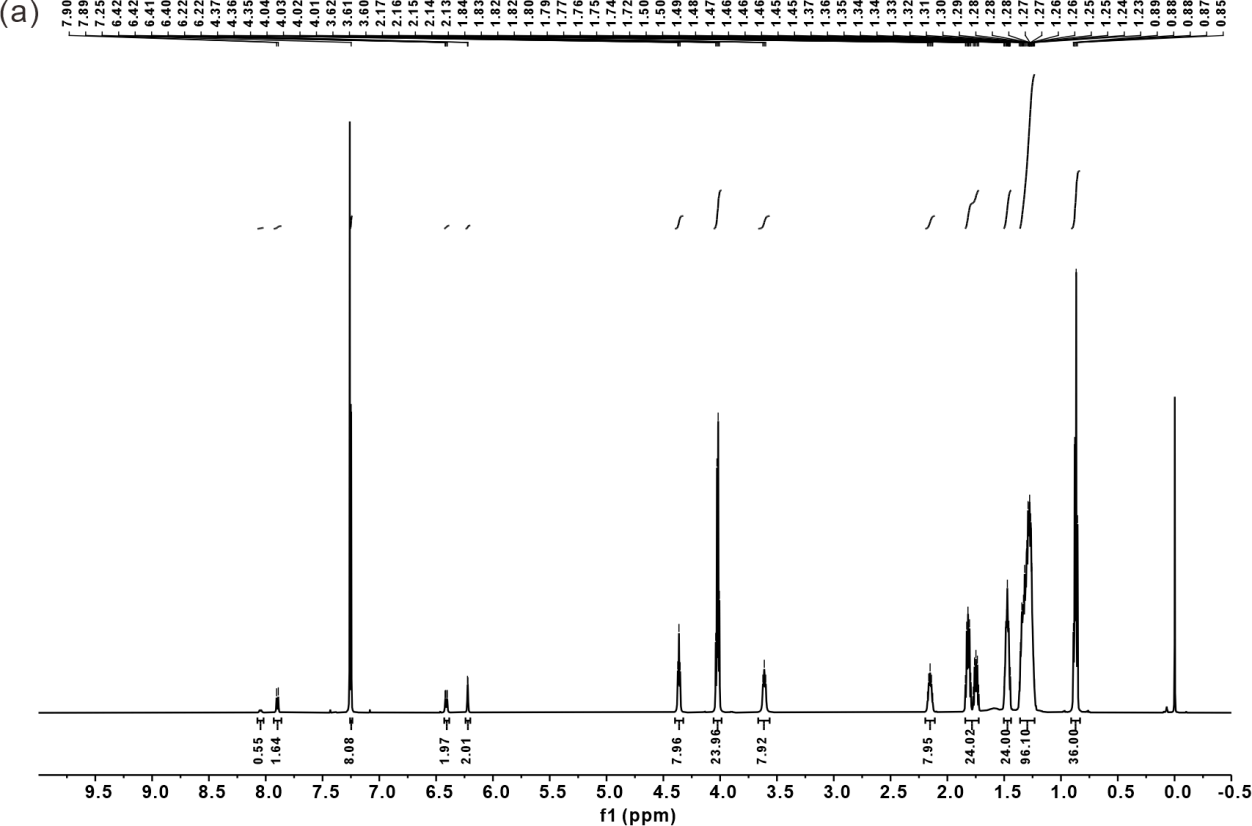


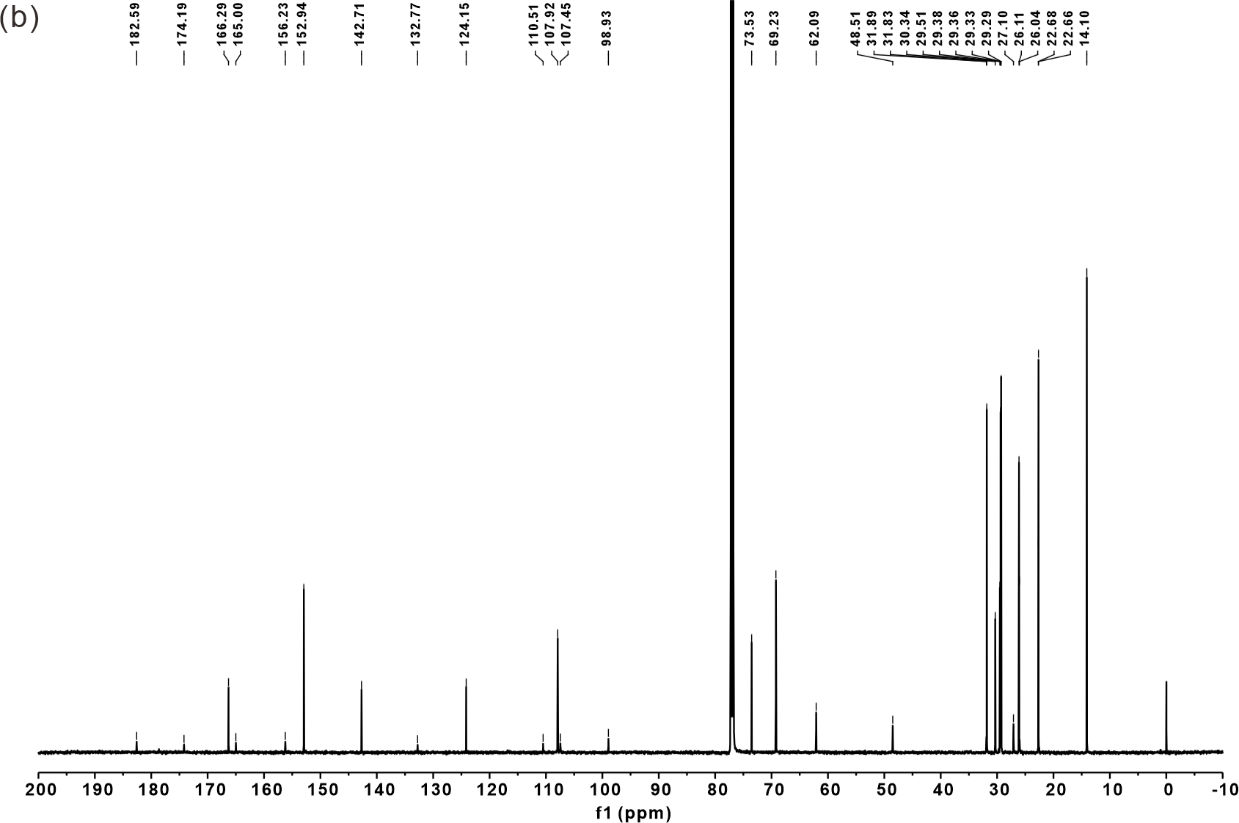


**Figure S1.** **SQ8**: (a) **^1^H NMR** spectrum (600 MHz, CDCl_3_, 298 K); (b) **^13^C NMR** spectrum (151 MHz, CDCl_3_, 298 K).

**2) SQ10**

**SQ10** was synthesized from compound **E10** using a similar procedure as for the synthesis of compound **SQ8**. **^1^H NMR** (600 MHz, CDCl_3_) δ 7.90 (d, *J* = 9.0 Hz, 2H), 7.25 (s, 8H), 6.41 (d, *J* = 9.0 Hz, 2H), 6.22 (s, 2H), 4.36 (t, *J* = 6.0 Hz, 8H), 4.02 (q, *J* = 6.4 Hz, 24H), 3.69 – 3.54 (m, 8H), 2.22 – 2.08 (m, 8H), 1.87 – 1.69 (m, 24H), 1.52 – 1.42 (m, 24H), 1.38-1.20 (m, 144H), 0.87 (q, *J* = 6.8 Hz, 36H).**^13^C NMR** (151 MHz, CDCl_3_) δ 182.57, 174.19, 166.27, 164.99, 156.21, 152.93, 142.70, 132.77, 124.13, 110.52, 107.90, 107.44, 98.93, 73.52, 69.21, 62.07, 48.50, 31.92, 31.90, 30.34, 29.72, 29.66, 29.64, 29.59, 29.57, 29.53, 29.49, 29.43, 29.38, 29.35, 29.32, 27.09, 26.11, 26.05, 22.68, 22.67, 14.09. **HRMS-ESI** m/z 2859.1573 ([M+K]^+^[C_176_H_294_N_2_O_24_K]^+^, calc. 2859. 1478). **Elemental analysis** found: C 74.34%; H 10.16%; N 0.95%. C_176_H_294_N_2_O_24_. Required: C 74.90%; H 10.50%; N 0.99%.


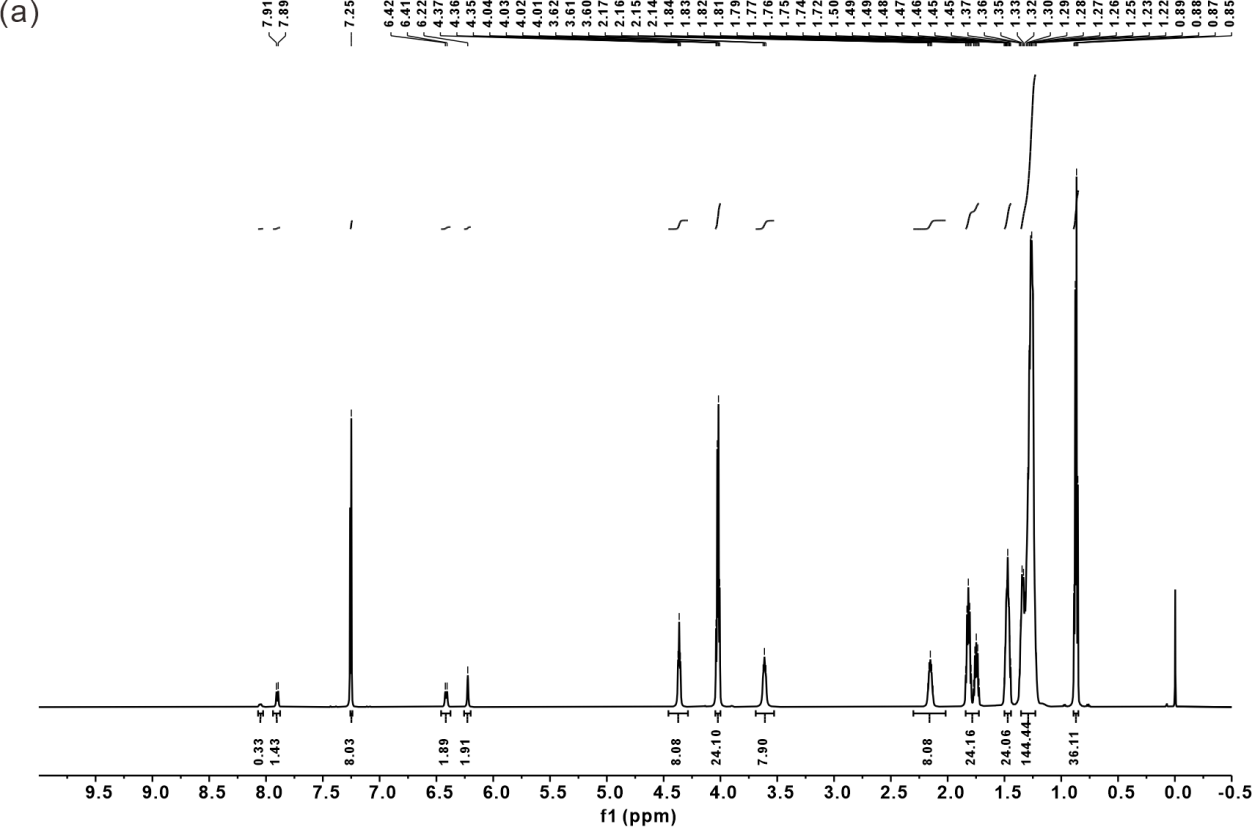


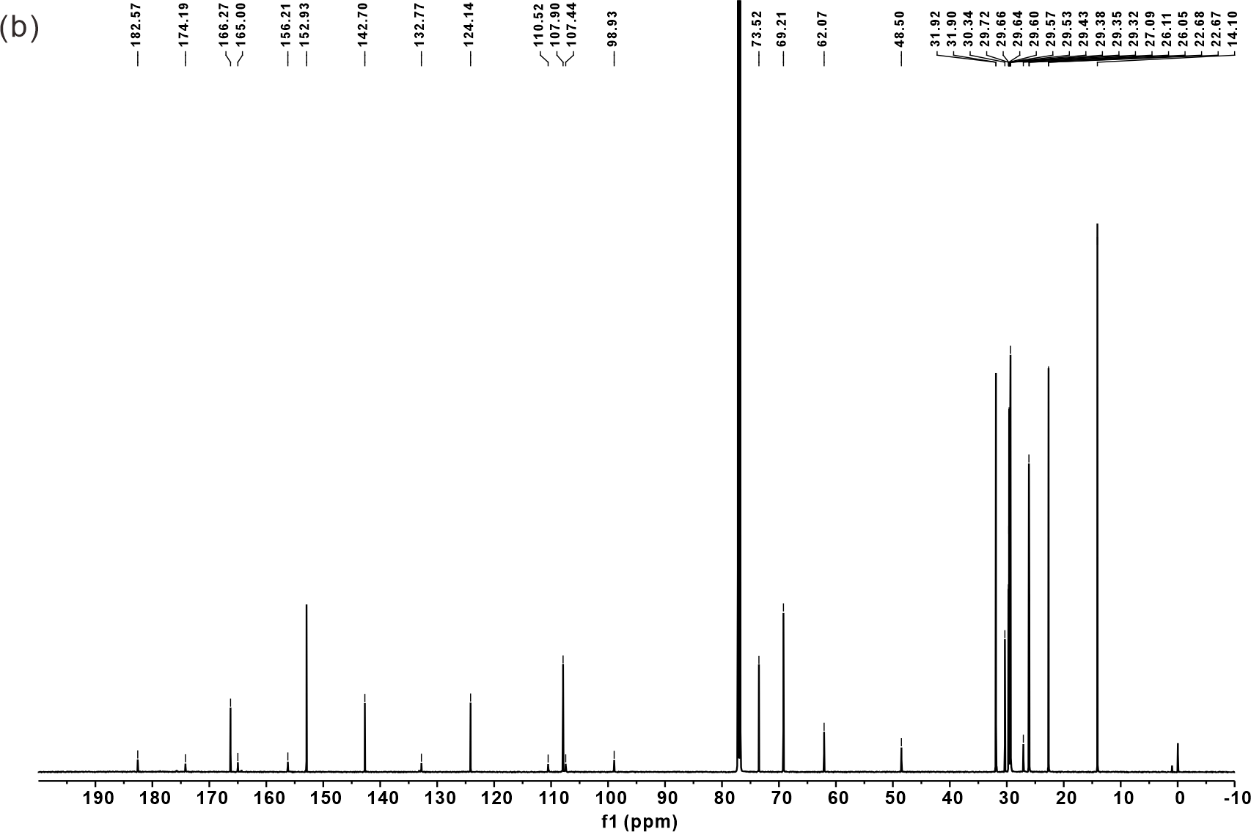


**Figure S2.** **SQ10**: (a) **^1^H NMR** spectrum (600 MHz, CDCl_3_, 298 K); (b) **^13^C NMR** spectrum (151 MHz, CDCl_3_, 298 K).

**3) SQ12**

**SQ12** was synthesized from compound **E12** using a similar procedure as for the synthesis of compound **SQ8** Yield: 45%. **^1^H NMR** (600 MHz, Chloroform-d) δ 7.90 (d, J = 9.1 Hz, 2H), 7.25 (s, 8H), 6.41 (d, J = 9.1 Hz, 2H), 6.22 (s, 2H), 4.36 (t, J = 6.1 Hz, 8H), 4.02 (q, J = 6.3 Hz, 24H), 3.71 – 3.49 (m, 8H), 2.22 – 2.09 (m, 8H), 1.84 – 1.72 (m, 24H), 1.50 – 1.44 (m, 24H), 1.35 – 1.22 (m, 192H), 0.87 (td, J = 7.0, 4.2 Hz, 36H).**^13^C NMR** (151 MHz, CDCl_3_) δ 182.58, 174.21, 166.28, 165.00, 156.23, 152.94, 142.70, 132.78, 124.14, 110.51, 107.91, 107.42, 98.91, 73.53, 69.22, 62.08, 48.49, 31.93, 31.92, 30.35, 29.75, 29.73, 29.71, 29.69, 29.67, 29.66, 29.59, 29.44, 29.39, 29.37, 29.33, 27.11, 26.13, 26.06, 22.68, 14.11. **HRMS-ESI** m/z 3157.5824 ([M+H]^+^[C_200_H_342_N_2_O_24_H]^+^, calc. 3157.5675). **Elemental analysis** found: C 76.07%; H 10.65%; N 0.84%; C_200_H_342_N_2_O_24_. Required: C 76.04%; H 10.91%; N 0.89%.


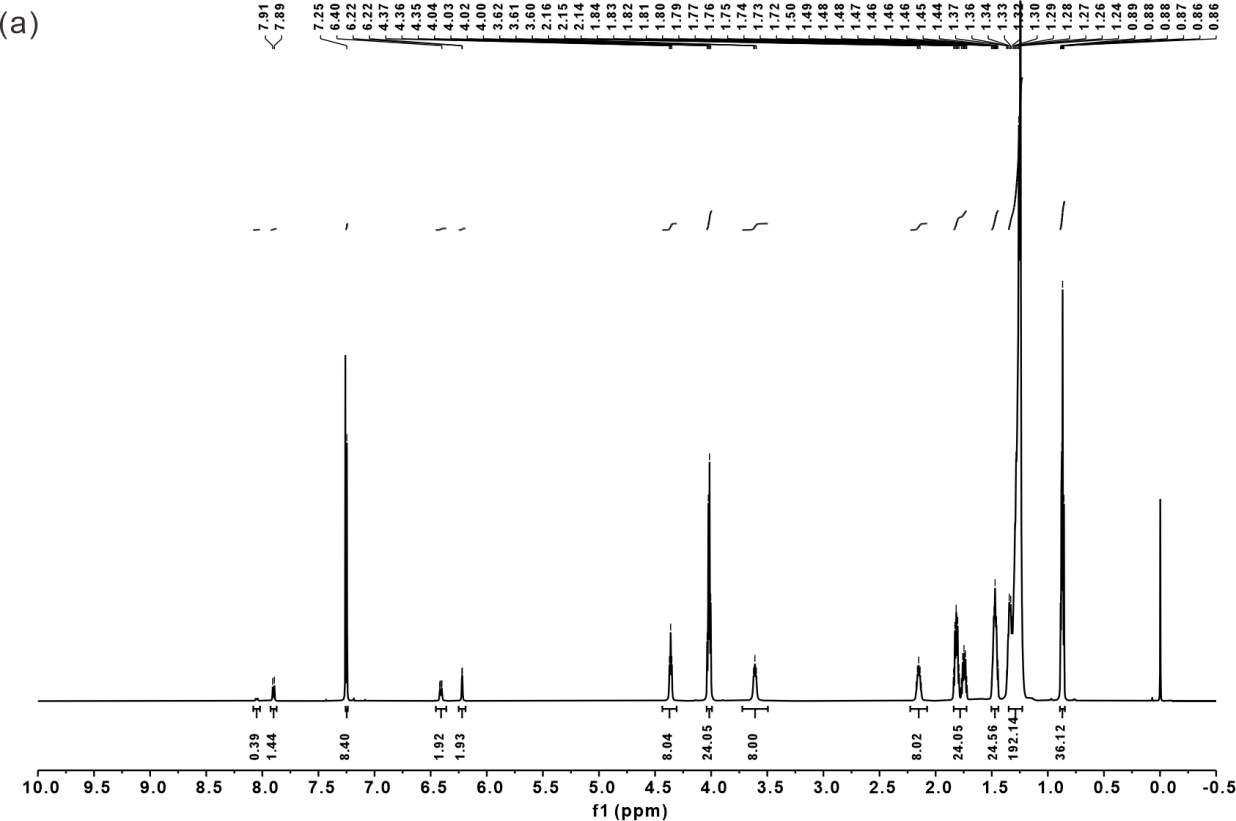


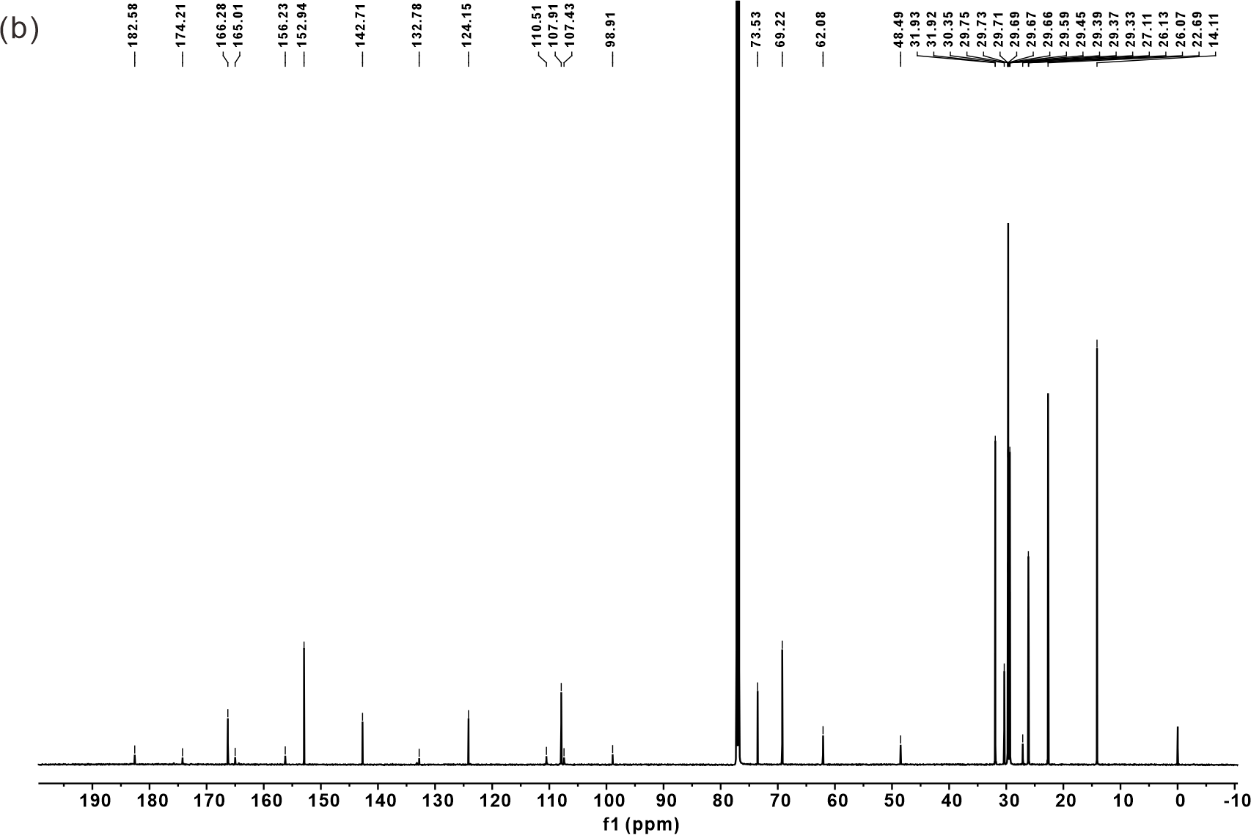


**Figure S3.** **SQ12**: (a) **^1^H NMR** spectrum (600 MHz, CDCl_3_, 298 K); (b) **^13^C NMR** spectrum (151 MHz, CDCl_3_, 298 K).

**4) SQ14**

**SQ14** was synthesized from compound **E14** using a similar procedure as for the synthesis of compound **SQ8**. Yield 41%. **^1^H NMR** (600 MHz, Chloroform-*d*) δ 7.90 (d, *J* = 9.1 Hz, 1H), 7.25 (s, 8H), 6.41 (dd, *J* = 9.0, 2.4 Hz, 2H), 6.22 (d, *J* = 2.3 Hz, 2H), 4.36 (t, *J* = 6.2 Hz, 8H), 4.02 (q, *J* = 6.3 Hz, 24H), 3.61 (t, *J* = 7.4 Hz, 8H), 2.15 (t, *J* = 7.2 Hz, 8H), 1.84 – 1.72 (m, 24H), 1.50 – 1.45 (m, 24H), 1.35 – 1.24 (m, 240H), 0.87 (td, *J* = 7.0, 2.8 Hz, 36H).**^13^C NMR** (151 MHz, CDCl_3_) δ 182.57, 174.21, 166.27, 165.00, 156.23, 152.94, 142.70, 132.78, 124.14, 110.49, 107.90, 107.41, 98.90, 73.52, 69.21, 62.07, 48.48, 31.92, 30.35, 29.76, 29.74, 29.72, 29.68, 29.66, 29.59, 29.50, 29.45, 29.37, 29.33, 27.11, 26.13, 26.06, 22.68, 14.10. **HRMS-ESI** m/z 3531.8740 ([M+K]^+^[C_224_H_390_N_2_O_24_K]^+^, calc. 3531. 8990). **Elemental analysis** found: C 76.90%; H 10.87%; N 0.74%. C_224_H_390_N_2_O_24_. Required: C 76.97%; H 11.25%; N 0.80%.


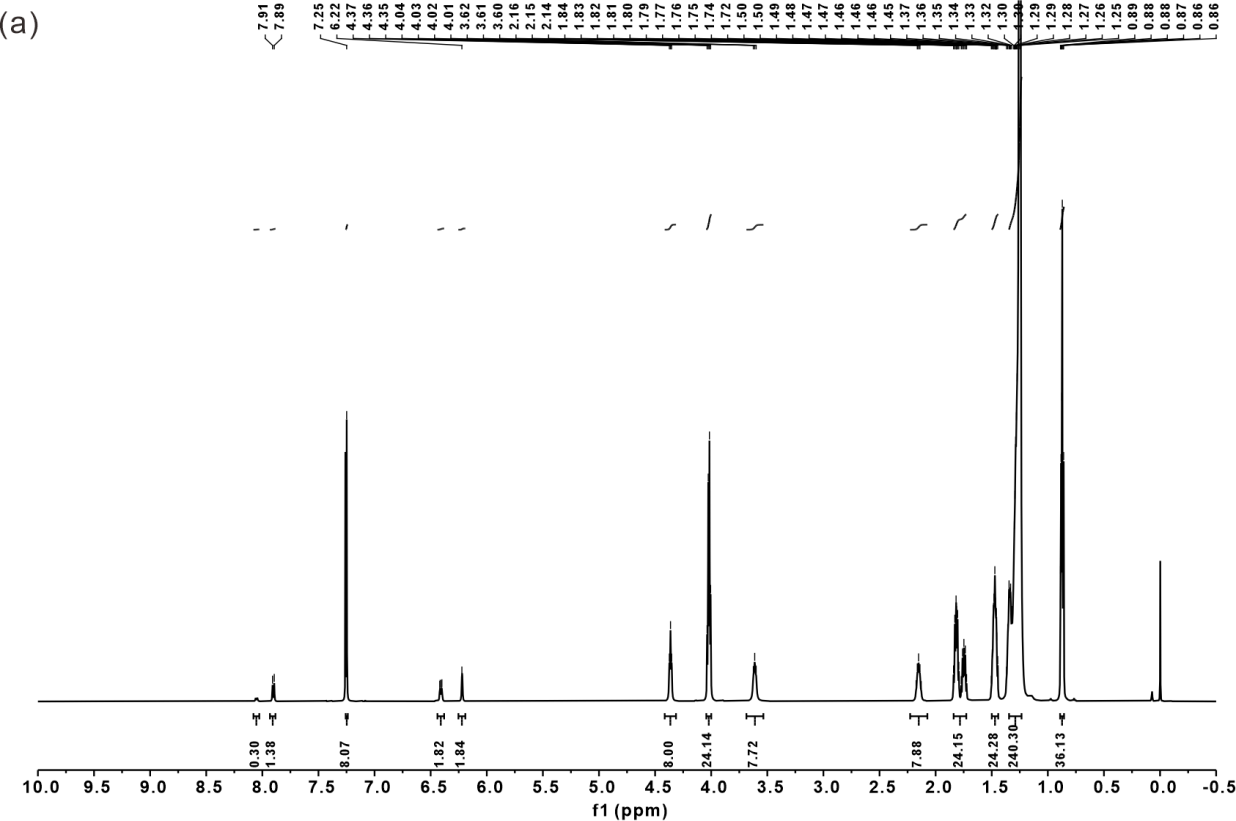


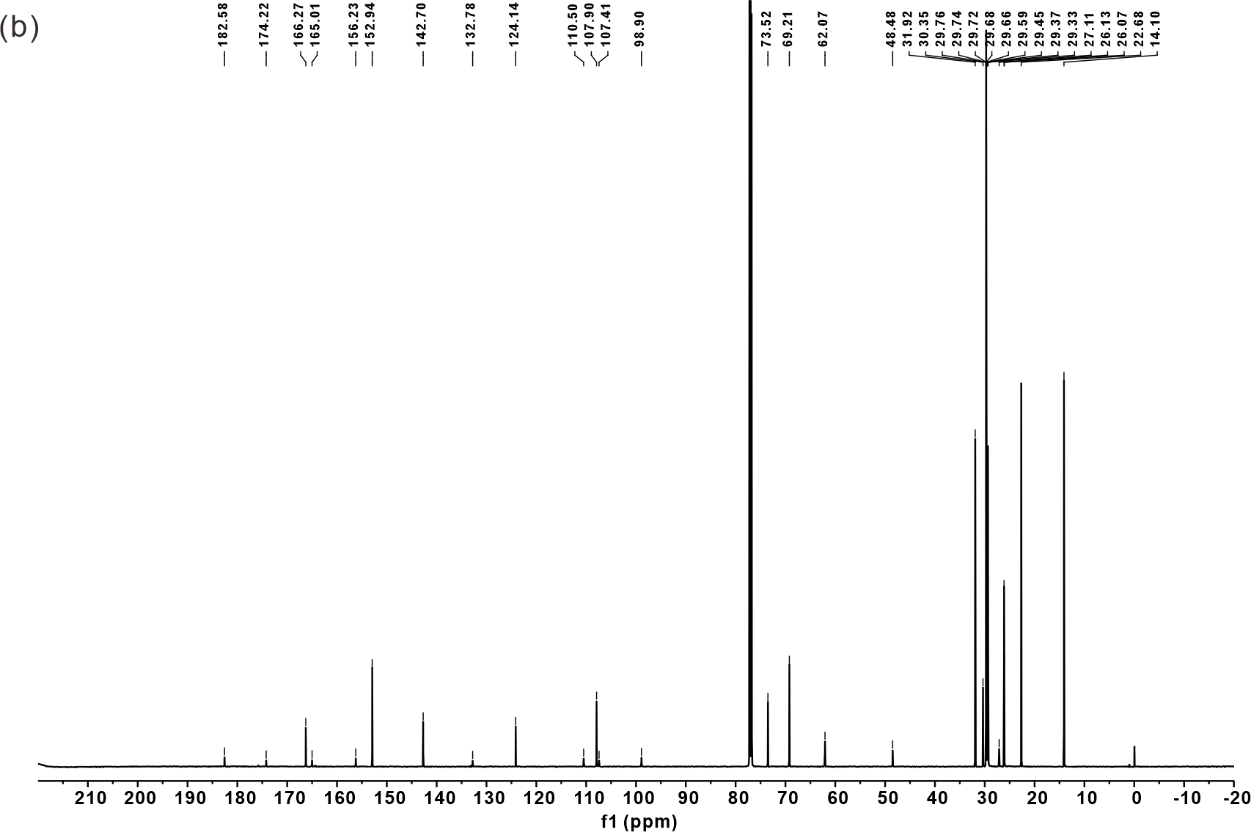


**Figure S4.** **SQ14**: (a) **^1^H NMR** spectrum (600 MHz, CDCl_3_, 298 K); (b) **^13^C NMR** spectrum (151 MHz, CDCl_3_, 298 K).

**5) SQ16**

**SQ16** was synthesized from compound **E16** using a similar procedure of synthesizing compound **SQ8**. Yield: 37%. **1H NMR** (600 MHz, Chloroform-d) δ 7.90 (d, J = 9.1 Hz, 2H), 7.25 (s, 8H), 6.41 (dd, J = 9.3, 2.6 Hz, 2H), 6.22 (d, J = 2.4 Hz, 2H), 4.36 (t, J = 6.2 Hz, 8H), 4.02 (q, J = 6.3 Hz, 24H), 3.61 (t, J = 7.4 Hz, 8H), 2.15 (t, J = 7.2 Hz, 8H), 1.84 – 1.72 (m, 24H), 1.47 (dq, J = 11.9, 7.6, 5.5 Hz, 24H), 1.35 – 1.23 (m, 288H), 0.87 (td, J = 7.0, 2.0 Hz, 36H). **^13^C NMR**(151 MHz, CDCl_3_) δ 182.58, 174.23, 166.27, 165.01, 156.25, 152.94, 152.88, 142.71, 132.78, 124.15, 110.47, 107.91, 107.39, 98.88, 73.52, 69.22, 62.07, 48.47, 32.03, 31.92, 30.36, 29.77, 29.76, 29.73, 29.67, 29.60, 29.50, 29.46, 29.44, 29.37, 29.34, 27.13, 26.14, 26.12, 26.07, 22.79, 22.68, 14.11. **HRMS-ESI** m/z 3868.2807 ([M+K]^+^[C_248_H_438_N_2_O_24_K]^+^, calc. 3868.2741). **Elemental analysis** found: C 77.74%; H 11.31%; N 0.70%. C_248_H_438_N_2_O_24_. Required: C 77.73%; H 11.52%; N 0.73%.


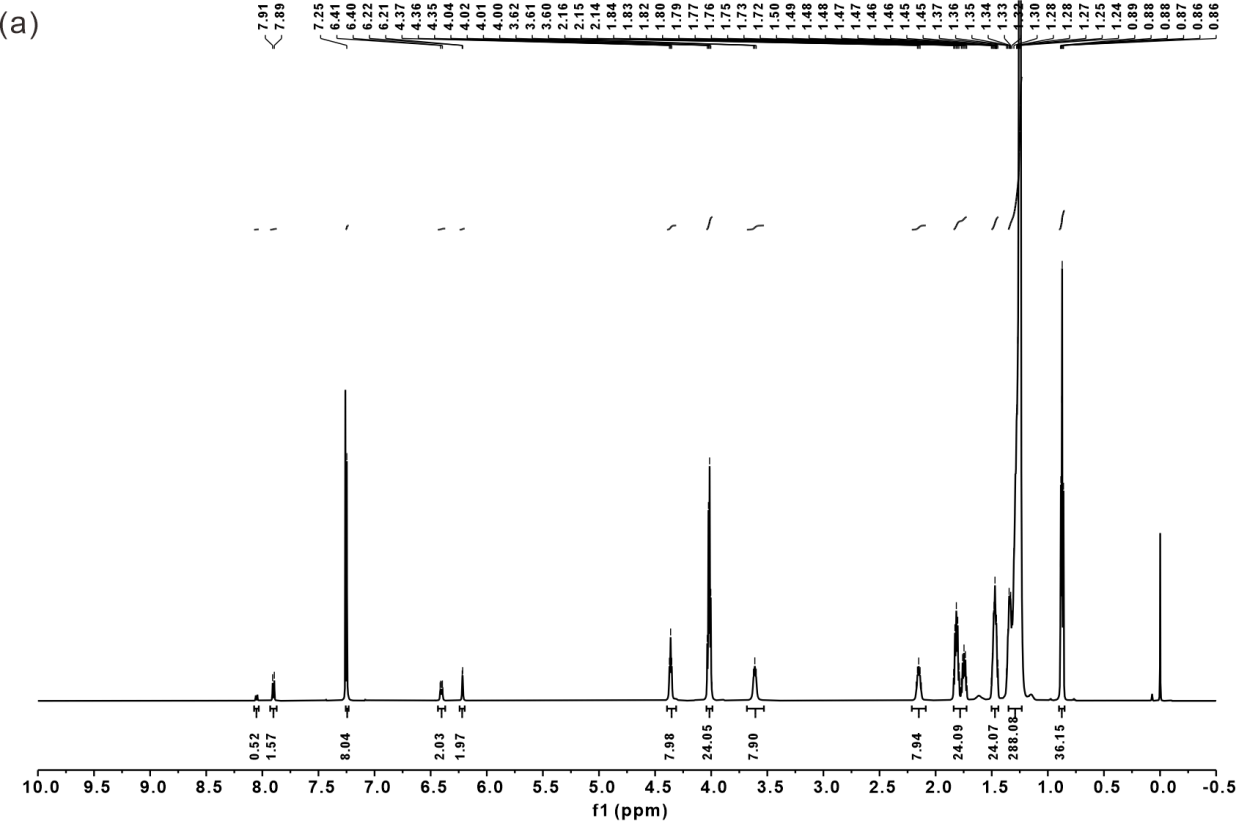


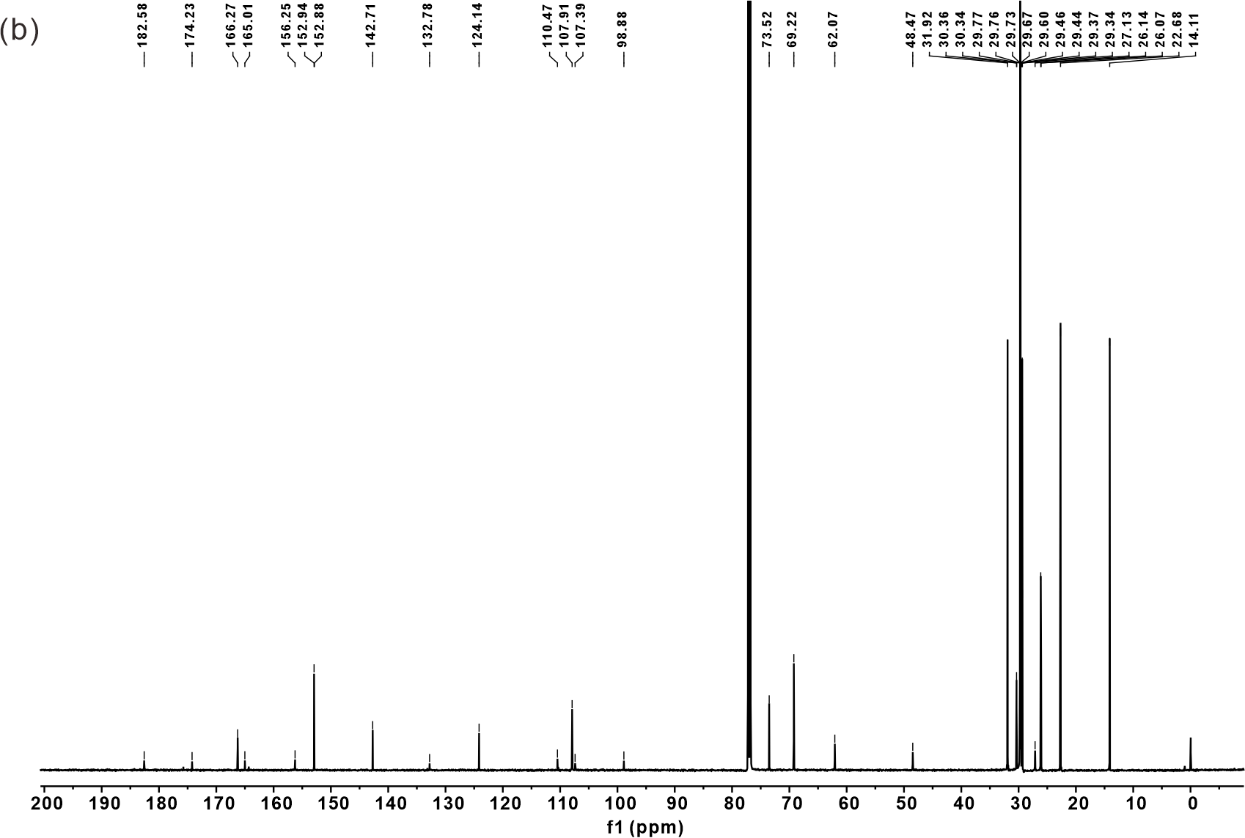


**Figure S5.** **SQ16**: (a) **^1^H NMR** spectrum (600 MHz, CDCl_3_, 298 K); (b) **^13^C NMR** spectrum (151 MHz, CDCl_3_, 298 K).

**6) SQ18**

**SQ18** was synthesized from compound **E18** using a similar procedure of synthesizing compound **SQ8**. Yield: 33%. **^1^H NMR** (600 MHz, Chloroform-*d*) δ 8.05 (d, *J* = 9.1 Hz, 1H), 7.90 (d, *J* = 9.1 Hz, 2H), 7.25 (s, 8H), 6.40 (d, *J* = 9.1 Hz, 2H), 6.21 (s, 2H), 4.36 (t, *J* = 6.0 Hz, 8H), 4.02 (q, *J* = 6.3 Hz, 24H), 3.61 (s, 8H), 2.23 – 2.09 (m, 8H), 1.78 (ddt, *J* = 41.0, 14.6, 6.5 Hz, 24H), 1.51 – 1.44 (m, 24H), 1.41 – 1.13 (m, 336H), 0.87 (t, *J* = 7.0 Hz, 36H). **^13^C NMR**(151 MHz, CDCl_3_) δ 182.59, 174.23, 166.27, 165.02, 156.26, 152.94, 152.77, 142.71, 124.15, 110.48, 107.91, 107.39, 98.89, 73.53, 73.47, 69.22, 69.15, 62.07, 48.47, 31.92, 30.37, 30.31, 29.78, 29.76, 29.74, 29.72, 29.68, 29.67, 29.64, 29.61, 29.59, 29.56, 29.47, 29.41, 29.37, 29.35, 29.30, 27.13, 26.14, 26.08, 26.05, 26.03, 22.68, 14.11. **HRMS-ESI** m/z 4166.6976 ([M+H]^+^[C_272_H_486_N_2_O_24_H]^+^, calc. 4166.6949). **Elemental analysis** found: C 78.36%; H 11.62%; N 0.58%. C_272_H_486_N_2_O_24_. Required: C 78.37%; H 11.75%; N 0.67%.


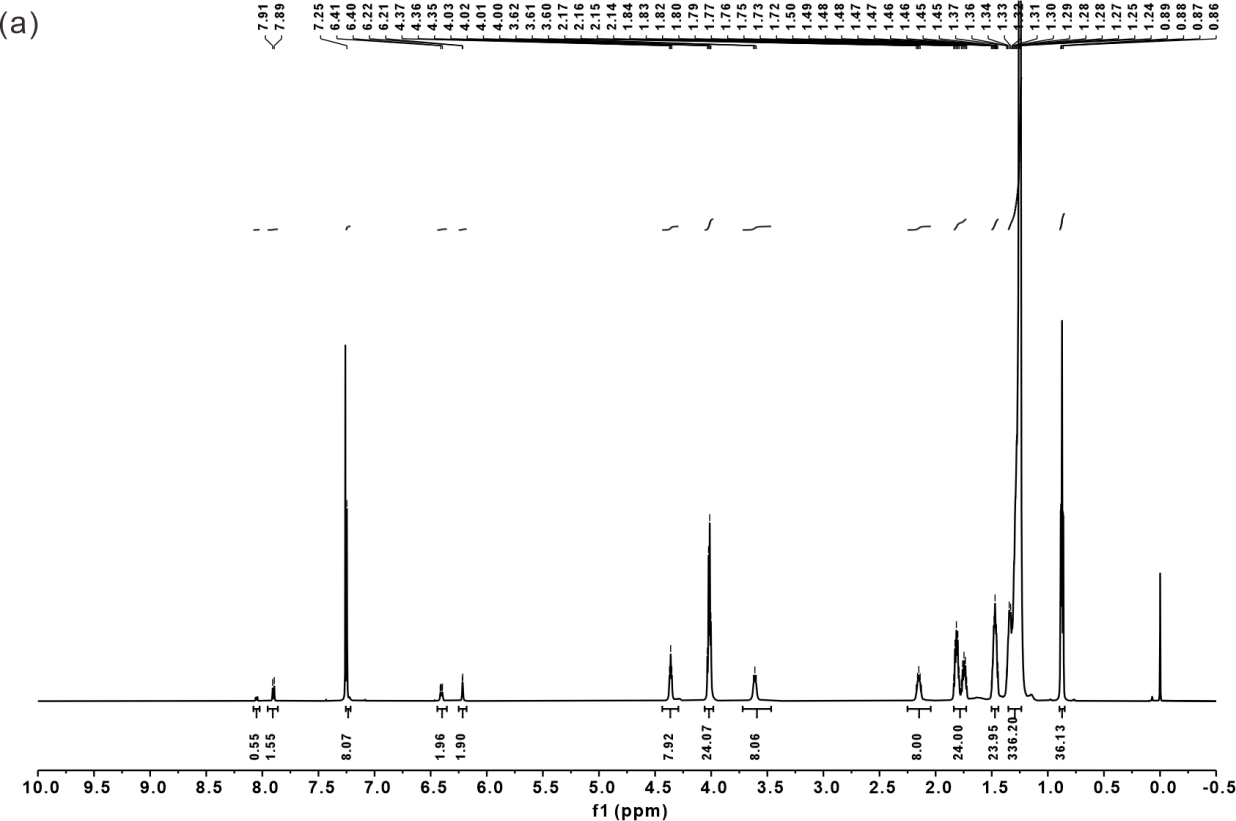


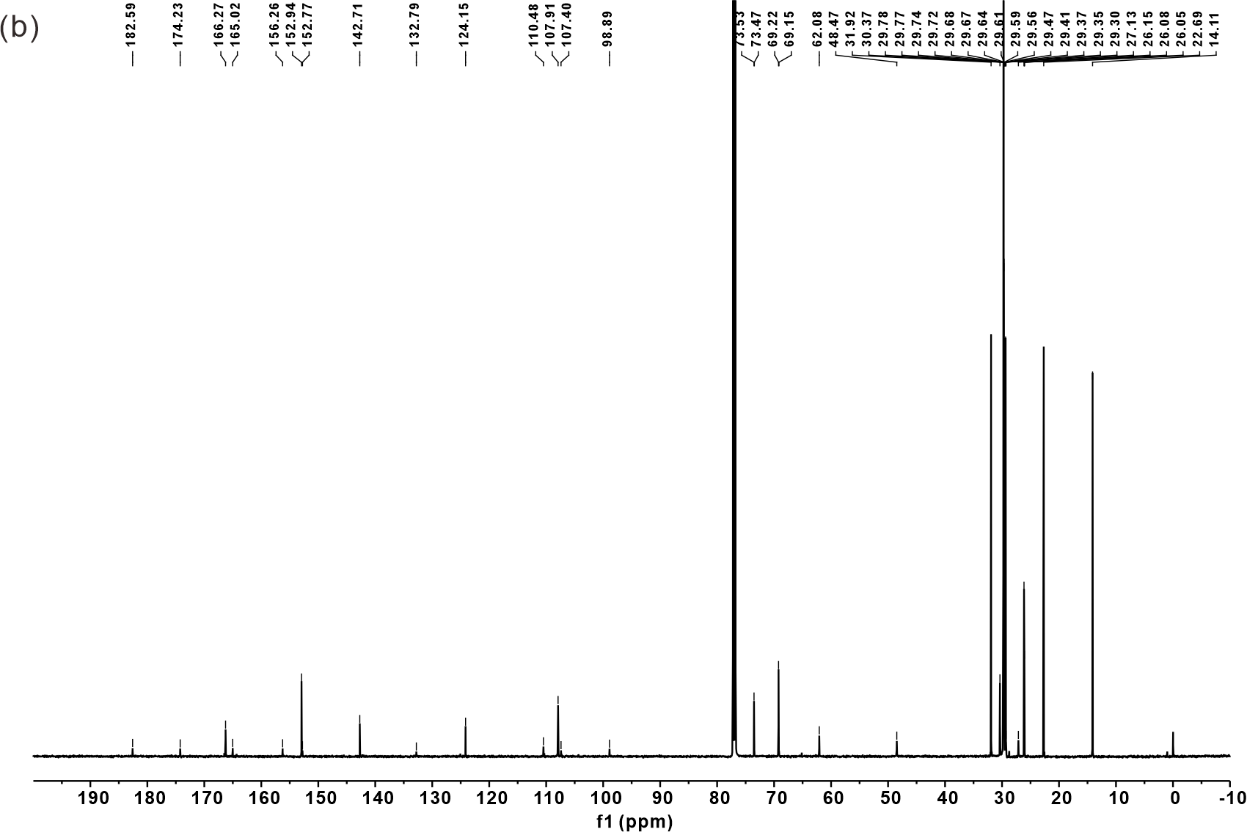


**Figure S6.** **SQ18**: (a) **^1^H NMR** spectrum (600 MHz, CDCl_3_, 298 K); (b) **^13^C NMR** spectrum (151 MHz, CDCl_3_, 298 K).

# 3. DSC traces, transition enthalpies of SQ*n* and their mixtures


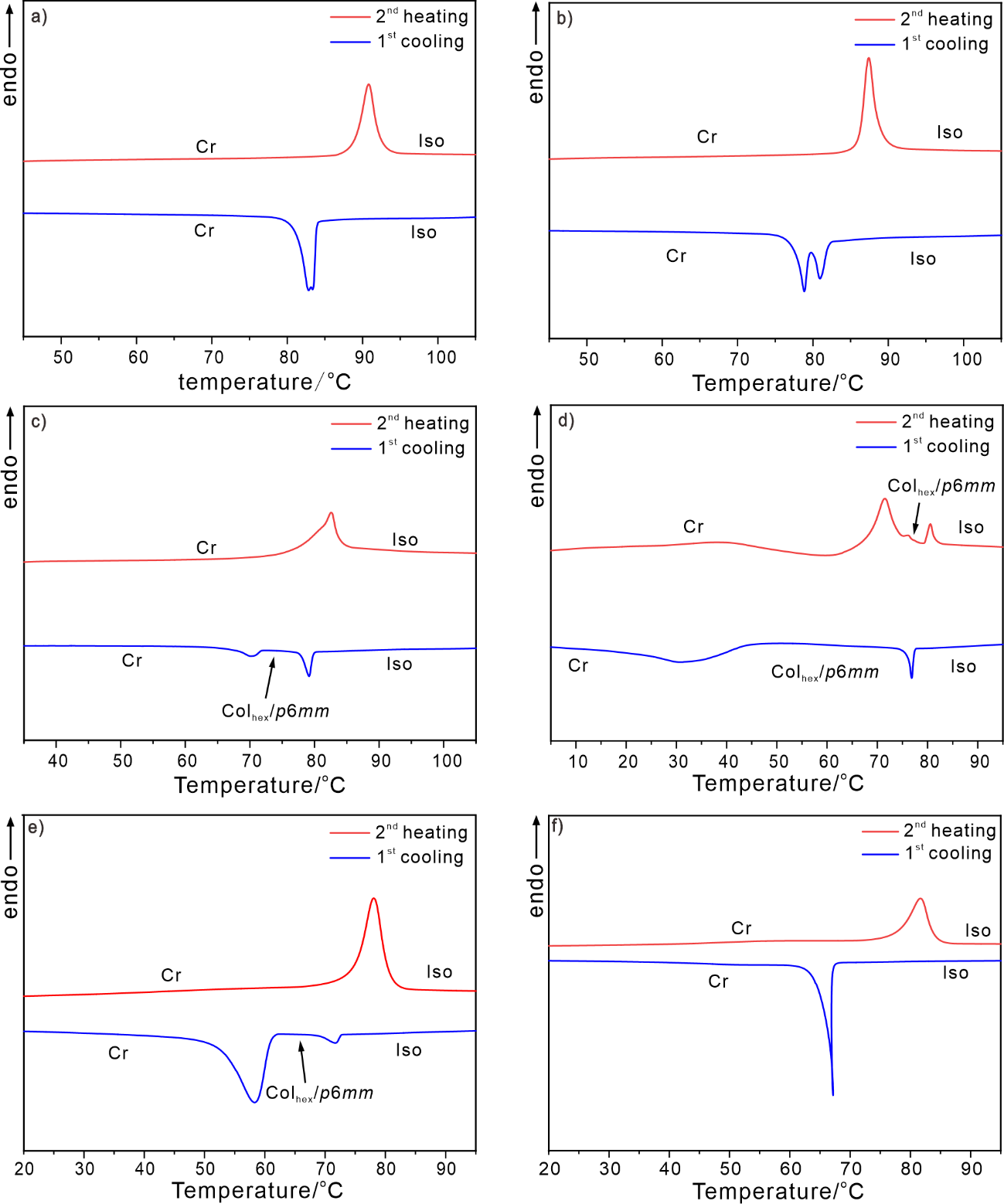


**Figure S7.** DSC heating and cooling traces for compounds: (a) **SQ8**; (b) **SQ10**; (c) **SQ12**; (d) **SQ14**;(e) **SQ16** and (f) **SQ18** recorded at 10 K/min.

**Table S1.** Mesophases, transition temperatures (*T*/℃) and associated enthalpies values of **SQ*n***

| Comp. | Heating scan  *T*/℃ [Δ*H* J/g] ^a^ | Cooling scan  *T*/℃ [Δ*H* J/g] ^a^ |
| --- | --- | --- |
| **SQ8** | Cr 84 [11.8] Iso | Iso 89 [13.3] Cr |
| **SQ10** | Cr 86 [11.6] Iso | Iso 80 [8.5] Cr |
| **SQ12** | Cr 80 [6.6] Iso | Iso 80 [3.0] Col_hex_/*p*6*mm* 72 [1.9] Cr |
| **SQ14** | Cr 68 [11.8] Col_hex_/*p*6*mm* 80 [1.8] Iso | Iso 78 [2.2] Col_hex_/*p*6*mm* 44 [13.9] Cr |
| **SQ16** | Cr 75 [36.1] Iso | Iso 73 [1.4] Col_hex_/*p*6*mm* 61 [36.7] Cr |
| **SQ18** | Cr 78 [50.0] Iso | Iso 67 [56.8] Cr |

^a^ Onset temperatures were determined from the first cooling scan and the second heating scan at 10 K/min. Abbreviations: Iso = isotropic liquid, Col_hex_ **=** hexagonal columnar phase with plane group *p*6*mm*, Cr = crystal solid.


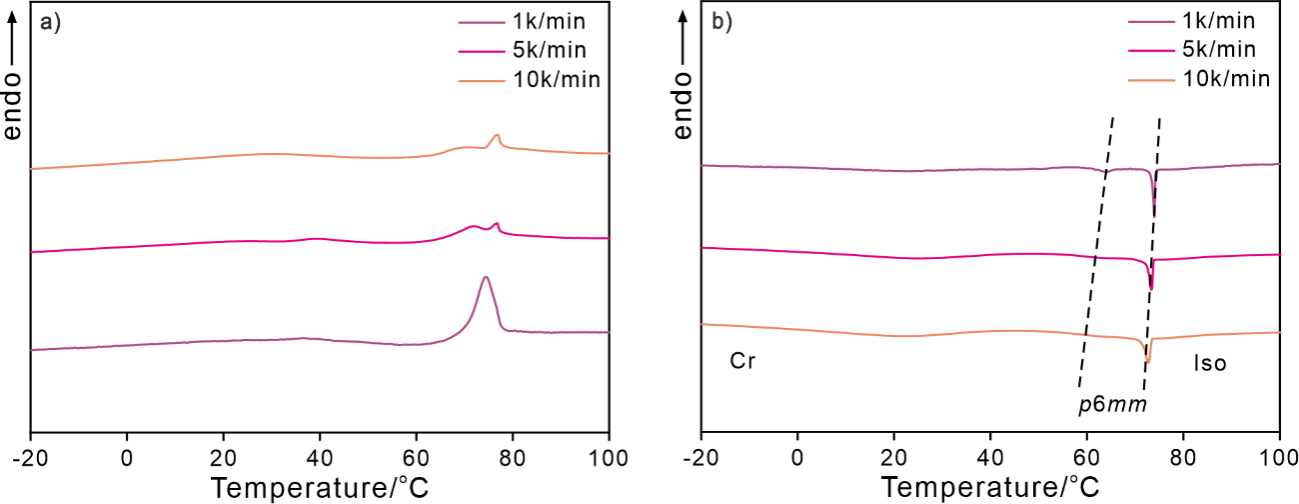


**Figure S8.** DSC traces of mixtures **SQ8/16** of (a) heating and (b) cooling using different heating/cooling rates as 1/5/10 K/min.


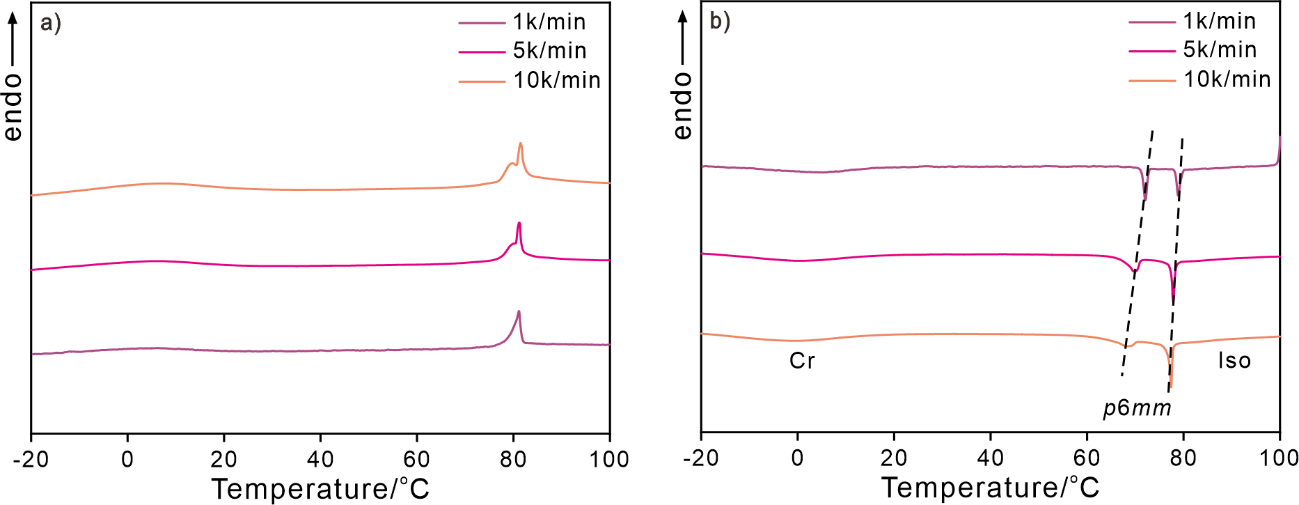


**Figure S9.** DSC traces of mixtures **SQ10/14** on (a) heating and (b) cooling using different heating/cooling rates as 1/5/10 K/min.


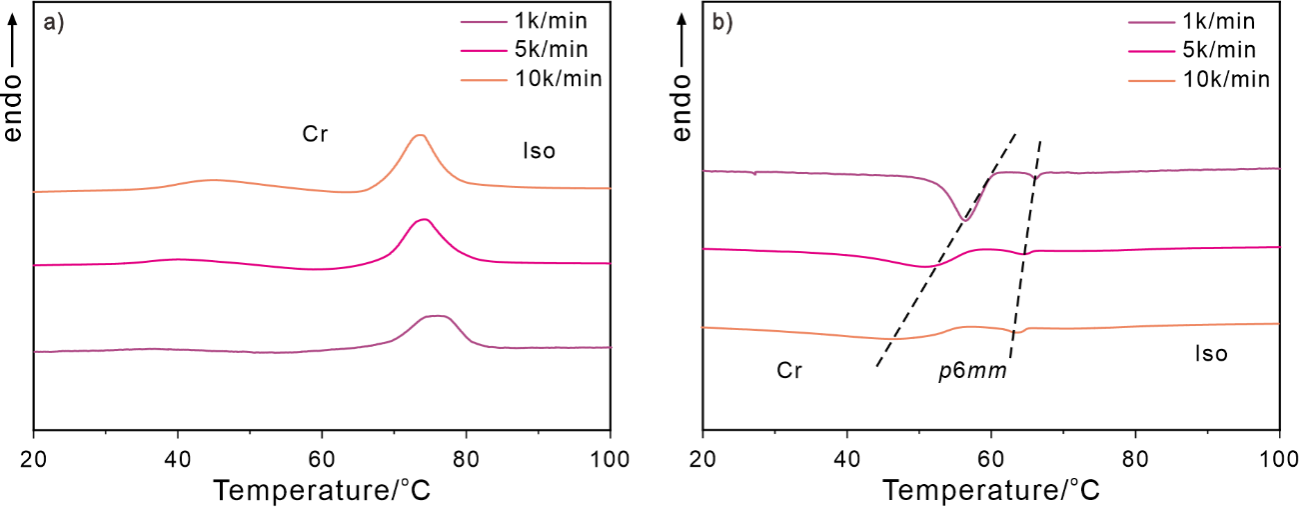


**Figure S10.** DSC traces of mixtures **SQ10/18** recorded in (a) heating process and (b) cooling process using different heating/cooling rates as 1/5/10 K/min.


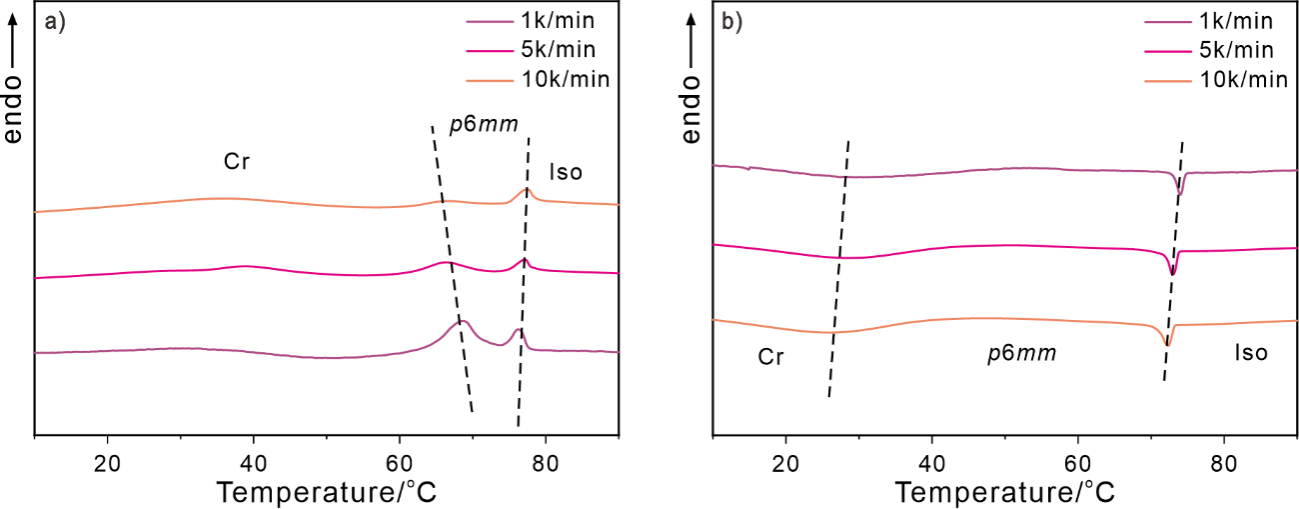


**Figure S11.** DSC traces of mixtures **SQ12/16** recorded in (a) heating process and (b) cooling process using different heating/cooling rates as 1/5/10 K/min.


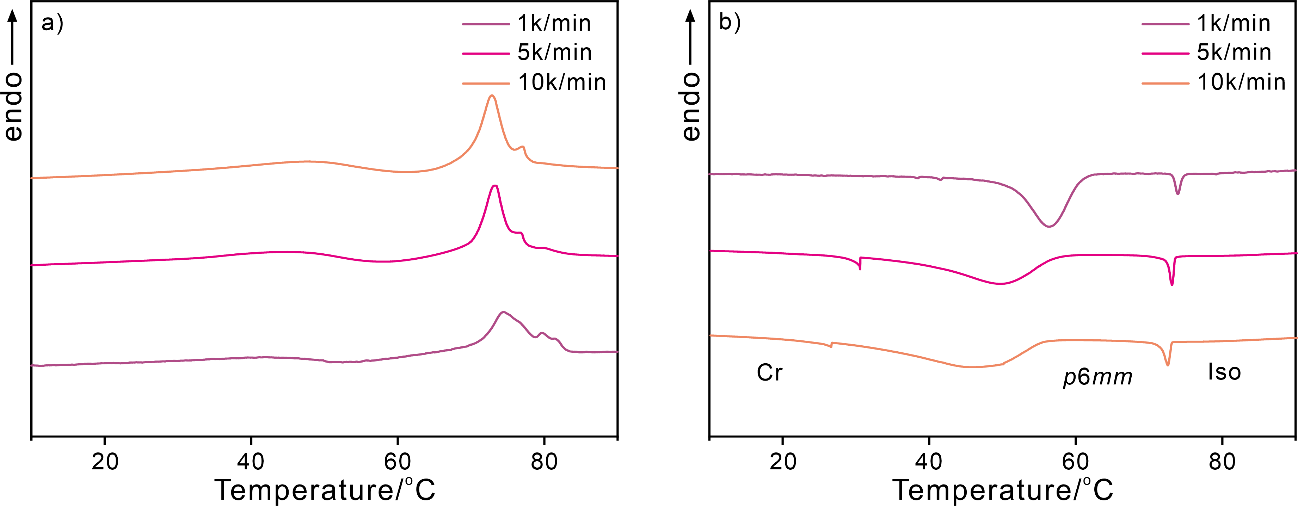


**Figure S12.** DSC traces of mixtures **SQ14/18** recorded in (a) heating process and (b) cooling process using different heating/cooling rates as 1/5/10 K/min.

**Table S2.** LC temperature ranges on cooling (*T*/℃) and associated enthalpies values of **SQ*n*** and their mixtures(recorded at 10 K/min.)

| Comp. | LC temperature range  *T*/℃ | Iso- Col_hex_/*p*6*mm*  J/g |
| --- | --- | --- |
| **SQ12** | 72 - 80 ℃ | 3.0 |
| **SQ8/SQ16** | 64 - 76 ℃ | 1.8 |
| **SQ10/SQ14** | 72 - 80 ℃ | 2.1 |
| **SQ14** | 44 - 78 ℃ | 2.2 |
| **SQ10/SQ18** | 56 - 66 ℃ | 0.6 |
| **SQ12/SQ16** | 45 - 77 ℃ | 1.8 |
| **SQ16** | 61 - 73 ℃ | 1.4 |
| **SQ14/SQ18** | 58 - 72 ℃ | 1.7 |

# 4*.* Additional optical micrographs and simulations

## 4.1 Additional optical textures for SQ*n*

**
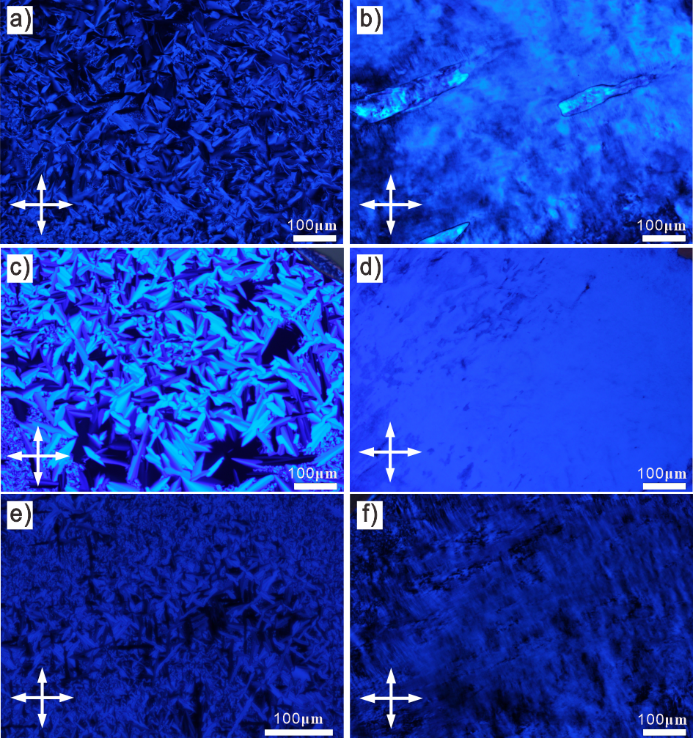
**

**Figure S13.** Textures of **SQ12** at 75 ℃ before (a) and after shearing (b); Textures of **SQ14** at 70 ℃ before (c) and after shearing (d); Textures of **SQ16** at 65 ℃ before (e) and after shearing (f).


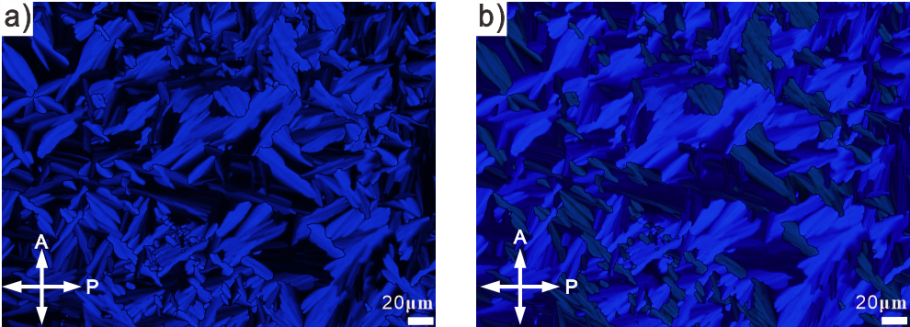


**Figure S14.** Textures of **SQ12** at 76 ℃ under different conditions (a) without a λ-plate; (b) with a 550 nm λ-plate.


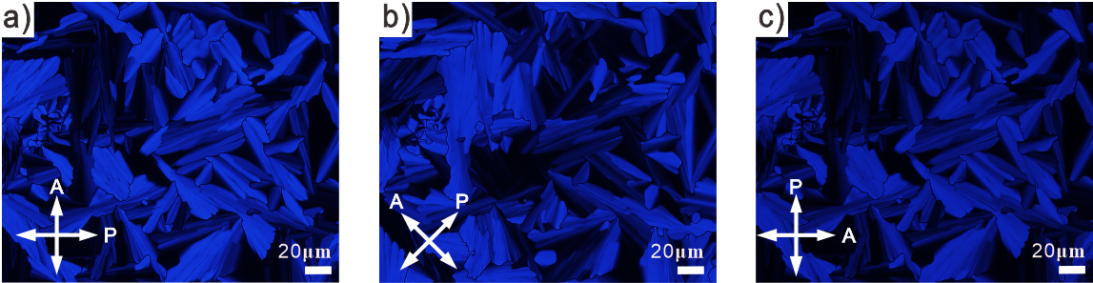


**Figure S15.** Textures of **SQ12** at 76 ℃ under different orientations (a) 0^o^; (b) 45^o^; (b) 90^o^, proving that dark regions are caused by well-alignment of molecules, rather than isotropic state.

# 5. Additional X-ray data

## 5.1 Synchrotron X-ray diffraction and electron density reconstruction

High-resolution small/wide-angle powder diffraction experiments were recorded on Beamline BL16B1 at Shanghai Synchrotron Radiation Facility, SSRF. Experiments were carried out on solid samples in 1 mm glass capillaries under the control of modified Linkam hot stage with a thermal stability within 0.2 ℃. The Pilatus 2M detector was applied in the experiments. *q* calibration and linearization were testified by using several orders of layer reflections from silver behenate and a series of *n*-alkanes. The diffraction peaks are indexed from the diffraction patterns, then the lattice parameters and the plane groups are subsequently determined. 2D electron density maps can be reconstructed according to diffraction intensities and the corresponding plane group. The general formula is as follows:

*E*(*xy*) = Σ*_hk_* *F*(*hk*)exp[*i*2π(*hx*+*ky*] Eqn. 1

In Eqn. 1, *F*(*hk*) is the structure factor of a diffraction peak with index (*hk*). It is normally a complex number and the experimentally observed diffraction intensity

*I*(*hk*) = *K*·*F*(*hk*)·*F**(*hk*) = *K*·|*F*(*hk*)|^2^ Eqn. 2

Here *K* is a constant related to the sample volume, incident beam intensity etc. In this paper we are only interested in the relative electron densities, hence this constant is simply taken to be 1. Thus, the electron density for 2D plane groups is:

*E*(*xy*) = Σ*_hk_* sqrt[*I*(*hk*)] exp[*i*2π(*hx*+*ky*)+*φ_hk_*] Eqn. 3

As the observed diffraction intensity, *I*(*hk*), is only related to the amplitude of the structure factor |*F*(*hk*)|, while the information about the phase of *F*(*hk*), *φ_hkl_*, cannot be determined directly from experiments. However, the problem is simplified as the structure of the ordered phase is centrosymmetric, and hence the structure factor *F*(*hk*) is always real and *φ_hk_* is either 0 or *π*. This makes it possible for a trial-and-error approach, where possible electron density maps are reconstructed for all possible phase combinations, and the “correct” phase combination is then selected on the merit of the maps, helped by prior physical and chemical knowledge of the system. This is especially useful for the study of nanostructures, where normally only a limited number of diffraction peaks are observed.

The Col_hex_/*p*6*mm* phase of **SQ12** is illustrated here to show how the proper phase combination is selected. The four peaks of the Col_hex_/*p*6*mm* phase, (10), (11), (20) and (21), are used for the reconstruction of the electron density maps, so possible phase combination from those four reflections is 2^4^ = 16. As shown in **Figure S16**, the phase combination 0000, π000, 0π00, 00π0, 000π, ππ00, π0π0, π00π are simple reversals of ππππ, 0πππ, π0ππ, ππ0π, πππ0, 00ππ, 0π0π, 0ππ0, respectively. Therefore, the number of different electron density maps can be reduced by half from sixteen to eight. According to the molecular structures and structural model, high electron density SQ units are randomly stacked on top of each other surrounded by a corona of hydrocarbon groups with low electron density. This coarse-grain reconstruction already gives the main features of the structure, indicating only the phase combination (00ππ) meets the structural constraints.


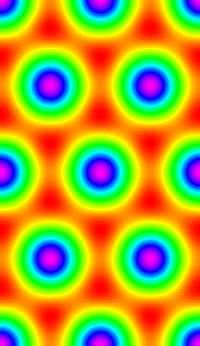

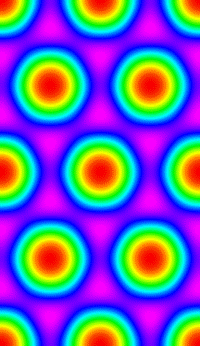

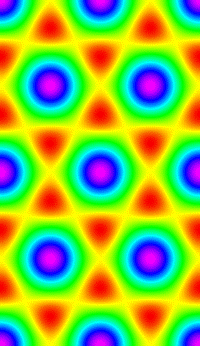

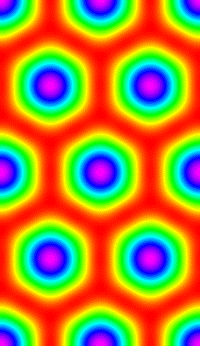


(0000) (π000) (0π00) (00π0)


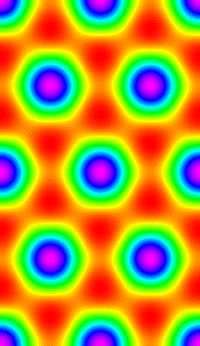

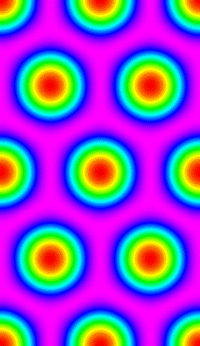

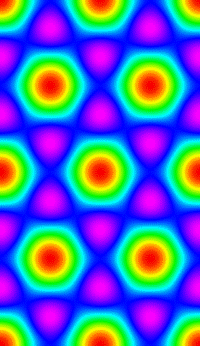

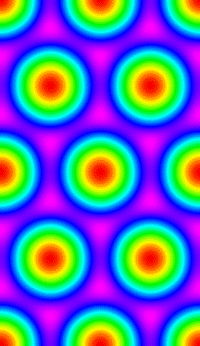


(000π) (ππ00) (π0π0) (π00π)


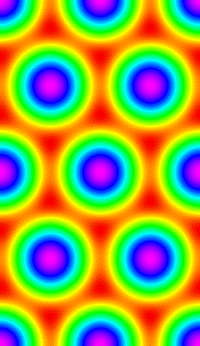

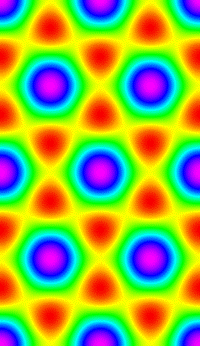

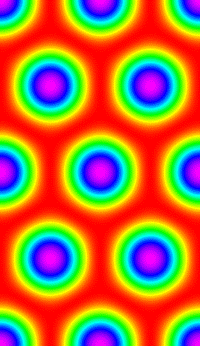

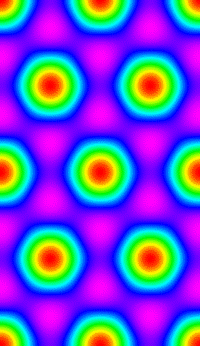


(0ππ0) (0π0π) (00ππ) (πππ0)


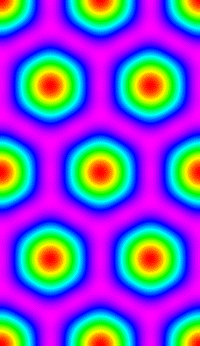

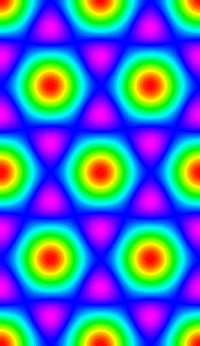

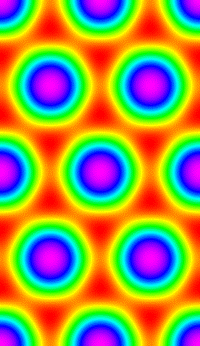

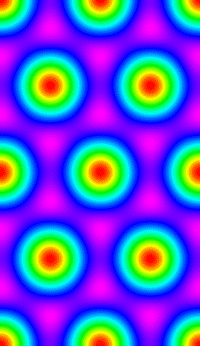


(ππ0π) (π0ππ) (0πππ) (ππππ)

**Figure S16.**All possible phase combinations of reconstructed density maps of the Col_hex_/*p*6*mm* phase of **SQ12** (purple for high ED regions and red for the low ED regions).

**5.2 SAXS/WAXS/GISAXS data**

**Table S3.** Experimental and calculated *d*-spacings, relative integrated intensities, and phases used in the reconstruction of electron densities for the Col_hex_/*p*6*mm* phase of **SQ12** at 76 ℃. All intensity values are Lorentz and multiplicity corrected.

| (*hk*) | *d*_obs_- spacings (nm) | *d*_cal_ - spacings (nm) | *Intensity* | *Phase* |
| --- | --- | --- | --- | --- |
| (10) | 3.75 | 3.75 | 100.0 | 0 |
| (11) | 2.16 | 2.16 | 0.3 | 0 |
| (20) | 1.87 | 1.87 | 0.3 | π |
| (21) | 1.41 | 1.42 | 0.03 | π |
| *a*_hex_= 4.33 nm | | | | |

**
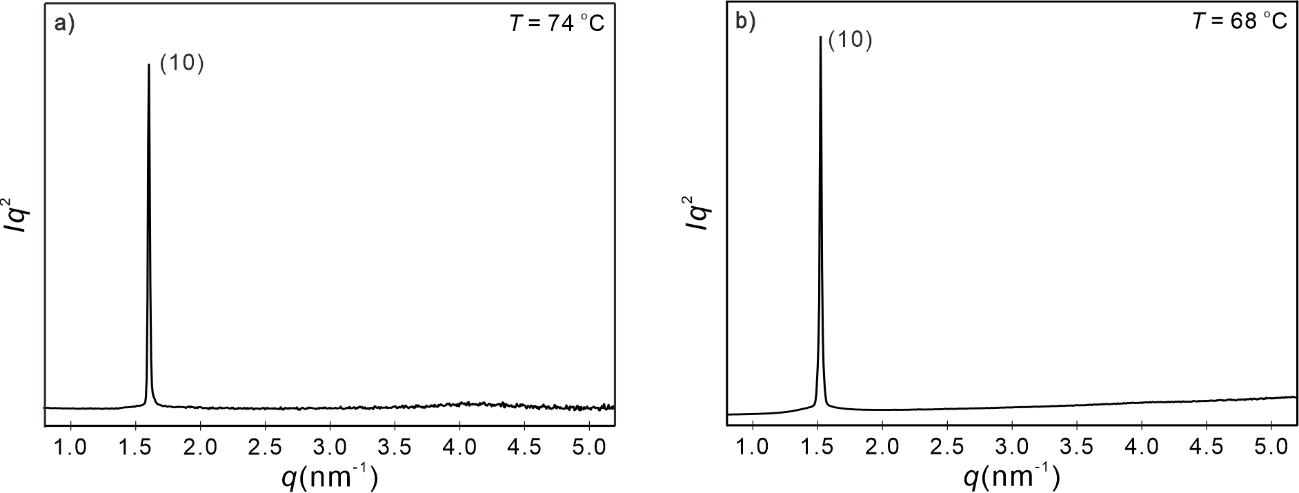
**

**Figure S17.** SAXS diffractograms of (a) **SQ14** at 74 ℃; (b) **SQ16** at 68 ℃. *a*_hex_ of **SQ14** and **SQ16** can be calculated as 4.51 nm and 4.77 nm respectively at the indicated temperature.


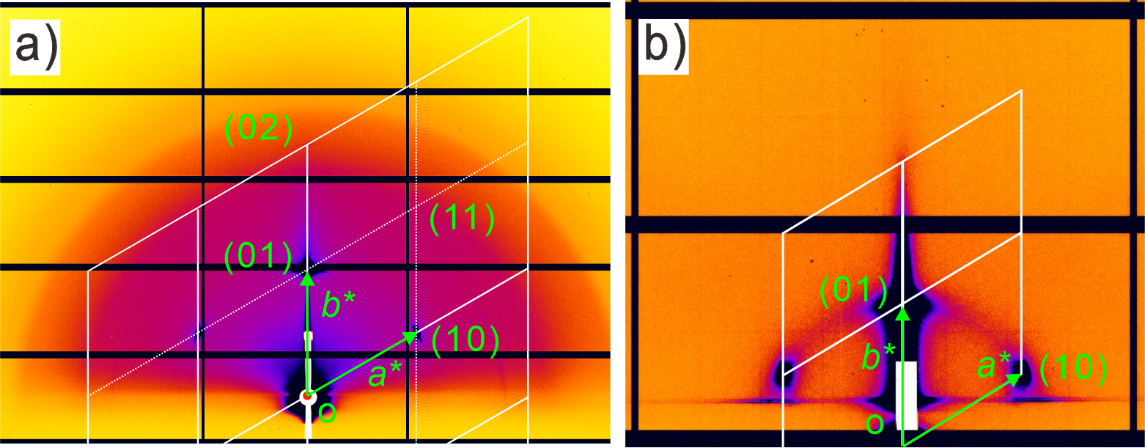


**Figure S18.** GISAXS patterns of (a) **SQ14** at70 ℃; (b) **SQ16** at 70 ℃.


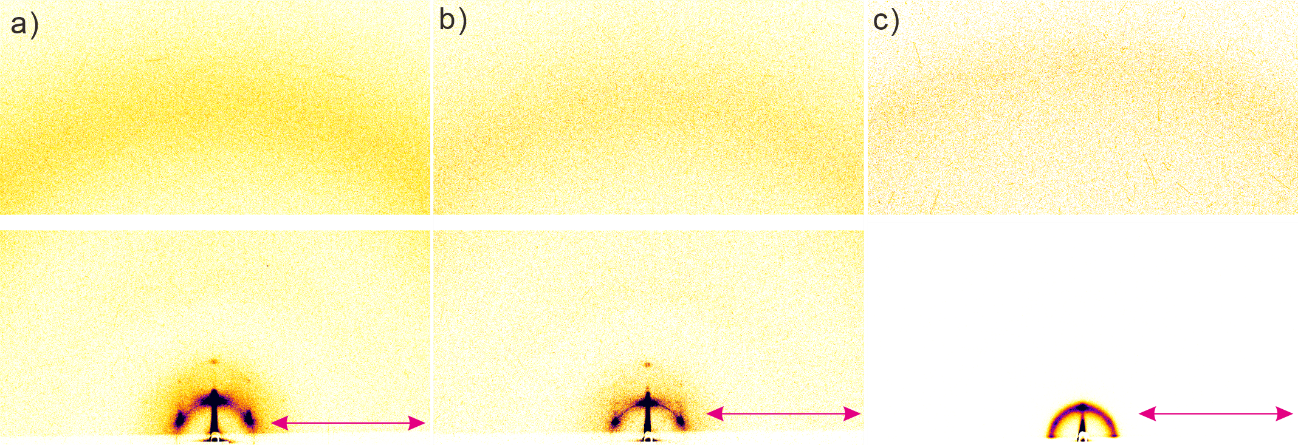


**Figure S19.** GIWAXS patterns of (a) **SQ12** at70 ℃; (a) **SQ14** at70 ℃; (b) **SQ16** at 65 ℃, the pink arrows indicate the shearing direction of samples.


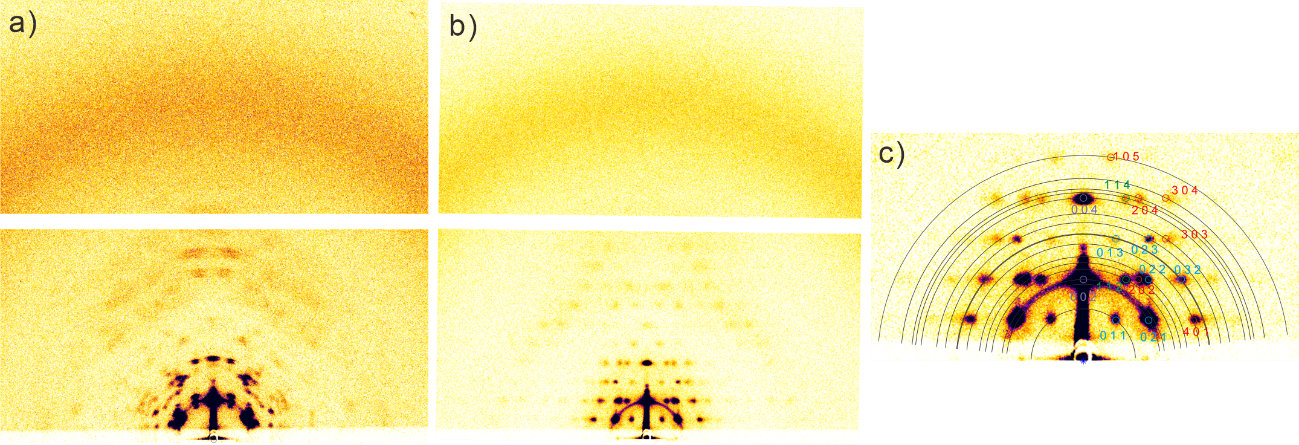


**Figure S20.** GIWAXS patterns of (a) M phase of **SQ12** at65 ℃; (b, c) orthorhombic phase at45 ℃ of **SQ14** with *a* = 10.9 nm, *b* = 9.1 nm, *c* = 7.3 nm, *α* = *β* = *γ* = 90^o^. We could estimate each unit cell containing ~135 molecules.

**
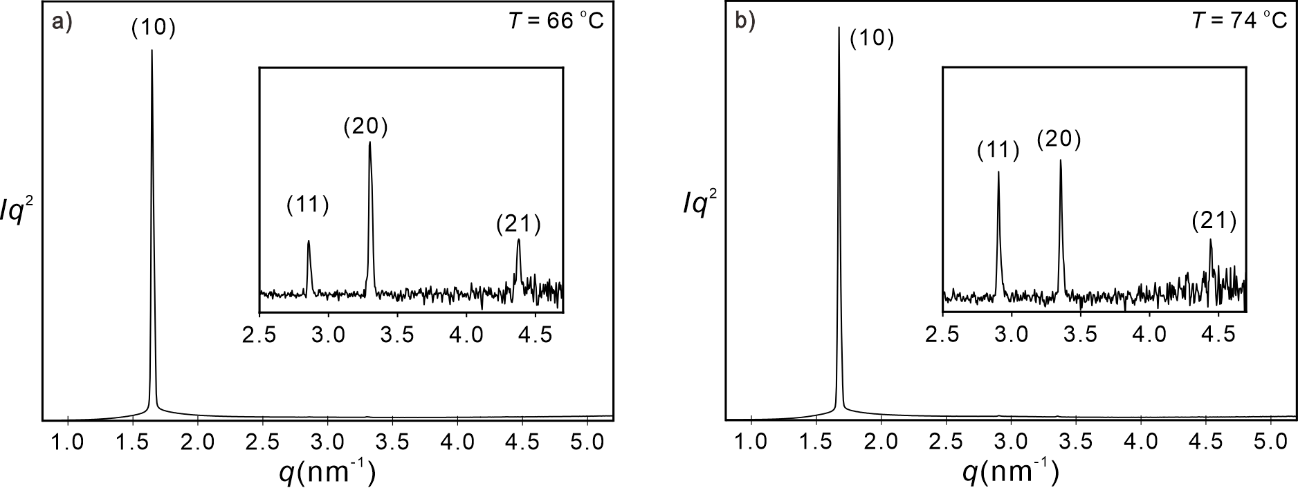
**

**Figure S21.** SAXS diffractograms of (a) **SQ8/16** at 66 ℃; (b) **SQ10/14** at 74 ℃ on cooling. *a*_hex_ of **SQ8/16** and **SQ10/14** can be calculated as 4.40 nm and 4.33 nm respectively at the indicated temperature.

**
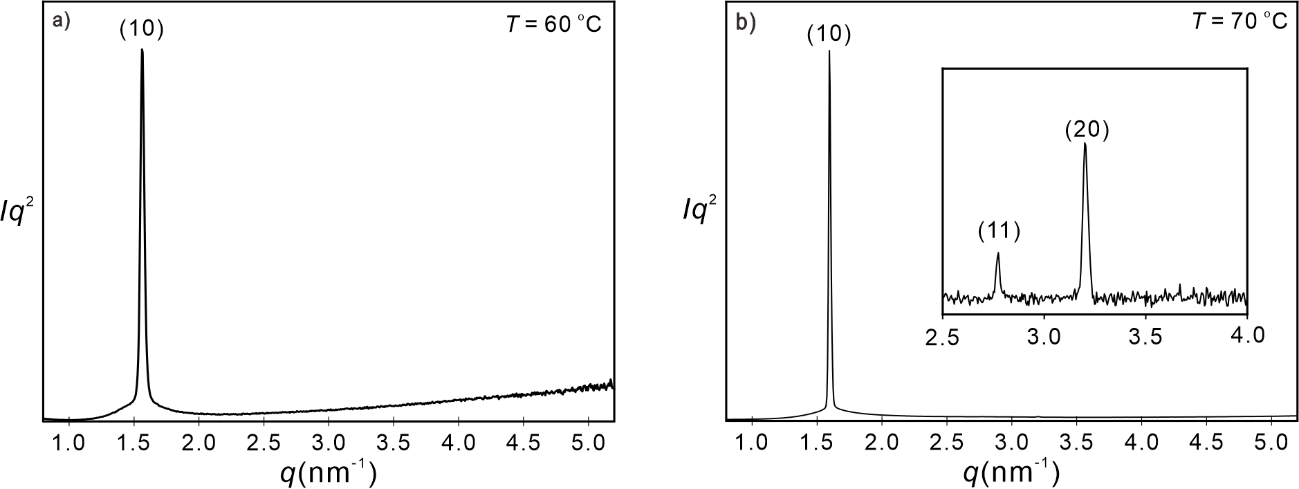
**

**Figure S22.** SAXS diffractograms of (a) **SQ10/18** at 60 ℃; (b) **SQ12/16** at 70 ℃ on cooling. *a*_hex_ of **SQ8/16** and **SQ10/14** can be calculated as 4.63 nm and 4.54 nm respectively at the indicated temperatures.

**
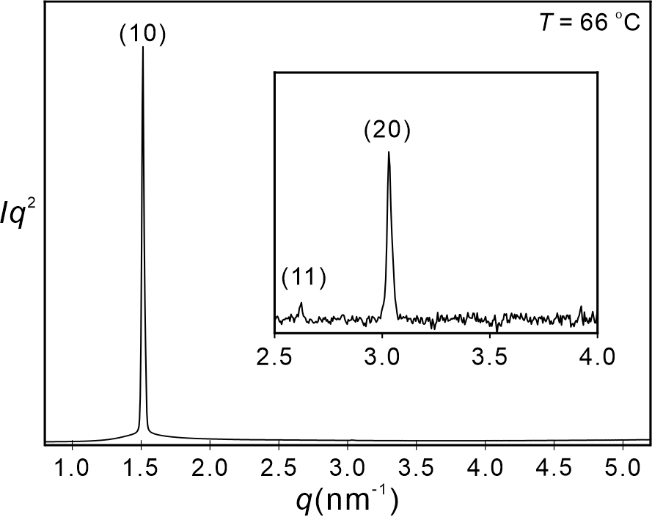
**

**Figure S23.** SAXS diffractogram of **SQ14/18** at 66 ℃; *a*_hex_ of **SQ14/18** can be calculated as 4.79 nm at the indicated temperatures.


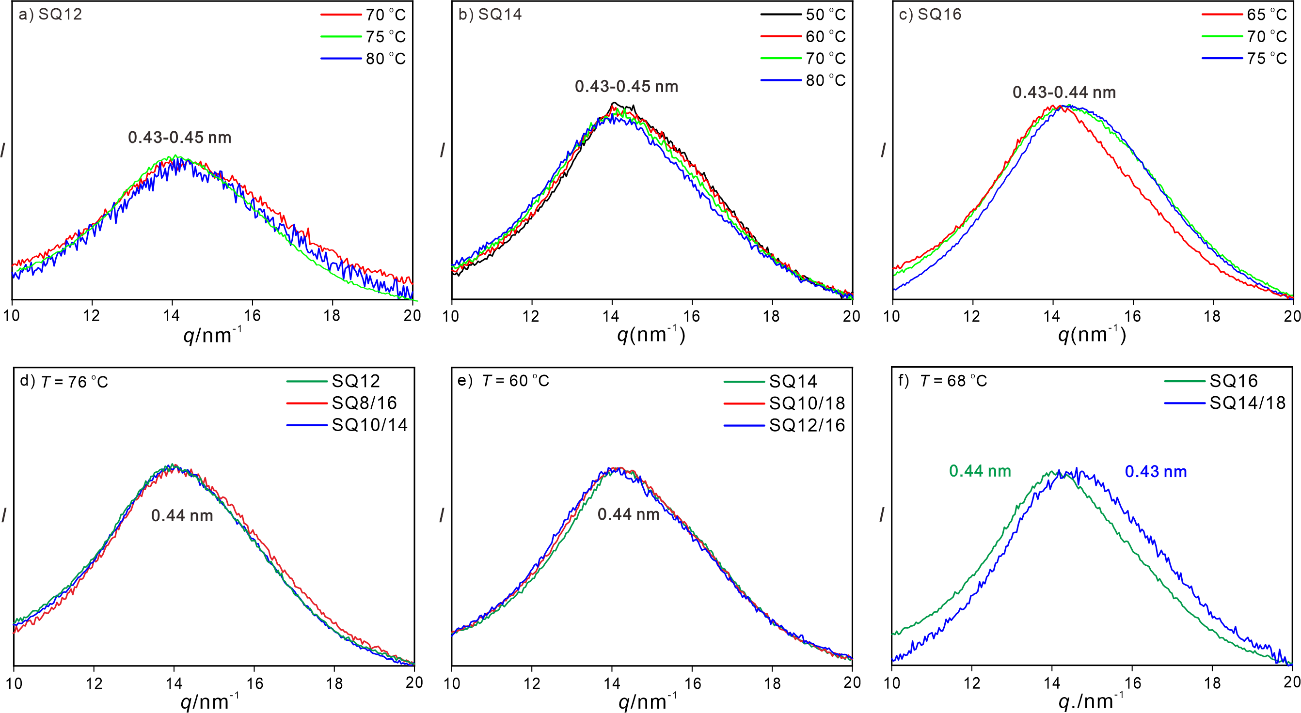


**Figure S24.** WAXS diffractograms of **SQ*n*** (***n*** =12, 14, 16)and their mixtures at the indicated temperature.


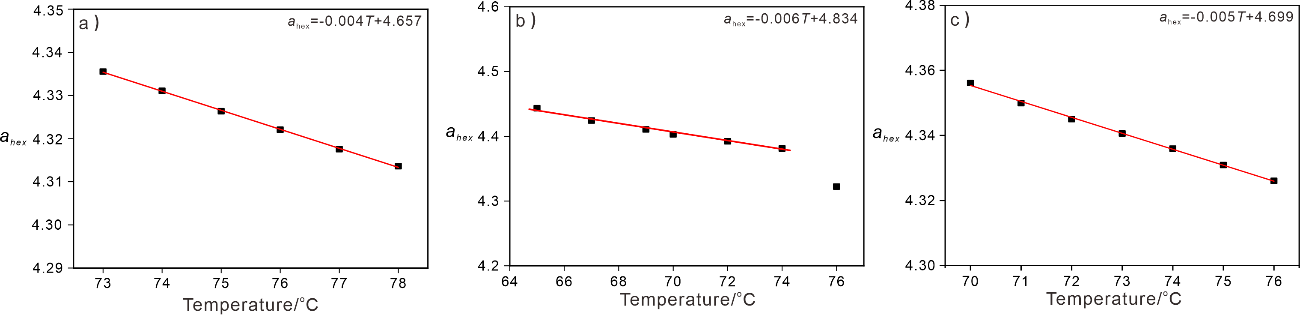


**Figure S25.** Temperature dependence of *a*_hex_ of (a) **SQ12** (b) **SQ8/16** and(c) **SQ10/14** on cooling; we could obtain *a*_hex_ of 4.33 nm, 4.32 nm and 4.33 nm for **SQ8/12**, **SQ8/16** and **SQ10/14** at 76 ℃ respectively.

**
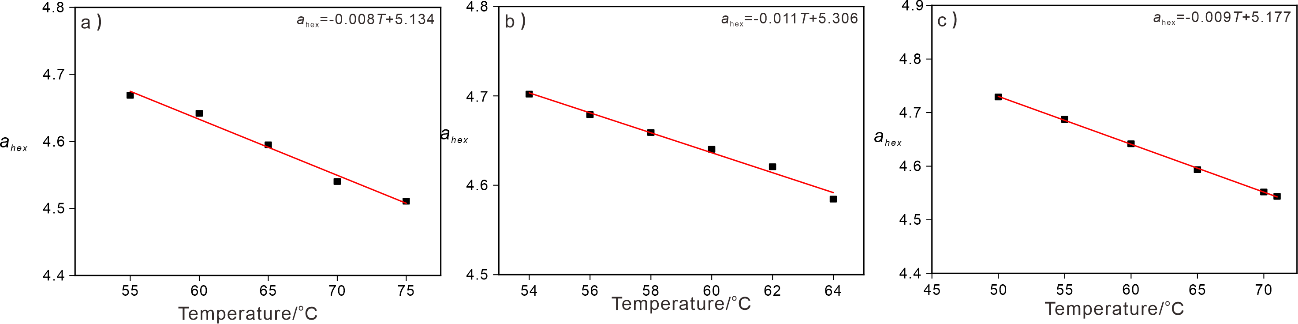
**

**Figure S26.** Temperature dependence of *a*_hex_ of (a) **SQ14** (b) **SQ10/18** and(c) **SQ12/16** on cooling; we could obtain *a*_hex_ of 4.64 nm for **SQ14**, 4.63 nm for **SQ8/16** and 4.64 nm for **SQ10/14** at 60 ℃ respectively.

**
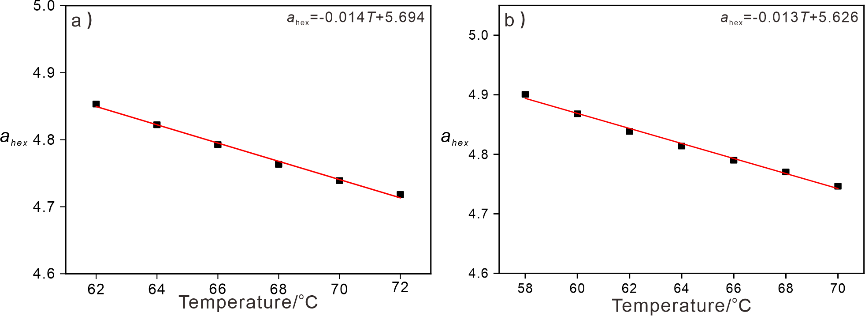
**

**Figure S27.** Temperature dependence of *a*_hex_ of **SQ16** and **SQ14/18** on cooling; we could obtain *a*_hex_ as 4.77 nm of **SQ16** and 4.78 nm of **SQ14/18** at 68 ℃.

Above results imply that phase structures are completely controlled by the aliphatic chain volume, because of the similar lattice parameters between mixtures and chain volume equivalent pure samples.

## 5.3 Additional electron density maps for SQ*n* mixtures

**
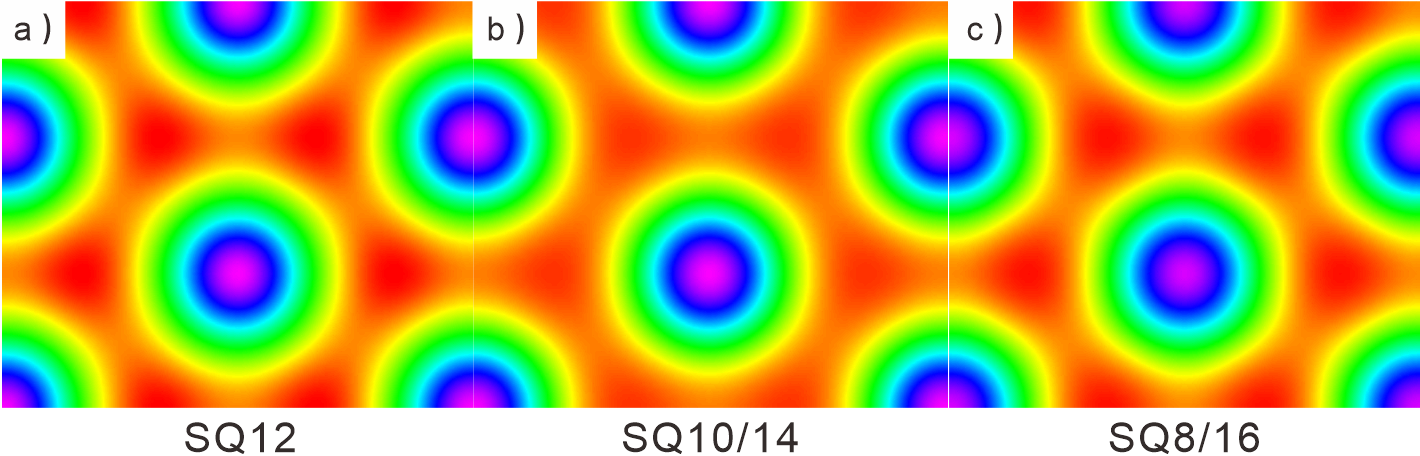
**

**Figure S28.** Normalized ED maps of samples (a) **SQ12**; (b) **SQ10/14** and (c) **SQ8/16**.


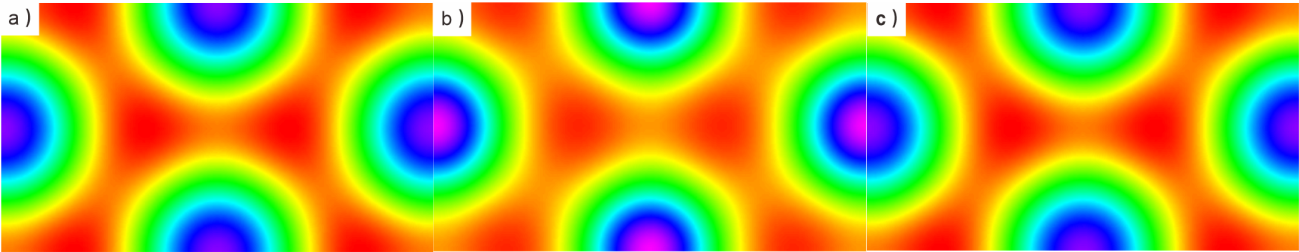


**Figure S29.** Normalized ED maps of samples (a) **SQ14**; (b) **SQ12/16** and (c) **SQ10/18**.

**
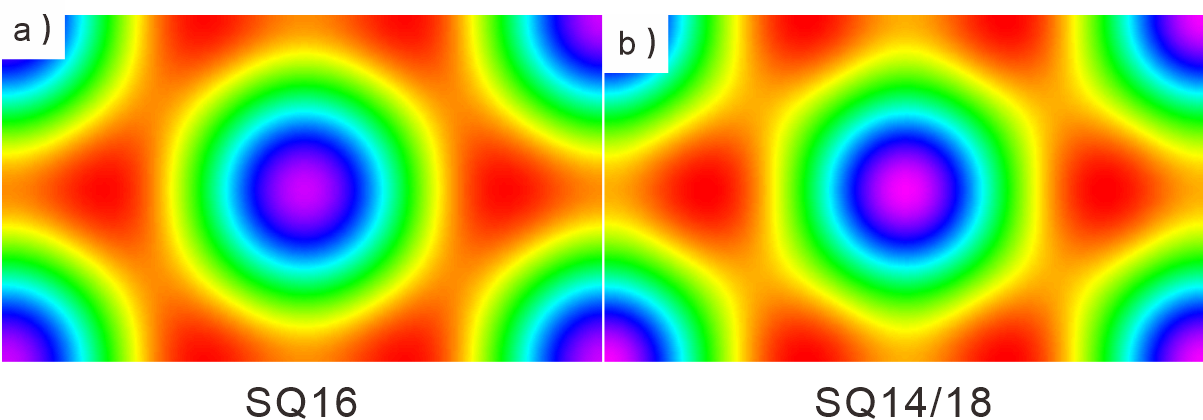
**

**Figure S30.** Normalized ED maps of samples (a) **SQ16**; (b) **SQ14/18**.

# 6. Calculation of geometrical parameters of molecule and Lattice parameters

## 6.1 Calculated molecular volume, volume fractions of molecular segments


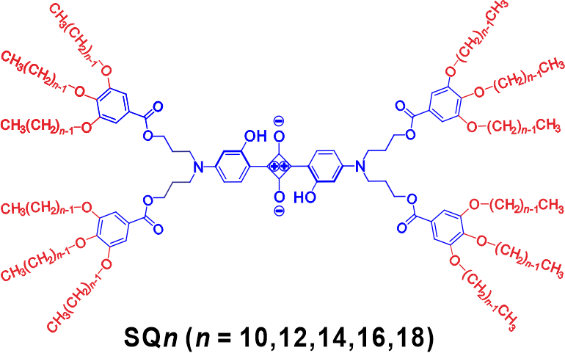


**Figure S31.**Molecular structure and molecular segments.

**Table S4.** The length, volume and fraction of molecular segments^a^.

| Comp. | *L*_core_  (nm) | *L*_alkyloxy_  (nm) | *L_N-N_*  (nm) | *L*_molecule_  (nm) | *W*_molecule_  (nm) | *V*_core_*^b^*  (nm^3^) | *V_alkyloxy_*^b^  (nm^3^) | *V_total_^b^*  (nm^3^) | *V*_core_% |
| --- | --- | --- | --- | --- | --- | --- | --- | --- | --- |
| **SQ8** | 3.4 | 1.2 | 1.5 | 5.7 | 2.4 | 1.0 | 2.6 | 3.5 | 28.6 |
| **SQ10** |  | 1.4 |  | 6.2 | 2.6 |  | 3.2 | 4.2 | 23.8 |
| **SQ12** |  | 1.6 |  | 6.7 | 2.8 |  | 3.8 | 4.7 | 21.3 |
| **SQ14** |  | 1.8 |  | 7.2 | 3.0 |  | 4.4 | 5.3 | 18.9 |
| **SQ16** |  | 2.0 |  | 7.7 | 3.1 |  | 5.0 | 5.9 | 16.9 |
| **SQ18** |  | 2.2 |  | 8.2 | 3.3 |  | 5.6 | 6.5 | 15.4 |

*^a^**L*_core_= the length of the core of the SQ molecules in an all-trans conformation (the blue part in **Figure S31**); *L*_alkyloxy_= the length of alkyloxy groups of SQ molecules in trans conformations (the red part in **Figure S31**); *L_N-N_*= the length of SQ chromophores between two terminal amino groups; *L*_molecule_ = the length of molecules; *W*_molecule_= the width of molecules; *V*_core_ , *V_alkyloxy_*,*V_total_* represent the volume of core parts, the volume of alkyloxy parts and the total volume of the molecule, respectively. ^b^*V* is the volume of molecular segments calculated by using the method of organic crystal increments^S3^.

## 6.2 Calculation of the average number of molecules in “unit cell”

*V_unit cell_=*$\frac{\text{√3}}{\text{2}}\text{a}^{\text{2}}\text{×}\text{h}$

*n_cell, crys_= V_unit cell_* / *V_molecules_*

*n_cell, liq_= (n_cell, crys_* / 0.7)$\text{ ×}\text{ }$0.55

*n_cell, LC_= (n_cell, crys_+ n_cell, liq_)* / 2

**Table S5.** Number of the molecules in each “unit cell”.

| Comp. | *a_hex_*  (nm) | *h*  (Å) | *V_molecules_*  (nm^3^) | *V_unit cell_* (nm^3^) | *n_cell, crys_* | *n_cell, liq_* | *n_cell, Lc_* |
| --- | --- | --- | --- | --- | --- | --- | --- |
| **SQ12** | 4.31-4.34 | 4.4 | 4.74 | 7.08-7.18 | 1.5 | 1.2 | 1.4 |
| **SQ14** | 4.51-4.68 | 4.4 | 5.34 | 7.75-8.35 | 1.4-1.6 | 1.1-1.3 | 1.3-1.5 |
| **SQ16** | 4.72-4.85 | 4.4 | 5.93 | 8.49-8.96 | 1.4-1.5 | 1.1-1.2 | 1.3-1.4 |

*V_unit cell_* is the volume an “unit cell” of columnar phase, or a columnar stratum with height *h* as averaged molecular distance acquired from WAXS patterns (~ 0.4 nm). *V_molecule_* is the volume of one molecule calculated by using the method of organic crystal increments^S3^. *n_cell, crys_* is the average number of molecules in one unit cell of a crystal, while the *n_cell, liq_* is the average number of molecules in one unit cell of a “liquid state” by correcting the packing coefficient (the packing coefficients of a crystal and a liquid are 0.7 and 0.55, respectively^S4^). Moreover, it is reasonable to take the density of liquid crystals as 1 g/cm^3^, leading to ~1 molecule per unit cell, fitting well with the results calculated with the method of organic crystal increments.

## 6.3 Radial volume distribution curve


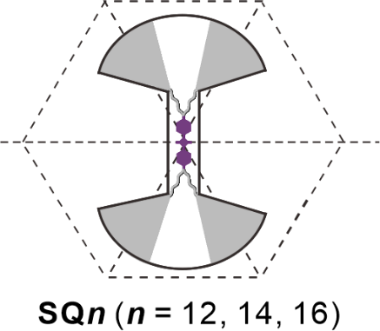


**Figure S32.** Molecular profile of **SQ*n*** (*n* =12, 14, 16).


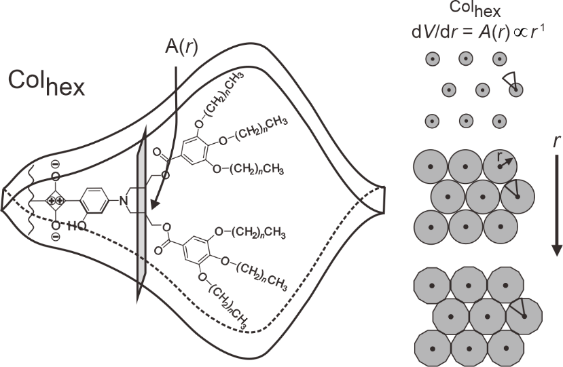


**Figure S33.** Illustration of d*V*/d*r* in the columnar phase.

The radial volume distribution (d*V*/d*r*) curves show the occupied volume increment as the size of phase increases. Generally, the d*V*/d*r* curves indicate the molecular shapes accommodating the phase structure. For the columnar phase (take **SQ12** as an example, *a* = *a*_hex_= 4.33 nm, see parameters of columnar phase):

$\frac{\text{d}\text{V}}{\text{d}\text{r}}\text{=2π}\text{hr}\text{ (0 ≤ }\text{r }\text{≤}{\text{ }\text{a}}/\text{2}\text{)}$ Eqn. 4

$\frac{\text{d}\text{V}}{\text{d}\text{r}}\text{=2π}\text{hr}\text{-12 }\text{r}\text{ }\text{arccos}\frac{\text{a}}{\text{2}\text{r}}\text{(}\text{a}/\text{2 }\text{< }\text{r}\text{ ≤ }\text{a}/{\sqrt{\text{3}}}$) Eqn. 5


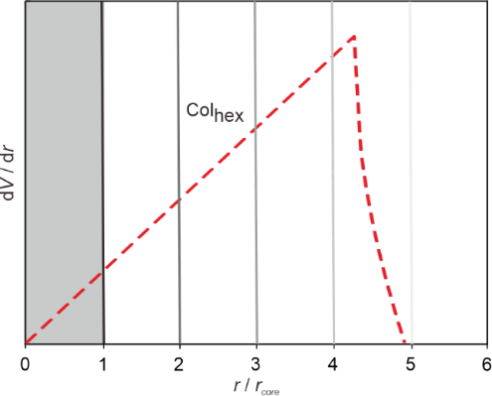


**Figure S34.** The d*V*/d*r* curve of columnar phase (Red dotted line), *r*_core_ = length of squaraine chromophores (1.5 nm).

## 6.4 Molecular dynamic simulation of molecule arrangement in columnar phase

Annealing dynamics runs were carried out using the Universal Force Field (Material Studio, Accelrys). Taking **SQ12** as an example, the structure in **Figure 1**d was obtained with 1 molecule in one “unit cell” with side equals to 4.33 nm and a height of 0.43 nm, then expanding to 3d crystals. 30 temperature cycles of NVT dynamics were run between 300 - 400 K, with a total annealing time of 30 ps for LC columnar phase. The authors thank Prof. Yumei Zhang at the College of Materials Science and Engineering, Donghua University, China, for allowing use of the Material Studio (Accelrys Inc.).

# 7. UV/vis absorption spectra and PL spectra

## 7.1 PL spectra of SQ*n*


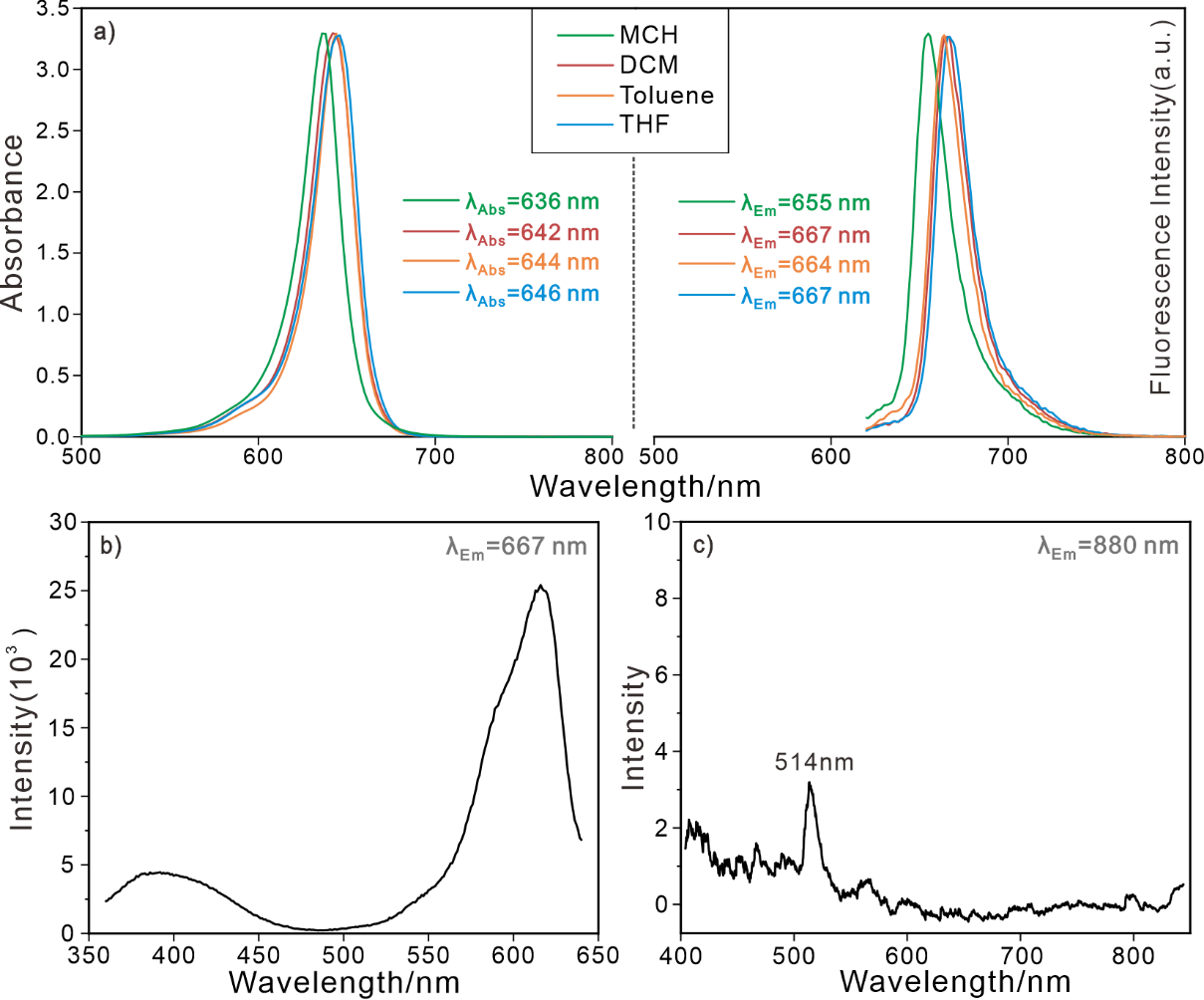


**Figure S35.** (a)Absorption (solid line) and emission (dotted line) spectra of **SQ12**, **SQ14** and **SQ16** in different solvents (*c* = 1$\times$10^-5^ M); Fluorescence excitation spectra of **SQ*n*** (b) in dilute solutions (*c* = 1$\times$10^-5^ M, DCM) and (c) as thin films.

**SQ*n*** compounds have limited solubility in *n*-hexane and highly polar solvents like acetonitrile and methanol, but soluble in methylcyclohexane (MCH), toluene, tetrahydrofuran (THF), dichloromethane (DCM) and chloroform. The fluorescence excitation spectra of **SQ*n*** thin films featuring weak fluorescence, prove the fluorescence quenching character of H-aggregates in SQ solids.

Photophysical parameters of **SQ12** in different organic solvents were collected in Table S6. Moreover, all **SQ*n*** compounds exhibit similar photophysical parameters (especially the same absorption and emission) in the same solvents, reflecting the gallate groups with different chain lengths have no effect on optical properties of SQchromophores. Furthermore, their fluorescence lifetimes and quantum yields are similar to each other, see Table S7.

**Table S6.** Photophysical parameters of **SQ12** in different organic solvents.

| Solvents | *ε*  M^-1^·cm^-1^ | *λ*_Abs_  nm (cm^-1^) | *λ*_onset_  nm | $\text{E}_{\text{g}}^{\text{opt}}$  eV | *λ*_Em_  nm(cm^-1^) | Δ*λ*  nm(cm^-1^) | *τ*_F_  ns | Ф_F_ |
| --- | --- | --- | --- | --- | --- | --- | --- | --- |
| MCH | 3.29$\text{×}$10^5^ | 636 (15723) | 661 | 1.87 | 655(15129) | 25(595) | 5.4 | 0.60 |
| DCM | 3.32$\text{×}$10^5^ | 642 (15576) | 671 | 1.85 | 667(14903) | 29(673) | 5.2 | 0.76 |
| Toluene | 3.22$\text{×}$10^5^ | 644 (15528) | 664 | 1.88 | 664(15060) | 20(468) | 4.8 | 0.76 |
| THF | 3.20$\text{×}$10^5^ | 646 (15480) | 669 | 1.85 | 667(14948) | 23(532) | 5.0 | 0.73 |

The optical band gap was calculated from$\text{E}_{\text{g}}^{\text{opt}}$= 1240/λ_onset_.

**Table S7.** Photophysical parameters of **SQ*n*** (*n* = 14, 16) in different organic solvents.

| *τ*_F_ (Ф_F_) | MCH | DCM | Toluene | THF |
| --- | --- | --- | --- | --- |
| **SQ14** | 5.3ns  (0.59) | 5.1ns  (0.78) | 5.0 ns  (0.83) | 5.1 ns  (0.79) |
| **SQ16** | 5.2ns  (0.57) | 5.1ns  (0.81) | 5.3 ns  (0.79) | 5.2 ns  (0.77) |


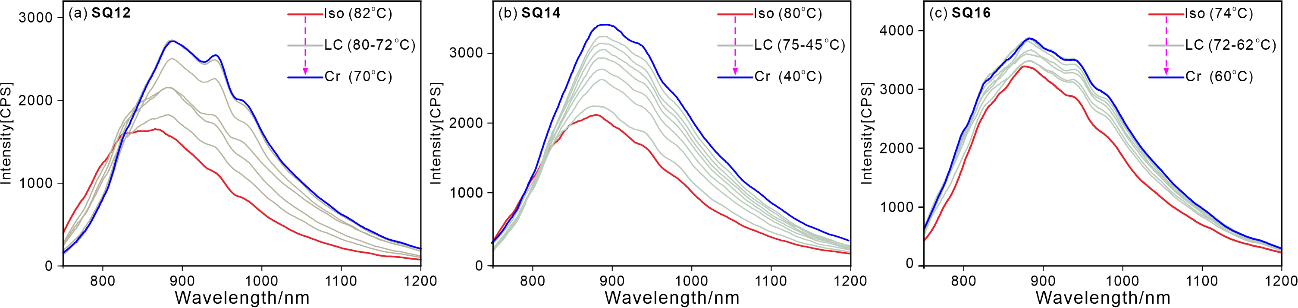


**Figure S36.** Temperature-dependent PL curves of SQ thin films on cooling (a) **SQ12**; (b) **SQ14**; (c) **SQ16**(*λ*_ex_ = 514 nm), the curves for different states are colored differently for clarity.

Firstly, the observed emission band is weak and broad, and thus we are limited to identifying individual emission channels. Besides, radiative and non-radiative channels in thermotropic LC systems could not be precisely measured due to multiple factors, including aggregate polydispersity, energetic/structural disorders, temperature, environmental polarity and various molecular interactions. Additionally, the fluorescence of H-aggregates of SQs has been reported.^S5^ Weak emissions can be recorded at around 25 K and vanish as the temperature rises. In our case (up to 353 K), the emission should be thus undetectable. To the best of our knowledge, there is no other literature reporting the emission of H-aggregates at any temperature in a bulk system. On the other hand, strongly emissive optical monomers featuring a red-shifted emission band have been found in the solid state of SQs and other dye systems.^S6-9^ Therefore, we prefer to assign the fluorescence of thin film to strongly emissive optical monomers rather than aggregates.

## 7.2 Additional temperature-dependent absorption spectra


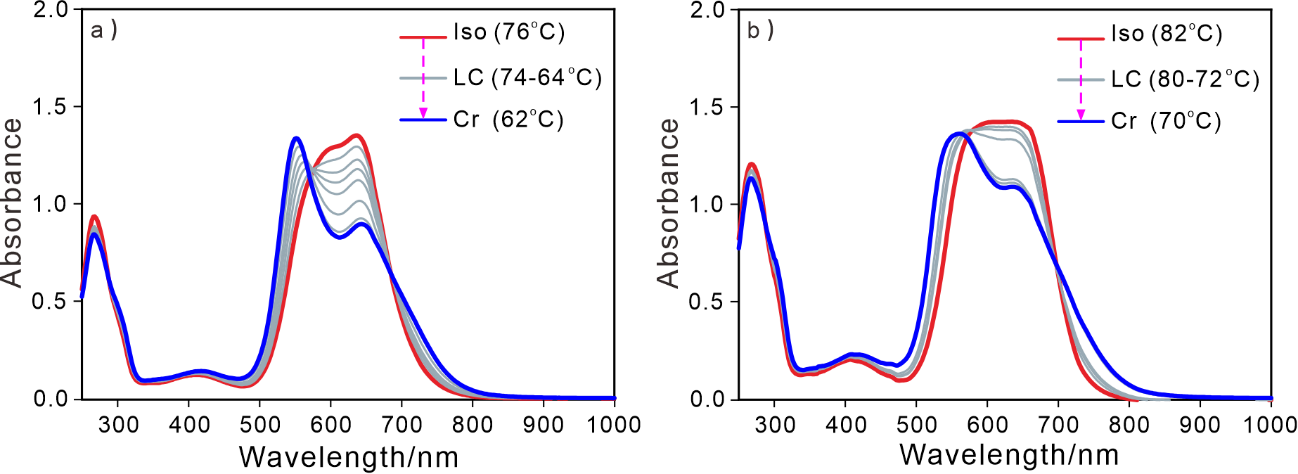


**Figure S37.** Temperature-dependent UV/vis curves on cooling (a) **SQ8/16**; (b) **SQ10/14**.

**
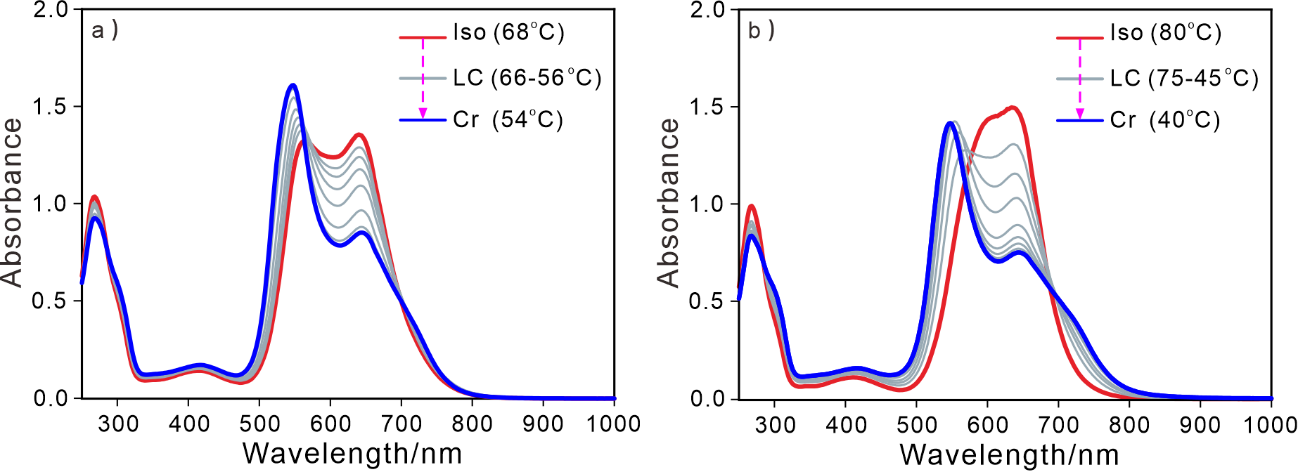
**

**Figure S38.** Temperature-dependent UV/vis curves on cooling (a) **SQ10/18**; (b) **SQ12/16**.

**
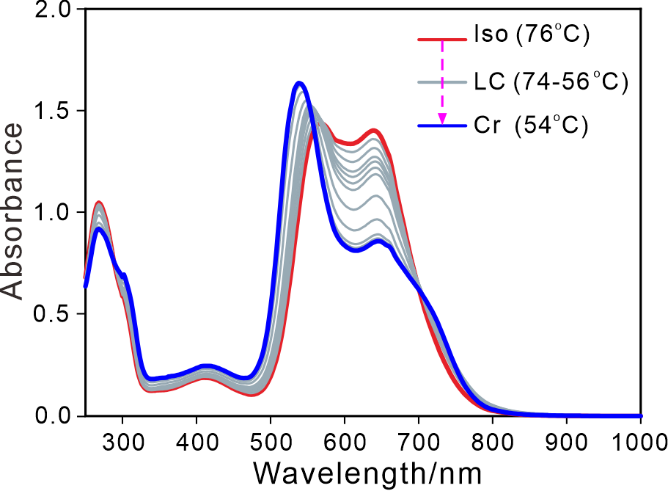
**

**Figure S39.** Temperature-dependent UV/vis curves of **SQ14/18** on cooling.

**
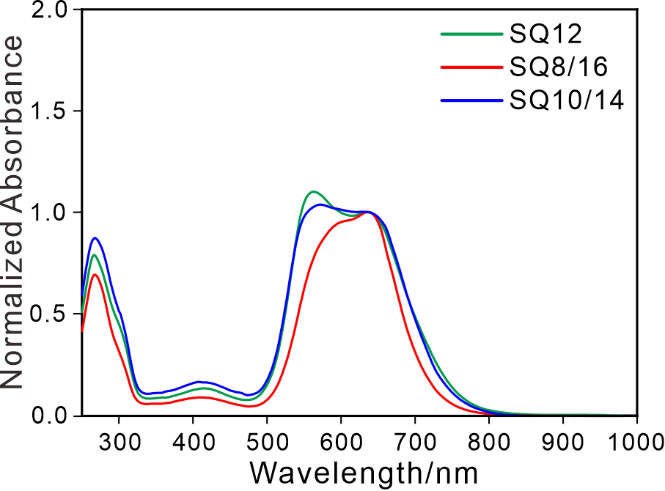
**

**Figure S40.** Normalized UV/vis curves of **SQ8/16**, **SQ10/14** and **SQ12** at 76 ℃; the absorption spectra were normalized by absorbance at 640 nm.


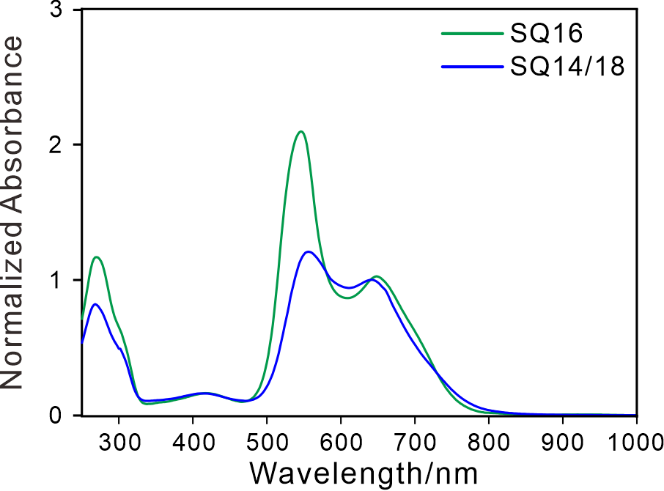


**Figure S41.** Normalized UV/vis curves of **SQ14/18** and **SQ16** at 68 ℃; the absorption spectra were normalized by absorbance at 640 nm.

## 7.3 Measurement of the dichroic ratio of thin film

The dichroic ratio was measured by the parallel and perpendicular absorbance of well-aligned **SQ14** thin film in LC state under polarized light. The quality of orientation is monitored under POM by rotating samples, see **Figure S42**b-d. Arrows with colors show the direction of shearing, and further the orientation of columns. In **Figure S42** b-d, columns are well-aligned parallel to the substrate, and dichroic ratio, *R* = 1.7, was calculated according to the following equation:

$R=\frac{A_{\parallel}}{A_{\perp}}$ Eqn. 6


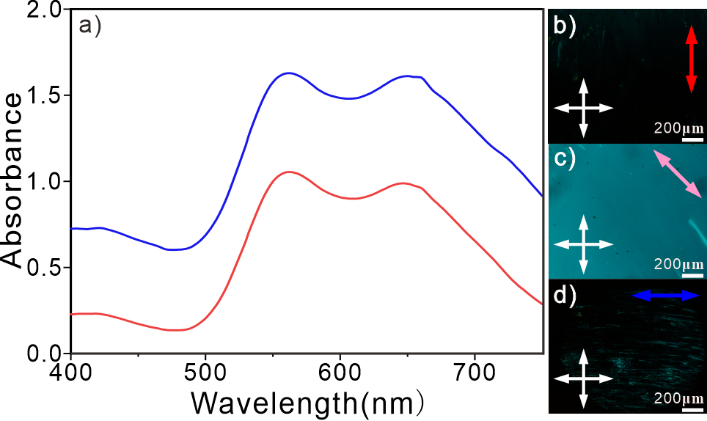


**Figure S42.** (a) Parallel (0°, blue line) and perpendicular (90°, red line) absorption curves; (b, c, d) cross-polarized optical micrographs after shearing, the pink/blue/red arrows represent shearing direction.


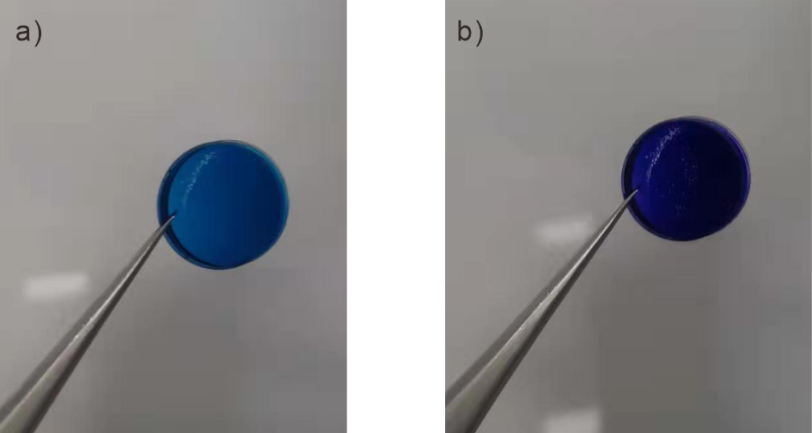


**Figure S43.** Thermochromatic SQ LC thin film of **SQ12** at different temperatures (a) blue at 80 ℃; (b) purple at 72 ℃.

# 8. SCFT Model and Method

## 8.1 SCFT Model

As **Figure** **S44** shows, we simplify each LC SQ to a polymer consisting of 12 flexible end blocks A (red) and one rigid block R (blue). Consider an incompressible melt of LC binary copolymers with thirteen blocks and a degree of polymerization *N* confined in a two-dimensional system with a volume *V*. The chain length of $\text{α}$ block is $\text{f}_{\text{α}}\text{N}\text{,}\text{ }\text{α}\text{ }\text{=}\text{ }\text{\{}\text{A}_{\text{11}}\text{,}\text{ }\text{A}_{\text{12}}\text{,}\text{ }\text{A}_{\text{13}}\text{,}{\text{ }\text{A}}_{\text{14}}\text{,}{\text{ }\text{A}}_{\text{15}}\text{,}\text{ }\text{A}_{\text{16}}\text{,}\text{ }\text{R,}\text{ }\text{A}_{\text{21}}\text{,}\text{ }\text{A}_{\text{22}}\text{,}\text{ }\text{A}_{\text{23}}\text{,}{\text{ }\text{A}}_{\text{24}}\text{,}{\text{ }\text{A}}_{\text{25}}\text{,}\text{ }\text{A}_{\text{26}}\text{\}}$ and $\text{Σ}_{\text{α}}\text{f}_{\text{α}}\text{=1}$. The statistical segment lengths of monomer A and R are $\text{b}_{\text{A}}$ and $\text{b}_{\text{R}}$, respectively.


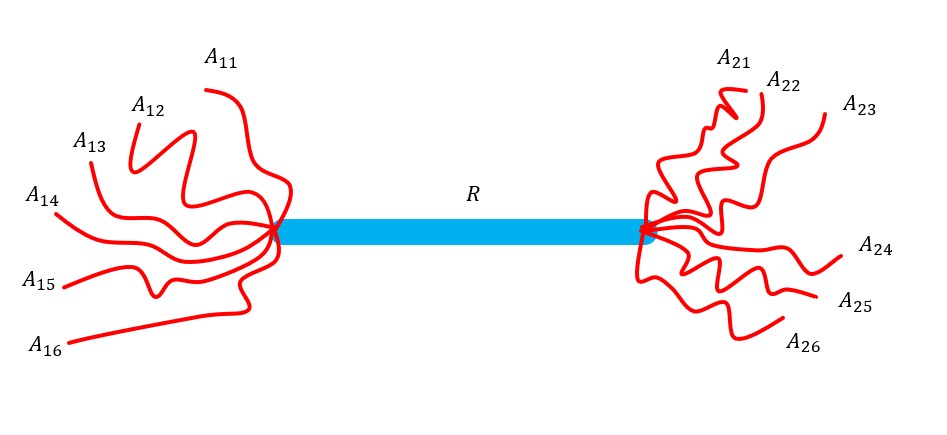


**Figure S44.** Simplified schematic of LC SQ polymer, where *R* is a rigid rod in blue, and the others are flexible chains in red.

We employ the continuous Gaussian chain model to describe flexible subchains, while the wormlike chain model to describe semi-flexible subchains. These block copolymers are represented by continuous space curves $\text{C}^{\text{i}}\left( \text{s} \right)\text{,}\text{ }\text{i}\text{ }\text{=}\text{ }\text{1,}\text{ }\text{2,}\text{⋯}\text{,}\text{ }\text{n}$, where *s*$\text{∈}\left[ \text{0,1} \right]$ is a normalized arc length variable measured along the chain contour. For the *i*-th polymer chain,$\text{C}_{\text{α}}^{\text{i}}\left( \text{s} \right)\left( \text{s}\text{ }\text{∈}\text{ }\text{I}_{\text{α}} \right)$ denotes the blocks whose monomers belong to the same species $\text{α}\text{,}\text{ }\text{α}\text{ }\text{=}\text{ }\text{A,}\text{ }\text{R}$, and $I_{\alpha}$ is the interval of corresponding contour parameter. Denote $\text{I}_{\text{A}}\text{=}\text{I}_{\text{A}_{\text{11}}}\text{∪}\text{I}_{\text{A}_{\text{12}}}\text{∪}\text{I}_{\text{A}_{\text{13}}}\text{∪}\text{I}_{\text{A}_{\text{14}}}\text{∪}\text{I}_{\text{A}_{\text{15}}}\text{∪}\text{I}_{\text{A}_{\text{16}}}\text{∪}\text{I}_{\text{A}_{\text{21}}}\text{∪}\text{I}_{\text{A}_{\text{22}}}\text{∪}\text{I}_{\text{A}_{\text{23}}}\text{∪}\text{I}_{\text{A}_{\text{24}}}\text{∪}\text{I}_{\text{A}_{\text{25}}}\text{∪}\text{I}_{\text{A}_{\text{2}\text{6}}}$,

$\text{I}_{\text{R}}\text{=[}\text{f}_{\text{A}_{\text{11}}}\text{,}\text{f}_{\text{A}_{\text{11}}}\text{+}\text{f}_{\text{R}}\text{]}$,$\int_{\text{I}_{\text{A}}} \text{ }\text{=}\int_{\text{0}}^{\text{f}_{\text{A}_{\text{11}}}} \text{ +}\int_{\text{0}}^{\text{f}_{\text{A}_{\text{12}}}} \text{ +}\int_{\text{0}}^{\text{f}_{\text{A}_{\text{13}}}} \text{ +}\int_{\text{0}}^{\text{f}_{\text{A}_{\text{14}}}} \text{ +}\int_{\text{0}}^{\text{f}_{\text{A}_{\text{15}}}} \text{ +}\int_{\text{0}}^{\text{f}_{\text{A}_{\text{16}}}} \text{ +}\int_{\text{0}}^{\text{f}_{\text{A}_{\text{21}}}} \text{ +}\int_{\text{0}}^{\text{f}_{\text{A}_{\text{22}}}} \text{ +}\int_{\text{0}}^{\text{f}_{\text{A}_{\text{23}}}} \text{ +}\int_{\text{0}}^{\text{f}_{\text{A}_{\text{24}}}} \text{ +}\int_{\text{0}}^{\text{f}_{\text{A}_{\text{25}}}} \text{ +}\int_{\text{0}}^{\text{f}_{\text{A}_{\text{26}}}} \text{ }$, $\int_{\text{I}_{\text{R}}} \text{ }\text{=}\text{ }{\int_{\text{f}_{\text{A}_{\text{11}}}}^{\text{f}_{\text{A}_{\text{11}}}\text{+}\text{f}_{\text{R}}} \text{ }}_{\text{ }}$.

The normalized microscopic monomer density of the type-$\text{α}$ species at space position ***r*** is

| $\begin{matrix} \hat{\phi}_{A}\left( \boldsymbol{r} \right)=\frac{N}{\phi_{0}}\sum_{i=1}^{n} \int_{I_{A}} \delta\left[ \boldsymbol{r}-C_{A}^{i}\left( s \right) \right]ds, \\ \hat{\phi}_{R}\left( \boldsymbol{r} \right)=\frac{N}{\phi_{0}}\sum_{i=1}^{n} \int_{I_{R}} \delta\{\boldsymbol{r}-\left[ C_{R}^{i}\left( s \right)+b_{R}u^{i}(s) \right]\}ds, \end{matrix}$ | (Eqn.7) |
| --- | --- |

where $\text{ϕ}_{\text{0}}$= *nN*/*V* is average monomer density, ***u***(*s*) = d***r***(*s*)/ds is a vector defined on the unit sphere $\mathcal{S}$, which represents the local orientation of the semiflexible molecule at contour location *s*. The incompressibility constraint is provided by enforcing that

| $\sum_{\alpha} \hat{\phi}_{\alpha}=1,\alpha=A,R.$ | (Eqn.8) |
| --- | --- |

The orientational order parameter is

| $\hat{\boldsymbol{S}}\left( \boldsymbol{r} \right)=\frac{N}{\phi_{0}}\sum_{i=1}^{n} \int_{I_{R}} \delta\{\boldsymbol{r}-\left[ C_{R}^{i}\left( s \right)+b_{R}\boldsymbol{u}^{i}(s) \right]\}(\boldsymbol{u}^{i}\boldsymbol{u}^{i}-\frac{\boldsymbol{I}}{2})ds.$ | (Eqn.9) |
| --- | --- |

The conformations of noninteracting flexible components are ascribed a Gaussian statistical weight, $\text{exp(}\text{F}_{\text{0}}\text{)}$, with a harmonic stretching (free) energy given by (units of $\text{k}_{\text{B}}\text{T}$)

| $F_{0}=\frac{3}{2{b_{A}}^{2}}\sum_{i=1}^{n} \int_{I_{A}} \left\vert\frac{d C_{A}^{i}\left( s \right)}{ds} \right\vert^{2}ds.$ | (Eqn.10) |
| --- | --- |

The conformations of noninteracting semiflexible components are ascribed a wormlike statistical weight, $\text{exp(}\text{F}_{\text{1}}\text{)}$, with a bending (free) energy given by

| $F_{1}=\frac{\lambda}{2{b_{R}}^{2}}\sum_{i=1}^{n} \int_{I_{R}} \left\vert\frac{d\boldsymbol{u}^{\boldsymbol{i}}\left( s \right)}{ds} \right\vert^{2}ds,$ | (Eqn.11) |
| --- | --- |

where $\text{λ}$is the persistence length, i.e. the distance along the contour of a semi-flexible chain over which orientational correlations decay.

Interactions between monomers on the same or different polymers are usually modeled by pseudopotentials in the monomer densities, e.g., a local quadratic form in the microscopic monomer densities. In the case of the two different species melt considered here, we adopt the following form for this pseudopotential

| $F_{I}=\phi_{0}\int_{V} \chi_{AR}\hat{\phi}_{A}\left( \boldsymbol{r} \right)\hat{\phi}_{R}\left( \boldsymbol{r} \right)d\boldsymbol{r}$ | (Eqn.12) |
| --- | --- |

where $\text{χ}_{\text{AR}}$ represents the Flory-Huggins interaction parameter between the A and R blocks. The Maier-Saupe-type orientational interaction is adopted to describe the excluded volume interactions between the semi-flexible subchains

| $F_{M}=-\frac{\eta\phi_{0}}{2}\int_{V} \hat{\boldsymbol{S}}\left( \boldsymbol{r} \right):\hat{\boldsymbol{S}}\left( \boldsymbol{r} \right)d\boldsymbol{r},$ | (Eqn.13) |
| --- | --- |

where $\text{η}$represents the strength of the orientational interaction favoring the alignment of the semi-flexible subchains. The symbol $\text{A:B}$ denotes the double dot product, defined as $\text{A:B=}\sum_{\text{ij}} \text{A}_{\text{ij}}\text{B}_{\text{ij}}\text{.}$ The particle-based partition function is

| $Z=\int_{\mathcal{S}} \int_{\mathbb{R}^{3}} \delta\left[ \sum_{\alpha=A,R} \hat{\phi}_{\alpha}\left( \boldsymbol{r} \right)-1 \right]\exp\left[ -F_{0}-F_{1}-F_{I}-F_{M} \right]\}D\boldsymbol{u}^{\boldsymbol{i}}\left( s \right)DC^{i}\left( s \right).$ | (Eqn.14) |
| --- | --- |

where the delta functional constraint imposes the local incompressibility condition. A typical starting point is to decouple the quadratic density and orientational interactions through a Hubbard-Stratanovich transformation, leading to a representation wherein independent polymer chains are coupled to fluctuating potential fields. By this approach, the derived field-based theory has good mathematical feature that shows the equilibrium states are saddle-points. Moreover, the descent and ascent directions of saddle-points of the effective field-based energy have been shown explicitly. Thus, the field-based partition function is

| $Z=\int_{\mathbb{R}^{3}} \int_{\mathbb{R}^{3}} \int_{\mathbb{R}^{3}} \exp\{-nF\left[ \mu_{+},\mu_{-},\boldsymbol{M} \right]\}D\mu_{+}\left( \boldsymbol{r} \right)D\mu_{-}\left( \boldsymbol{r} \right)D\boldsymbol{M}\left( \boldsymbol{r} \right).$ | (Eqn.15) |  |
| --- | --- | --- |

where $\text{F}$is the free energy of LC SQ polymer melt,

| $\frac{F}{nk_{B}T}=\frac{1}{V}\int_{V} \left[ -\mu_{+}\left( \boldsymbol{r} \right)+\frac{1}{\chi_{AR}\phi_{0}N}\mu_{-}^{2}\left( \boldsymbol{r} \right) \right]d\boldsymbol{r}-\frac{1}{2\eta N}\int_{V} \boldsymbol{M}\left( \boldsymbol{r} \right):\boldsymbol{M}\left( \boldsymbol{r} \right)d\boldsymbol{r}-log Q.$ | (Eqn.16) |
| --- | --- |

where $\text{Q}$is the single chain partition function. $\mu_{-}$is the general ‘exchange chemical potential’ of the system. $\text{µ}_{\text{+}}$ is the pressure potential to ensure the local incompressibility. ***M*** is the orientational field of semi-flexible chain. Mean fields $\text{ω}_{\text{α}}$, $\text{α}\text{ }\text{=}\text{ }\text{\{}\text{A, R}\text{\}}$ produced by the surrounding chains, satisfy

| $\begin{matrix} \omega_{A}=\mu_{+}-\mu_{-}, \\ \omega_{R}=\mu_{+}+\mu_{-}. \end{matrix}$ |  |
| --- | --- |

The single-chain partition function *Q*, the monomer density $\text{ϕ}_{\text{α}}$,$\text{α}\text{ }\text{=}\text{ }\text{\{A, R\}}$ and order-parameter $\text{S}$ can be computed through solving a set of modified diffusion equation of chain propagators $\text{q}_{\text{β}}\text{ }\text{(}\text{r}\text{,}\text{ }\text{s}\text{)}$ and $\text{q}_{\text{β}}^{\text{†}}\text{ }\text{(}\text{r}\text{,}\text{ }\text{s}\text{)}$, $\text{β}\text{ }\text{=}\text{ }\text{\{}\text{A}_{\text{11}}\text{,}{\text{ }\text{A}}_{\text{12}}\text{,}{\text{ }\text{A}}_{\text{13}}\text{,}\text{ }\text{A}_{\text{14}}\text{,}\text{ }\text{A}_{\text{15}}{\text{,}\text{ }\text{A}}_{\text{16}}\text{,}\text{ }\text{A}_{\text{21}}\text{,}{\text{ }\text{A}}_{\text{22}}\text{,}\text{ }\text{A}_{\text{23}}\text{,}\text{ }\text{A}_{\text{24}}\text{,}\text{ }\text{A}_{\text{25}}\text{,}\text{ }\text{A}_{\text{26}}\text{\}}$, $\text{q}_{\text{R}}\text{(}\text{r}\text{,}\text{ }\text{u}\text{,}\text{ }\text{s}\text{)}$ and $\text{q}_{\text{R}}^{\text{†}}\text{(}\text{r}\text{,}\text{ }\text{u}\text{,}\text{ }\text{s}\text{)}$.

| $\begin{matrix} Q=\frac{1}{V}\int_{V} q_{A_{11}}\left( \boldsymbol{r},s \right)q_{A_{11}}^{\dagger}\left( \boldsymbol{r},s \right)d\boldsymbol{r}, s\in I_{A_{11}}, \\ \phi_{A}\left( \boldsymbol{r} \right)=\frac{1}{Q}\sum_{\beta} \int_{0}^{f_{\beta}} q_{\beta}\left( \boldsymbol{r},s \right)q_{\beta}^{\dagger}\left( \boldsymbol{r},s \right)ds, \\ \phi_{R}\left( \boldsymbol{r} \right)=\frac{2\pi}{Q}\int_{I_{R}} \int_{\mathcal{S}} q_{R}\left( \boldsymbol{r},\boldsymbol{u},s \right)q_{R}^{\dagger}\left( \boldsymbol{r},\boldsymbol{u},s \right)d\boldsymbol{u} ds, \\ S\left( \boldsymbol{r} \right)=\frac{2\pi}{Q}\int_{I_{R}} \int_{\mathcal{S}} q_{R}\left( \boldsymbol{r},\boldsymbol{u},s \right)\left( \boldsymbol{uu}-\frac{I}{2} \right)q_{R}^{\dagger}\left( \boldsymbol{r},\boldsymbol{u},s \right)d\boldsymbol{u}ds. \end{matrix}$ | (Eqn.17) |
| --- | --- |

$\text{q}_{\text{β}}\text{(}\text{r}\text{, }\text{s}\text{)}$ is the forward propagator, representing the probability of finding the *s*-th $\text{β}$segment at a spatial position$\text{r}$ from *s* = 0 to $\text{s}\text{ }\text{=}\text{ }\text{f}_{\text{β}}$under mean field $\text{ω}_{\text{A}}$. The backward propagator $\text{q}_{\text{β}}^{\text{†}}\text{(}\text{r}\text{,}\text{ }\text{s}\text{)}$ represents the probability weight from $\text{s}\text{ }\text{=}{\text{ }\text{f}}_{\text{β}}$to *s* = 0. Denote $\text{A}_{\text{1}}\text{ }\text{=}\text{ }\left\{ \text{A}_{\text{11}}\text{,}{\text{ }\text{A}}_{\text{12}}\text{,}\text{ }\text{A}_{\text{13}}\text{,}{\text{ }\text{A}}_{\text{14}}\text{,}{\text{ }\text{A}}_{\text{15}}\text{,}{\text{ }\text{A}}_{\text{16}} \right\}$,$\text{A}_{\text{2}}\text{ }\text{=}\text{ }\text{β}\backslash\text{A}_{\text{1}}$.From the continuous Gaussian chain model, they satisfy the modified diffusion equations，

| $\begin{matrix} \frac{\partial q_{\beta}\left( \boldsymbol{r},s \right)}{\partial s}={\frac{{b_{A}}^{2}}{6}\nabla}_{\boldsymbol{r}}^{2}q_{\beta}\left( \boldsymbol{r},s \right)-\omega_{A}\left( \boldsymbol{r} \right)q_{\beta}\left( \boldsymbol{r},s \right), s\in{I_{\beta},} \\ q_{\beta}\left( \boldsymbol{r},0 \right)=1. \end{matrix}$ | (Eqn.18) |
| --- | --- |
| $\begin{matrix} \frac{\partial q_{\beta}^{\dagger}\left( \boldsymbol{r},s \right)}{\partial s}=\frac{{b_{A}}^{2}}{6}\nabla_{\boldsymbol{r}}^{2}q_{\beta}^{\dagger}\left( \boldsymbol{r},s \right)-\omega_{A}\left( \boldsymbol{r} \right)q_{\beta}^{\dagger}\left( \boldsymbol{r},s \right), s\in I_{\beta}, \\ q_{i}^{\dagger}\left( \boldsymbol{r},0 \right)=\prod_{A_{1}\backslash\{i\}} q_{i}\left( \boldsymbol{r},f_{i} \right)\int_{\mathcal{S}} q_{R}^{\dagger}\left( \boldsymbol{r},\boldsymbol{u},f_{R} \right)d\boldsymbol{u}, i\in A_{1}, \\ q_{j}^{\dagger}\left( \boldsymbol{r},0 \right)=\prod_{A_{2}\backslash\{j\}} q_{j}\left( \boldsymbol{r},f_{j} \right)\int_{\mathcal{S}} q_{R}^{\dagger}\left( \boldsymbol{r},\boldsymbol{u},f_{R} \right)d\boldsymbol{u}, j\in\text{A}_{\text{2}}. \\ \end{matrix}$ | (Eqn.19) |

The forward propagators of semi-flexible chains $\text{q}_{\text{R}}\text{(}\text{r}\text{,}\text{ }\text{u}\text{,}\text{ }\text{s)}$ represent the probability that the endpoint of the *s* segment at the spatial position $\text{r}$ and orientational position$\text{u}$. $\text{u}$is a vector defined on the unit sphere, which represents the local orientation of the semi-flexible molecule. They satisfy the “convective diffusion” equations

| $\begin{matrix} \frac{\partial}{\partial s}q_{R}\left( \boldsymbol{r},\boldsymbol{u},s \right)=(-\beta\cdot\nabla_{\boldsymbol{r}}-\Gamma\left( \boldsymbol{r},\boldsymbol{u} \right)+\frac{1}{2\lambda}\nabla_{\boldsymbol{u}}^{2}{) q}_{R}\left( \boldsymbol{r},\boldsymbol{u},s \right), \\ q_{R}\left( \boldsymbol{r},\boldsymbol{u},0 \right)=\frac{1}{2\pi}\prod_{{i\in A}_{1}} q_{i}\left( \boldsymbol{r},f_{i} \right), s\in{I_{R}}. \end{matrix}$ | (Eqn.20) |
| --- | --- |

Where $\text{Γ}\text{(}\text{r}\text{, }\text{u}\text{)}\text{ = }\text{ω}_{\text{R}}\text{(}\text{r}\text{)}\text{-}\text{M}\text{(}\text{r}\text{, }\text{u}\text{)}\text{:[}\text{uu}\text{-}\frac{\text{I}}{\text{2}}\text{]}$.Parameter $\text{β}\text{ }\text{=}\text{ }$($\text{b}_{\text{R}}\text{/}\text{b}_{\text{A}}$)${\text{(6}\text{N}\text{)}}^{\text{1/2}}$ measures the size asymmetry of monomer R and A. $\text{ }\text{λ}$is the hardness of the semi-flexible chain. The backward propagators of the semi-flexible block can be expressed as

| $\begin{matrix} \frac{\partial}{\partial s}q_{R}^{\dagger}\left( \boldsymbol{r},\boldsymbol{u},s \right)=(\beta\cdot\nabla_{\boldsymbol{r}}-\Gamma\left( \boldsymbol{r},\boldsymbol{u} \right)+\frac{1}{2\lambda}\nabla_{\boldsymbol{u}}^{2}{) q}_{R}^{\dagger}\left( \boldsymbol{r},\boldsymbol{u},s \right), \\ {q_{R}}^{\dagger}\left( \boldsymbol{r},\boldsymbol{u},0 \right)=\frac{1}{2\pi}\prod_{{j\in A}_{2}} q_{j}\left( \boldsymbol{r},f_{j} \right), s\in{I_{R.}} \end{matrix}$ | (Eqn.21) |
| --- | --- |

The first-order variations of the free energy with respect to field functions yield the SCFT equations.

| $\begin{matrix} \phi_{A}\left( \boldsymbol{r} \right)+\phi_{R}\left( \boldsymbol{r} \right)-1=0, \\ \frac{2}{\chi_{AR}N}\mu_{-}\left( \boldsymbol{r} \right)-\phi_{A}\left( \boldsymbol{r} \right)+\phi_{R}\left( \boldsymbol{r} \right)=0, \\ \frac{1}{\eta N}\boldsymbol{M}\left( \boldsymbol{r} \right)-\boldsymbol{S}\left( \boldsymbol{r} \right)=0. \end{matrix}$ | (Eqn.22) |
| --- | --- |

In the LC SQ system, there are five independent model parameters, including one composition parameter,$\text{f}_{\text{R}}$,(assume all the A chains have the same length), two interaction parameters,${\text{ }\text{χ}}_{\text{AR}}$, $\text{η}$, one parameter $\text{β}$ measuring the conformational asymmetry, one parameter $\text{λ}$ describing the hardness of rod molecule.

## 8.2 Method

SCFT equations are a set of highly nonlinear equations with multi-solutions and multi-parameters. Iterative techniques are required to solve such a complicated system. The specific iteration processes for the self-consistent systems are given in the following:

1. Step 1: Give reasonable initial values of chemical potential fields, order-parameter, calculation box;
2. Step 2: Solve propagators;
3. Step 3: Obtain single partition function Q, density operators $\text{ϕ}_{\text{α}}$,$\text{α}\text{ }\text{=}\text{ \{}\text{A, R}\text{\}}$ and order-parameter *S*;
4. Step 4: Update potential fields $\text{µ}_{\text{+}}$, $\text{μ}_{\text{-}}$, order-parameter ***M*** and calculation box;
5. Step 5: If a given convergent condition is achieved, stop iteration procedure, else go to Step 2.

The binary thirteen block LC SQ polymers are confined on a two-dimensional rectangular box with sizes $\text{L}_{\text{x}}\text{×}\text{L}_{\text{y}}$. The orientational vector ***u*** is restricted to unit circle. the fourth-order backward difference formula and the fourth-order Runge-Kutta method to discrete the contour variable of Gaussian and Wormlike chain propagators, respectively^S10, S11^. And the hybrid nonlinear iteration scheme is used to find the saddle points of the SCFT ^S11^.

Through our SCFT calculations, we can describe the probability distribution ***O*** of the orientation of the rigid rods,

| $O\left( \boldsymbol{r},\boldsymbol{u} \right)=\int_{I_{R}} q_{R}\left( \boldsymbol{r},\boldsymbol{u},s \right)q_{R}^{\dagger}\left( \boldsymbol{r},\boldsymbol{u},s \right)ds.$ | (Eqn.23) |
| --- | --- |

In columnar LC system, the equilibrium state along columnar axis is homogeneous, which suggests the molecular packing along the columnar axis can be simplified as summation of strata containing only one single molecule. Besides, considering the dynamic nature of LC, the initial state of the orientation field in the stratum is supposed to be arbitrary, i.e. the general transition pathway of orientation should be independent from initial state. Two different initial states, ordered/random states, are considered in Table S8 and for all molecular models, the low energy states are qualitatively same. In this way, the density profile and orientation distribution of squaraine core region for the Col_hex_ phase(**Figure** **S45**) are used to evaluate the aggregation behavior in the columnar phase. The one dominating orientation at low energy suggests high possibility of H-aggregation.


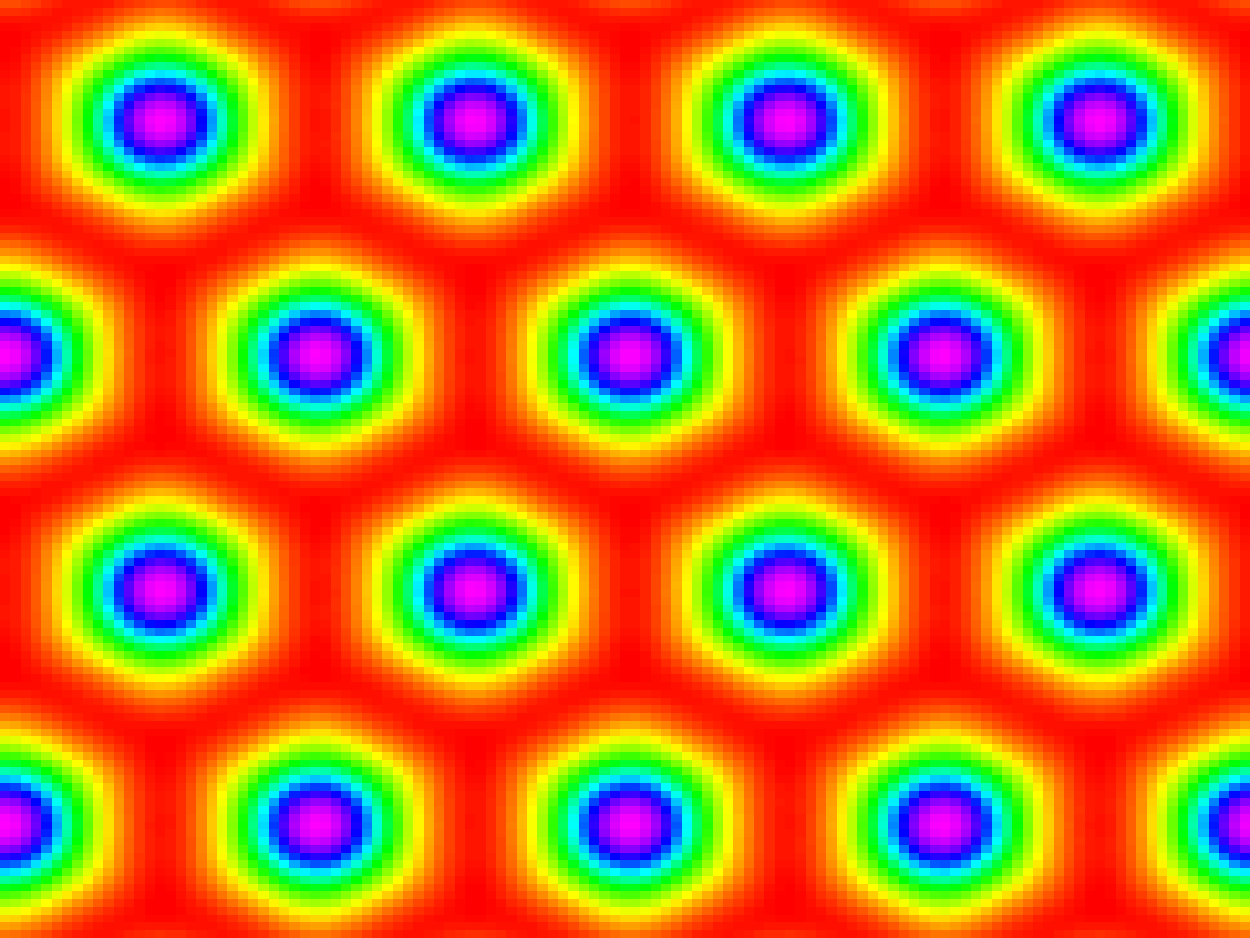

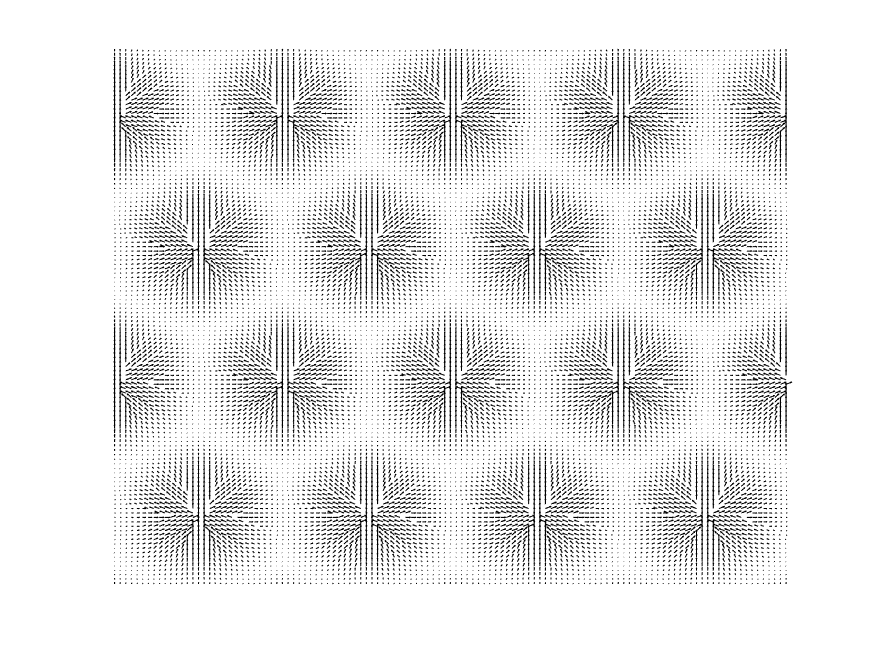


**Figure S45.** (Left) Density profile. (Right) Orientation distribution ***O*** of squaraine core region (black frame) when $\text{χ}_{\text{AR}}\text{N}\text{ }\text{=}\text{ }\text{45, }\text{β}\text{ }\text{=}\text{ }\text{6, }\text{η}\text{ }\text{=}\text{ }\text{0.15,}{\text{ }\text{f}}_{\text{R}}\text{ }\text{=}\text{ }\text{0.207,}\text{ }\text{λ}\text{ }\text{=}\text{ }\text{300,}{\text{ }\text{L}}_{\text{x}}\text{ =}\text{ }\text{6.7}\text{1}\text{, }\text{L}_{\text{y}}\text{ }\text{=}\text{ }\text{5.84}$.

**Table S8.** Orientation distribution of SQ cores for low free energy state depending on different *f*_R_ and initial orientation distribution (*O*)ofSQ cores. Note: Iter represents the iteration numbers.

| *f*_R_ | *O*_random_ | *O* = 2 |
| --- | --- | --- |
| / | 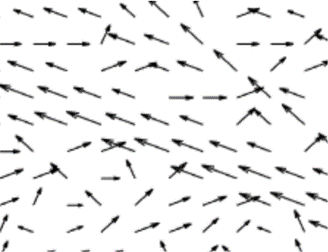  Iter=0 | 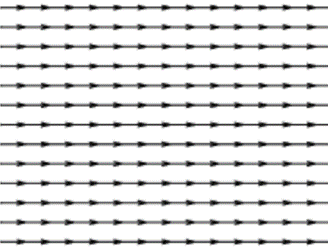  Iter=0 |
| 0.150 | 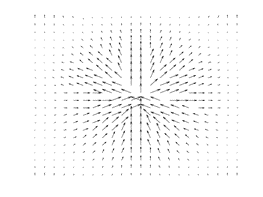  Iter=1174 | 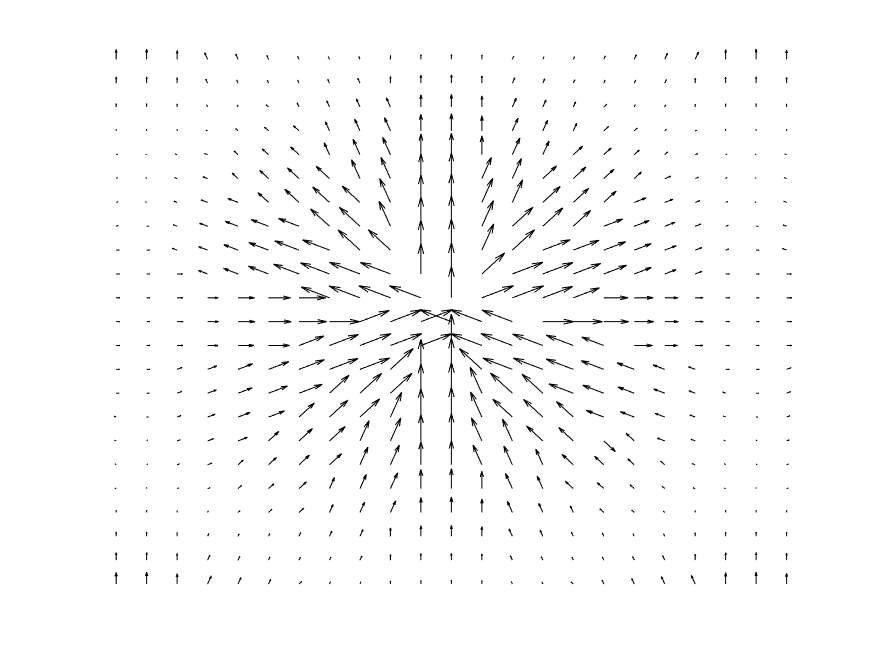  Iter=1174 |
| 0.165 | 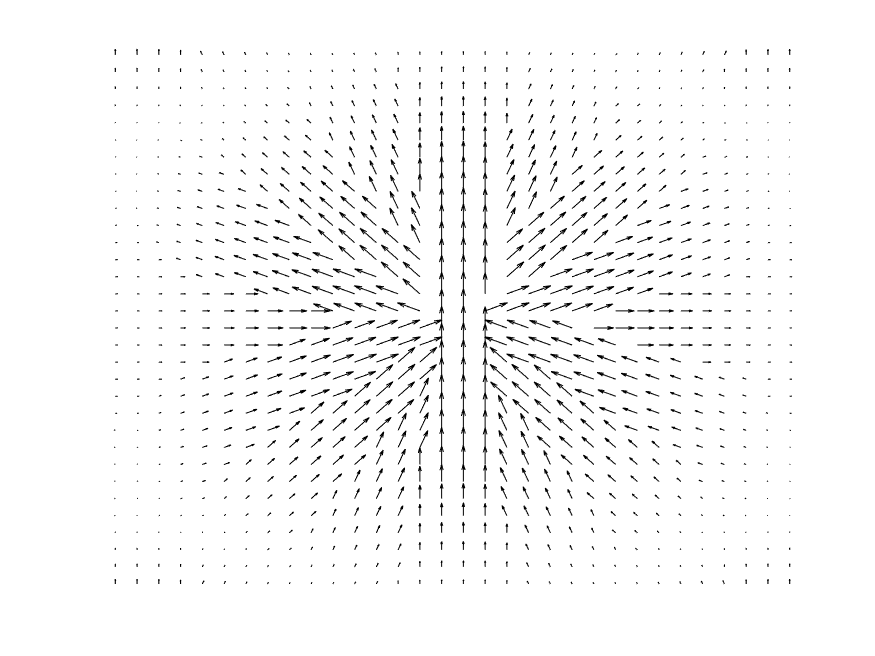  Iter=1450 | 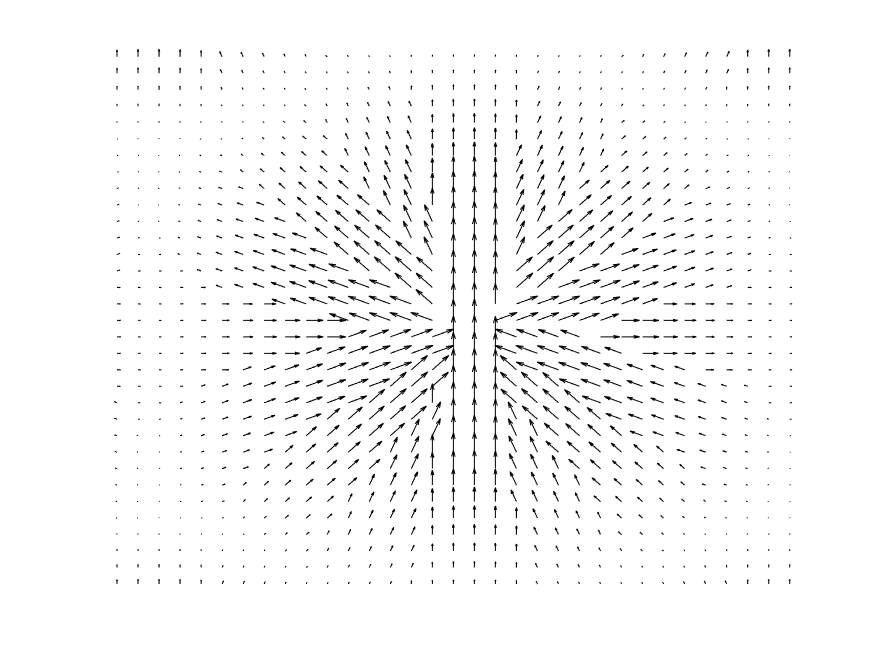  Iter=1610 |
| 0.184 | 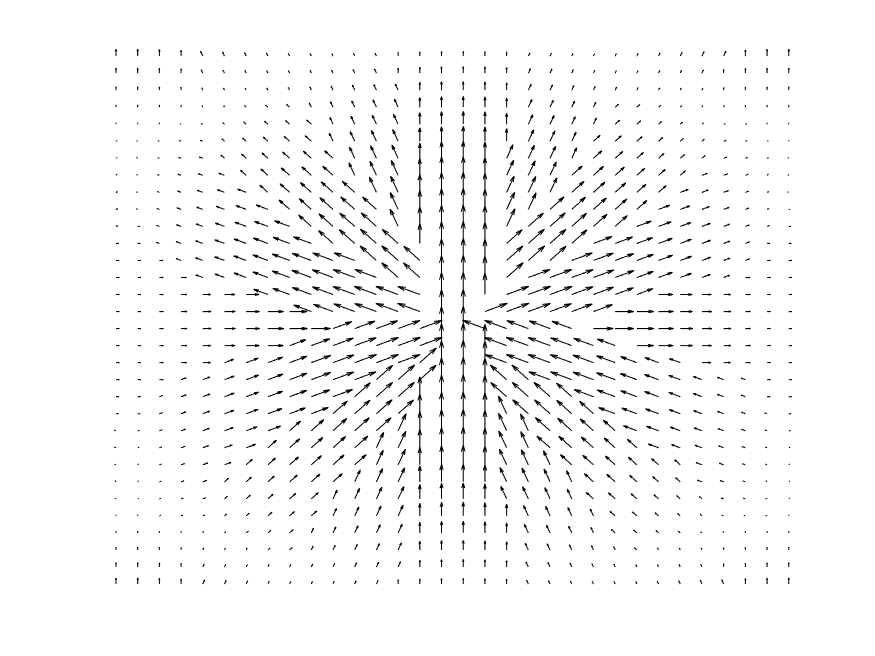  Iter=1366 | 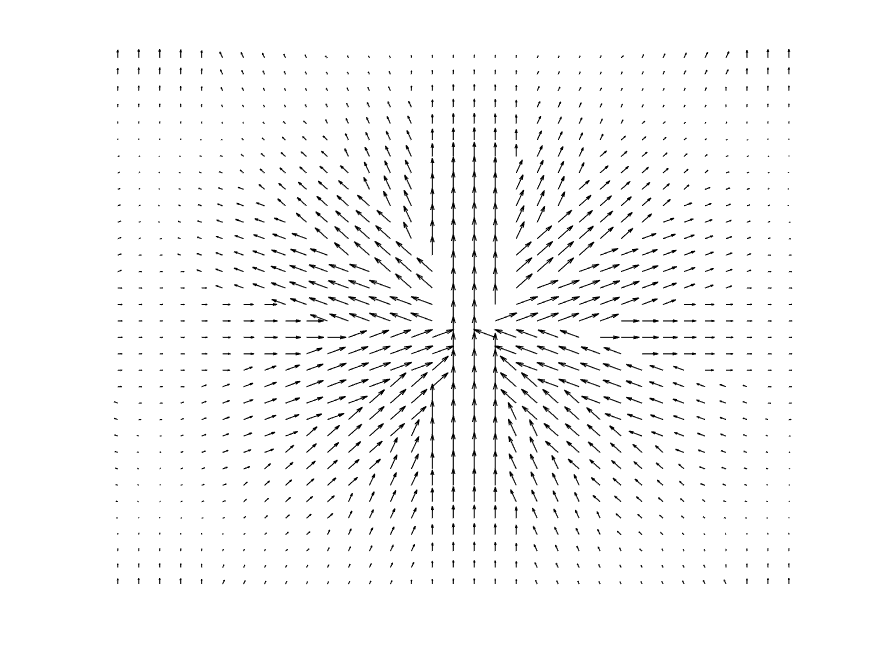  Iter=1623 |
| 0.207 | 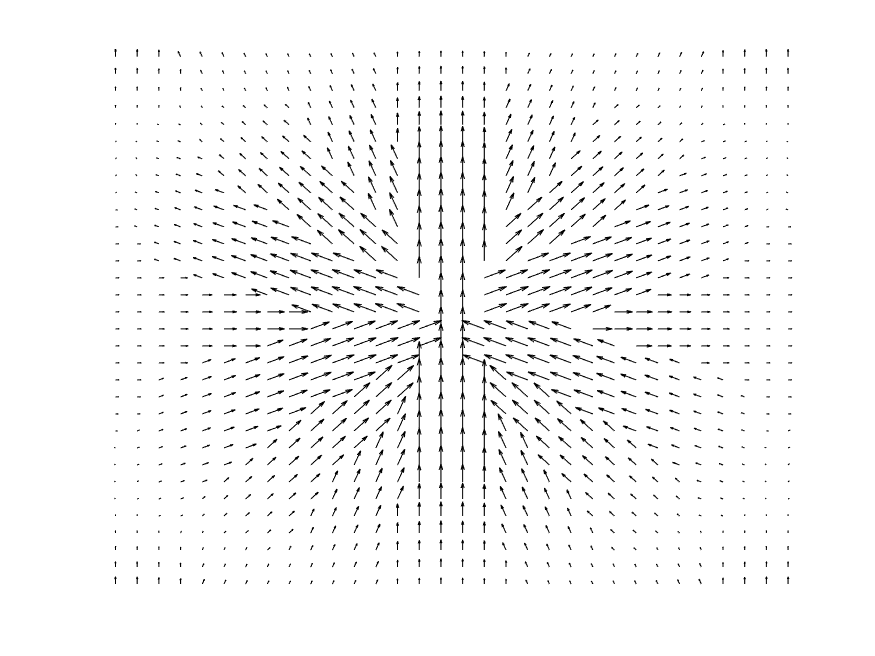  Iter=1383 | 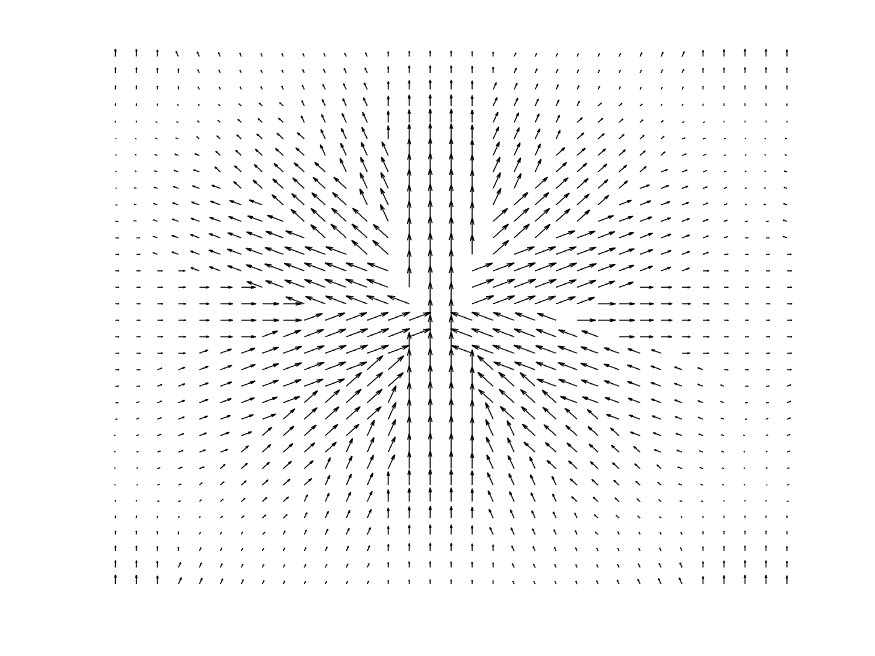  Iter=1279 |
| 0.236 | 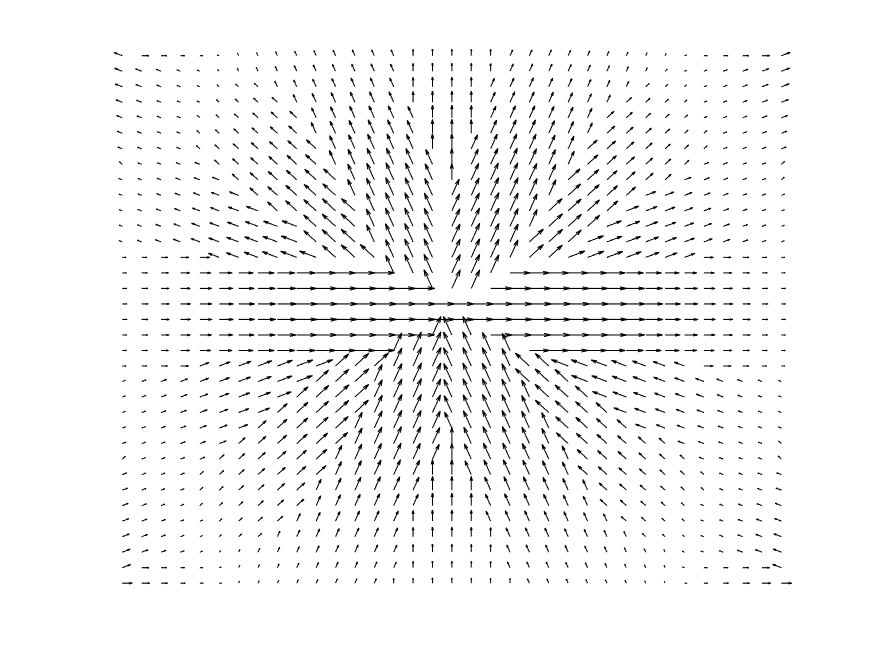  Iter=1255 | 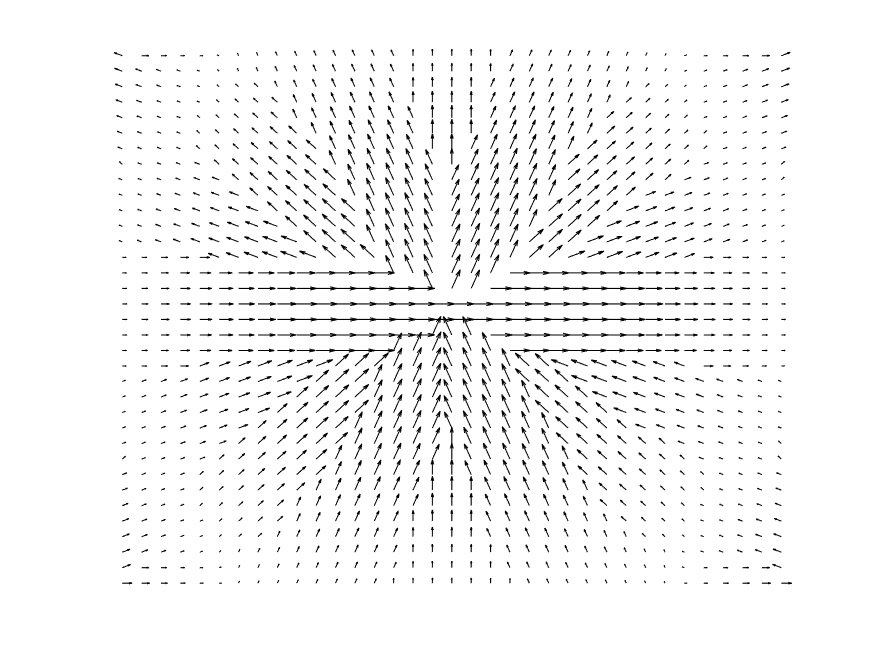  Iter=1238 |
| 0.276 | 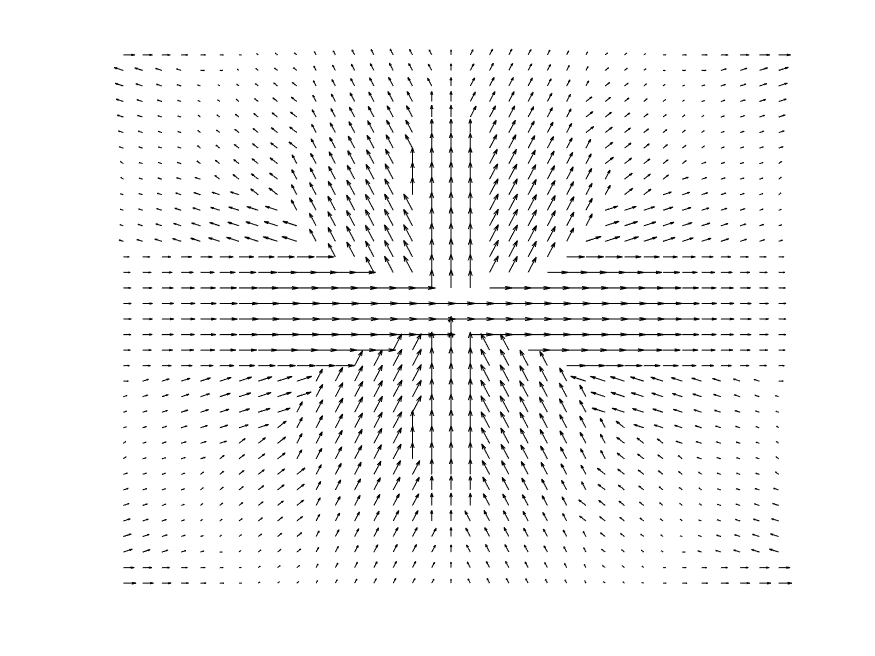  Iter=2460 | 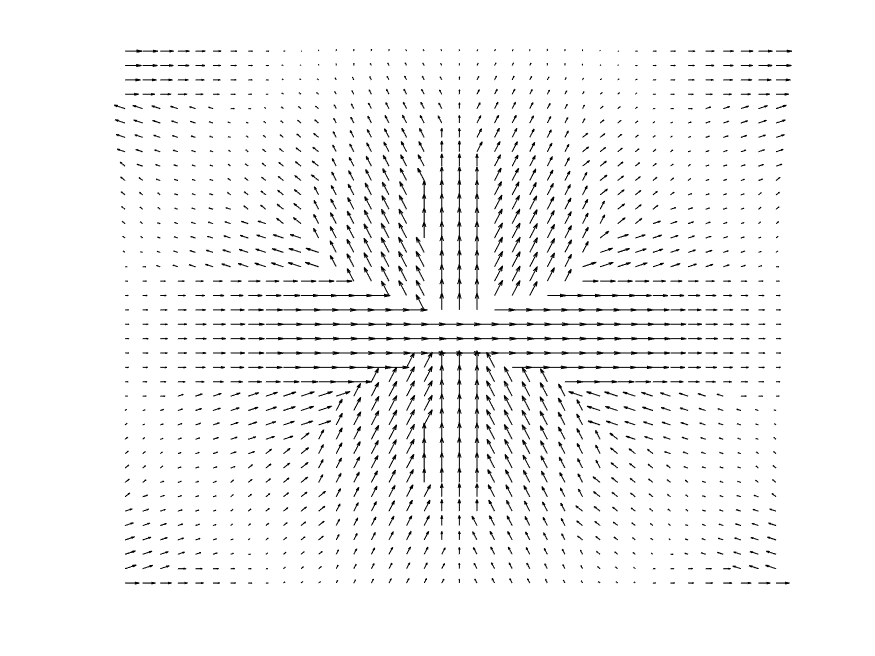  Iter=2444 |

*f*_R_ refers to the volume ratio of aromatic core.

GIF-direction contains 700 frames of *f*_R_ = 0.207 (SQ14).

GIF-density is the related change of the packing density for same compound.

# 9. Excited-State Calculations

## 9.1 Computational details

Theoretical calculations were performed to calculate the one-photon absorption spectra of SQ monomer and dimers in different configurations based on time-dependent density functional theory (TD-DFT). The molecular structures of monomer and dimer which we considered for our DFT calculations are shown in **Figure S46**. For simplicity, we terminate the N atom with two methyl groups. The ground-state geometries of SQ monomer and dimer were optimized with the Coulomb-attenuated B3LYP (CAM-B3LYP) exchange-correlation functional^S12^ together with Grimme’s D3 van der Waals correction (CAM-B3LYP+D3).^S13^ All other calculations involving excited states were based on time-dependent DFT. Solvent effects were considered by using the Polarizable Continuum Model, and a temperature effect was not included (i.e. essentially at 0 K), for both ground-state and excited-state calculations. Following the experimental UV/vis setup, we specified dichloromethane (CH_2_Cl_2_) as the solvent in all DFT calculations. Following our previous work on oligofluorenes^S14^, we used the CAM-B3LYP functional to calculate the excitation energies and transition dipoles, which was designed to better describe the long-range contributions to the electronic exchange interaction. All DFT calculations were performed using the Gaussian 16 package^S15^, and we used the 6-31+G* basis set throughout the present work. A homogeneous broadening, with a peak half-width at half height value of 0.1 eV, is included in all the spectra plotted.

## 9.2 Computational results

To understand how the geometric overlap between neighbouring SQ monomers may affect the optical properties of dimers or higher aggregates, we created a series of dimer configurations. We started from the DFT optimized ground-state structure of dimer (denoted as dimer-X+0 nm, see **Figure S47**). We then manually translate or rotate one monomer with respect to the other by different distances or angles (see example configurations in **Figure S47b, c**), and calculate their vertical one-photon absorption (OPA) spectra. We show the calculated OPA spectra in **Figure S48**, and we summarize the excitation energies and oscillator strength in **Tables S7** and **S8**.

Despite excellent qualitative agreement between the calculated one-photon absorption spectra (see **Figure S48**) and experimental UV/vis absorption spectra (see **Figure 4**), we note that our TD-DFT predicted excitation energies based on the CAM-B3LYP functional are higher than those measured by the UV/vis absorption spectra. This is a known issue, and similar overestimation of excitation energies by TD-CAM-B3LYP calculations was reported in the previous literature^S16, 17^. We would like to stress that the use of the CAM-B3LYP functional is important for accurate description of charge-transfer contributions to electronic excitations^S15^ which are present in SQ materials.


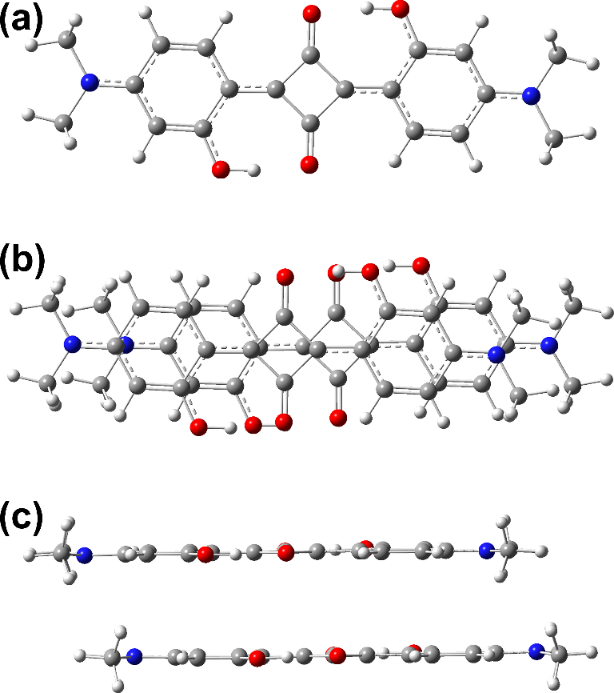


**Figure S46**. Molecular models considered in the DFT calculation: (a) monomer (top view), (b) dimer (top view) and (c) dimer (side view).


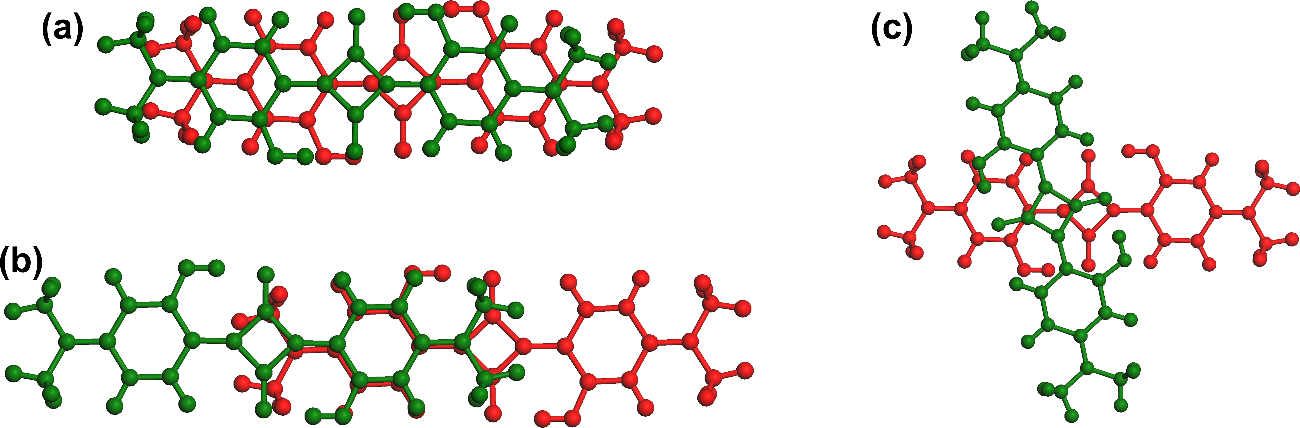


**Figure S47**. Example models of dimer configurations in which one monomer is translated or rotated with respect to the other monomer by different distances or angles, (a) DFT-optimized ground-state structure of dimer (denoted as dimer-X+0.16 nm or dimer-rotate+0°), (b) dimer configuration in which one monomer is translated with respect to the other monomer by 0.76 nm along the X axis (denoted as dimer-X+0.76 nm), and (c) dimer configuration in which one monomer is rotated clockwise with respect to the other monomer by 75° (denoted as dimer-rotate+75°). All models are in top view.


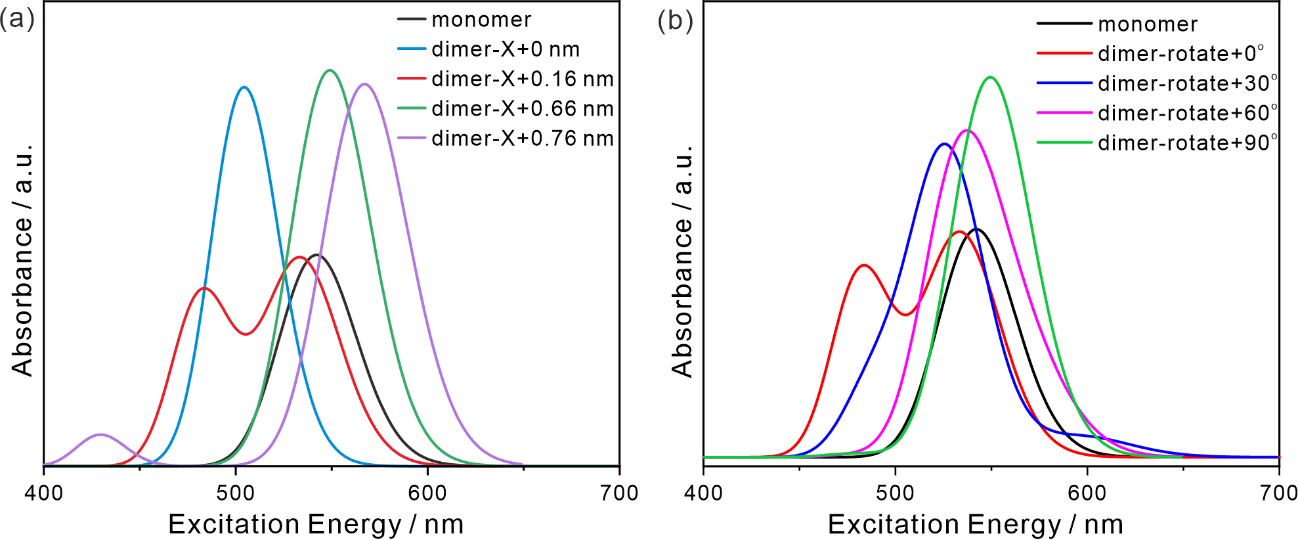


**Figure S48.** Calculated one-photon absorption spectra for SQ monomer and dimer configurations in which one monomer is (a) translated or (b) rotated with respect to the other monomer by different distances or angles (dimer-rotate+0° in **Figure S47**a is the geometrical optimized dimer-X+0.16 nm). Arbitrary units (a.u.) were used for the absorbance of broadened spectra.

**Table S9**. Calculated excitation energies *λ* (in nm) and oscillator strengths *f* (in atomic units, see numbers in brackets) of the three lowest excited states (S_1_, S_2_ and S_3_) of SQ monomer and dimer configurations with different slip distance along long molecular axis.

| Configuration | *λ*_S1_ (*f*_S1_) | *λ*_S2_ (*f*_S2_) | *λ*_S3_ (*f*_S3_) |
| --- | --- | --- | --- |
| monomer | 542 (1.85) | 342 (0) | 337 (0.14) |
| dimer-X+0 nm | 852 (0) | 512 (0.21) | 504 (3.14) |
| dimer-X+0.16 nm | 653 (0) | 534 (1.81) | 483 (1.52) |
| dimer-X+0.41 nm | 631 (0) | 543 (2.66) | 477 (0.66) |
| dimer-X+0.66 nm | 588 (0) | 549 (3.47) | 460 (0) |
| dimer-X+0.71 nm | 567 (0) | 558 (3.36) | 446 (0.18) |
| dimer-X+0.76 nm | 567 (3.35) | 544 (0) | 440 (0) |
| dimer-X+0.91 nm | 563 (3.71) | 541 (0) | 427 (0) |

**Table S10**. Calculated excitation energies *λ* (in nm) and oscillator strengths *f* (in atomic units, see numbers in brackets) of the three lowest excited states (S_1_, S_2_ and S_3_) of SQ monomer and dimer configurations in which one monomer is rotated with respect to the other monomer by different angles.

| Configuration | *λ*_S1_ (*f*_S1_) | *λ*_S2_ (*f*_S2_) | *λ*_S3_ (*f*_S3_) |
| --- | --- | --- | --- |
| monomer | 542 (1.85) | 342 (0) | 337 (0.14) |
| dimer-rotate+0° | 653 (0) | 534 (1.81) | 483 (1.52) |
| dimer-rotate+15° | 635 (0.04) | 529 (2.01) | 490 (1.26) |
| dimer-rotate+22.5° | 613 (0.09) | 528 (2.27) | 491 (0.84) |
| dimer-rotate+30° | 597 (0.17) | 527 (2.46) | 491 (0.22) |
| dimer-rotate+45° | 580 (0.40) | 528 (2.67) | 493 (0.10) |
| dimer-rotate+60° | 567 (0.75) | 534 (2.41) | 489 (0.02) |
| dimer-rotate+75° | 557 (1.16) | 541 (1.97) | 485 (0) |
| dimer-rotate+90° | 551 (1.50) | 548 (1.59) | 481 (0) |

## 9.3 Discussion

The calculated spectrum has three-peak form based on geometrical parameters such as *α* ~ 20^o^and *θ* ~70^o^, see **Table S10**, exhibits the global features as well as the tendency of peak shift and oscillators redistribution in line with experiments. According to our calculation, H-dimer with slip distance of 0.16 nm (denoted as dimer-X+0.16nm in **Table S9**) with angle *θ* ~70^o^ exhibits double-hump spectral signatures with similar oscillator redistribution and peak shift to that of experiments. Therefore, some slight tilt of molecules relative to the plane normal to the column axis exists in our case and still guarantee the optical negative nature of columns. Based on dimer of *θ* ~ 70^o^, spectra of different *α* are calculated, as shown in **Figure S47**c and **Table S10**. Calculated absorption profiles with small angle *α* (*α* < 20°) are in agreement with the spectra record at low LC temperature. Furthermore, increasing angle *α* further (*α* > 30°), those absorption profiles are close to that of monomers.

# 10. Additional discussion on phase sequence


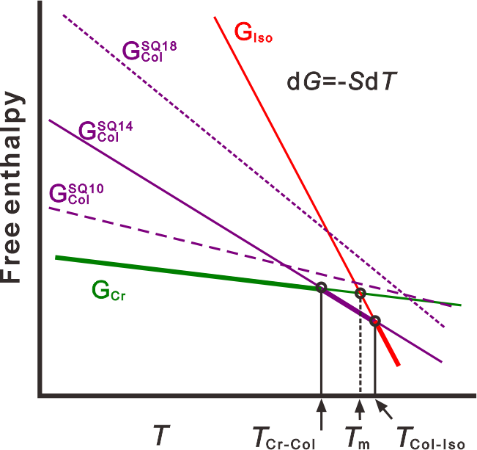


**Figure S49.**Schematic temperature dependence of free enthalpy of crystal (green), Col_hex_ (purple) and Iso (red) phases for the three SQ compounds indicated. G’s for the crystal and Iso are taken as reference, *T* for temperature.

As in almost all LCs, the simple shape of the rigid part (rod or disc) allows close packing and ensures low enthalpy *H*, whilst the flexible chains provide the entropy *S*. A stable enantiotropic LC phase forms if in a given temperature interval, when *G* = *H* – *TS* is the lowest. **Figure S49** illustrates qualitatively why **SQ10** and **SQ18** do not form the Col_hex_ phase, while **SQ14** does. Even though **SQ10** has the lowest enthalpy *H* of the three, and has the lowest Gibbs free energy *G* at low *T,* because of its short chains, entropy *S* is low too, hence its *G* does not drop sufficiently steeply on heating; recalling that d*G* = -*S*d*T*. In contrast, **SQ18** has ample entropy, but because of its low rigid fraction, its *H* value is the highest, and even the steepest descent of *G* does not bring it below *G_Cr_* below *T_m_*. Only for **SQ14** *H* is sufficiently low and *S* sufficiently high for its *G_Col_* to drop below both *G_Cr_* and *G_Iso_* in a certain temperature range.

# 11. References

S1. C. Zhan, J. Cheng, B. Li, S. Huang, F. Zeng, S. Wu, *Anal. Chem.* **2018**, *90*, 8807-8815.

S2. M. Yoshio, T. Mukai, H. Ohno, T. Kato, *J. Am. Chem. Soc.* **2004**, *126*, 994-995.

S3. A. Immirzi, B. Perini, *Acta Cryst.* **1977**, *A33*, 216-218.

S4. F. Liu, M. Prehm, X. Zeng, C. Tschierske, G. Ungar, *J. Am. Chem. Soc.* **2014**, *136*, 6846-6849.

S5 S. Freese, P. Lässing, R. Jakob, M. Schulz, A. Lützen, M. Schiek, N. Nilius, *Phys. Status Solidi B Basic Res* **2019,** *256* (3), 1800450.

S6 O. A. Mass, C. K. Wilson, G. Barcenas, E. A. Terpetschnig, O. M. Obukhova, O. S. Kolosova, A. L. Tatarets, L. Li, B. Yurke, W. B. Knowlton, R. D. Pensack, J. Lee, *J. Phys. Chem. C* **2022,** *126* (7), 3475-3488

S7 O. A. Mass, C. K. Wilson, S. K. Roy, M. S. Barclay, L. K. Patten, E. A. Terpetschnig, J. Lee, R. D. Pensack, B. Yurke, W. B. Knowlton, *J. Phys. Chem. B* 2020, 124 (43), 9636-9647

S8 H. P. Wagner, A. DeSilva, *Phys. Rev. B* **2004,** *70* (23), 235201.

S9 J. Seibt, P. Marquetand, V. Engel, Z. Chen, V. Dehm, F. Wurthner, *Chem. Phys.* **2006,** *328* (1-3), 354-362.

S10. L. Tan, Z. He, X. Wang, K. Jiang, *arxiv* **2024**,*https://arxiv.org/abs/2404.15363.*

S11. Z. He, X. Wang, P. Zhang, A.-C. Shi, K. Jiang, *Macromolecules* **2024**, *57*, 2154-2164.

S12. T. Yanai, D. P. Tew, N. C. Handy, *Chem. Phys. Lett.* **2004**, *393*, 51-57.

S13. S. Grimme, J. Antony, S. Ehrlich, H. Krieg, *J. Chem. Phys.* **2010**, 132, 154104.

S14. S. Ling, S. Schumacher, I. Galbraith, M. J. Paterson, *J. Chem. Phys. C* **2013**, *117*, 6889-6895.

S15. M. J. Frisch, G. W. Trucks, H. B. Schlegel, G. E. Scuseria, M. A. Robb, J. R. Cheeseman, G. Scalmani, V. Barone, B. Mennucci and G. A. Petersson, et al., *Gaussian 16 Rev. C.01*, Wallingford, CT, 2016.

S16. N. A. Montgomery, J.-C. Denis, S. Schumacher, A. Ruseckas, P. J. Skabara, A. Kanibolotsky, M. J. Paterson, I. Galbraith, G. A. Turnbull, I. D. W. Samuel, *J. Phys. Chem. A* **2011**, *115*, 2913-2919.

S17. J.-C. Denis, A. Ruseckas, G. J. Hedley, A. B. Matheson, M. J. Paterson, G. A. Turnbull, I. D. W. Samuel, I. Galbraith, *Phys. Chem. Chem. Phys.* **2016**, *18*, 21937-21948.
